# Global burden of knee osteoarthritis from 1990 to 2021: Trends, inequalities, and projections to 2035

**DOI:** 10.1371/journal.pone.0320115

**Published:** 2025-06-11

**Authors:** Junjie Chen, Xianshuai Chen, Tianshu Wang, Ming Li, Huanhuan Dai, Shuangshuang Shang, Lili Cheng, Zhongfu Tang, Sidi Liu, Chuanbing Huang

**Affiliations:** 1 The First Affiliated Hospital of Anhui University of Traditional Chinese Medicine, Hefei, China; 2 Department of Orthopaedics, Sports Trauma and Arthroscopic Surgery, Bozhou People’s Hospital, Bozhou, China; 3 Center for Xin’an Medicine and Modernization of Traditional Chinese Medicine of IHM, The First Affiliated Hospital of Anhui University of Chinese Medicine, Hefei, China; 4 College of Biology and Food Engineering of Hefei Normal University, Hefei, China; University College London, UNITED KINGDOM OF GREAT BRITAIN AND NORTHERN IRELAND

## Abstract

**Introduction:**

To present the global, regional, and national burden of knee osteoarthritis (KOA) and its causative factors, categorized by age, gender, and sociodemographic indices from 1990 to 2021, and to project future trends to 2035.

**Methods:**

A comprehensive analysis of KOA epidemiology was conducted using data from the 2021 Global Burden of Disease Study (GBD). The study examined change trends in KOA burden between 1990 and 2021, including prevalence, incidence, disability-adjusted life years (DALYs), and associated risk factors. Health inequality analyses were performed using slope index of inequality (SII) and concentration index (CI). Decomposition analysis was conducted to understand the contributions of population growth, aging, and epidemiological changes to the increasing burden. Future projections were made for global, Chinese, and Indian trends to 2035.

**Results:**

In 2021, the global prevalence of KOA was 374.7 million cases, with an annual incidence of 3.0846 million cases, totaling 12.01 million DALYs. Age-standardized rates for prevalence, incidence, and DALYs increased by 8.3%, 7.1%, and 8.2% respectively since 1990. Health inequality analyses revealed widening disparities across SDI levels, with SII for crude incidence rates increasing from 251 to 400 per 100,000 between 1990 and 2021. Decomposition analysis showed population growth as the primary driver of increased burden globally (75.07% for DALYs), with variations across SDI regions. Projections to 2035 indicate substantial increases in global burden, with incidence expected to rise by 33.6%, prevalence by 43.8%, and DALYs by 41.4%. China and India show differing patterns in projected burden increases.

**Conclusion:**

KOA remains a significant public health concern with increasing burden and widening health inequalities. The projected increases highlight the need for targeted interventions, especially in rapidly growing populations. Preventive measures should focus on reducing high BMI, implementing gender-specific treatments, and addressing regional disparities to mitigate the future burden of KOA.

## Introduction

Osteoarthritis (OA), a common and persistent arthropathy noted for the deterioration of articular cartilage, can gradually impacts whole joint structure [1]. Due to its unique physiological and structural characteristics, the knee joint is particularly susceptible to lesions, specifically knee osteoarthritis (KOA) [2]. The development and progression of this disease are closely linked to multiple risk factors, including obesity, knee injuries, muscle weakness, and joint laxity [3]. Given its chronic nature and high prevalence, KOA has imposed a heavy burden on patients and their family members and has exerted substantial pressure on healthcare systems and the community economy [4]. Although KOA is a chronic irreversible disease, efficient self-management practices can significantly reduce the disease burden and raise the standard of living for KOA individuals [5]. The Global Burden of Disease Study (GBD) is the sole detailed quantitative source of disability outcomes associated with disease and injury worldwide [6] and encompasses mortality and disability information for different countries, age groups, and genders (https://www.healthdata.org/GBD). GBD includes data on disease prevalence, incidence, and disability-adjusted life years (DALYs). According to GBD, OA cases globally reached 527.8 million in 2019 [7]. After reviewing the literature, it was found that most research focused primarily on OA. For instance, Cao et al. examined the trend and transnational inequality of the global OA burden between 1990 and 2019 through GBD [7]. Meanwhile, other scholars have explored OA burden in specific countries, such as India [8] and Indonesia, as conducted by Butarbutar JC et al. [9] However, Zhakhina G et al. [10] highlighted the KOA burden in Kazakhstan, which received less attention in terms of KOA-specific studies. Similarly, Shamekh A et al. [11] highlighted KOA burden in the Middle East. Nevertheless, there remains an insufficient amount of research regarding the KOA burden across 204 countries and regions worldwide from various perspectives, such as gender, age, and economic status.

In current work, the recent published data on KOA from the GBD in 2021 was meticulously examined and analyzed. This study aimed to deliver a thorough analysis of KOA prevalence, incidence, and DALYs across global, regional, and country scopes. Attention was paid to the impact of KOA on population health by comprehensively analyzing the burden of KOA by age, sex, and socio-demographic indices (SDIs) for 204 countries and territories between 1990 and 2021.Decomposition analyses were conducted to better understand the factors contributing to the rising burden of knee osteoarthritis (KOA) worldwide, including population growth, aging, and changes in disease prevalence. Additionally, health inequality analyses were carried out using the slope index of inequality (SII) and the concentration index (CI) to assess disparities in health outcomes, resource distribution, and disease prevention efforts across various regions. Furthermore, a Bayesian age-period-cohort analysis (BAPC) model with integrated nested Laplace approximation (INLA) was utilized to forecast KOA trends up to the year 2035 on a global scale, as well as in specific regions such as China and India. This approach provides valuable insights into the future trajectory of KOA and helps identify potential areas for intervention and improvement. These detailed estimates of the KOA burden not only deepen our understanding of the public health impact of the disease, but also highlight the importance of implementing effective preventive measures and optimizing the allocation of healthcare resources.

## Methods

This study accessed the GBD 2021 database on 2024/09/26 for data download and collation.GBD 2021 study provided burden estimates for 371 disorders and damages in 204 countries and areas from 1990 to 2021. Additionally, it offered pertinent data on 87 risk factors. These countries and regions were categorized into 21 distinct groups. The methodology employed in this study has been extensively documented in prior literature, enabling users to access both fatal and non-fatal estimations via publicly accessible online tools (https://vizhub.healthdata.org/gbd-compare/ and https://ghdx.healthdata.org/gbd-results-tool).

### Data acquisition and processing

KOA data was obtained from 21 regions in the GBD 2021 study database, which included global estimates from 1990 to 2021. The extracted data was processed and analyzed to determine age-standardized rates (ASRs) per 100,000 individuals, case counts for each age group, and percentage changes in ASRs between 1990 and 2021. The ASR and number of cases were reported, along with their associated 95% uncertainty intervals (UIs), all rounded to two decimal places. These uncertainty intervals were directly obtained from the GBD 2021 database, which computes them through a sampling process of 1,000 draws from the posterior distribution of each estimation step, accounting for sampling error, non-sampling error, and model uncertainty. This approach provides a comprehensive assessment of uncertainty throughout the analytical process. DALYs were calculated following the standard GBD methodology, which combines years of life lost (YLL) due to premature mortality and years lived with disability (YLD). For KOA, which primarily contributes to YLD rather than YLL, disability weights were applied based on the severity distribution of the condition. The GBD 2021 study used no age-weighting or time discounting in DALY calculations, ensuring comparability across different time periods and age groups.

### Geographical analysis

The data from the GBD study covered estimates from 204 countries, 21 regions, and the global level. For each location, the ASR per 100,000 people in 1990 and 2021 was extracted, as well as the number of cases in each age group. ASRs were rounded to two decimal places, while the case numbers were presented as integers, both accompanied by their respective 95% UIs. Percentage changes in ASR from 1990 to 2021 were calculated, and the data were reshaped and sorted according to a predefined regional order. Case numbers were converted to millions and rounded to three decimal places. Final results, including ASR, percent change, and number of cases per site (in millions), stratified by measure, were exported.

### Gender-specific analysis

In order to investigate gender-specific trends in depth, GBD data was analyzed for males and females. ASRs per 100,000 people were obtained for 1990 and 2021, as well as the number of cases in each age group. ASRs were rounded to two decimal places and case numbers were expressed as integers, both accompanied by a 95% UI. We also calculated the percentage change in ASR from 1990 to 2021 and rounded to one decimal place. ASRs were rounded to two decimal places and case numbers were expressed as integers, both accompanied by a 95% UI. Percentage change in ASR from 1990 to 2021 was calculated and rounded to one decimal place. Case numbers were converted to millions, primary values were rounded to three decimal places, and the UI showed valid numbers.

### Mapping and visualization of global prevalence, incidence, and DALYs

Age-standardized KOA prevalence rates for 2021 were extracted from the GBD database and mapped to corresponding geographic regions. Disease prevalence was represented using a quartile-based color scheme and a 10-level spectral palette to create a primary world map with seven detailed sub-maps (including the Caribbean and Central America, the Persian Gulf, Southeast Asia, West Africa, Eastern Mediterranean, and Northern Europe) arranged next to it.

### Age and gender-specific analysis of global disease burden

A detailed analysis of the GDB data for the 2021 KOA was conducted, focusing on 20 age groups, stratified by gender. Ages ranged from 1–4 years to 95 + years. We calculated the number of cases (in millions) and incidence rates per 100,000 people based on prevalence, number of deaths, and disability-adjusted life years. For each measure, a combined bar and line graph was created, where the bars represent the number of cases and the dashed line shows the rate. Error bars and confidence intervals are used to illustrate the uncertainty of the data.

### SDI and disease burden correlation

The association between SDI and KOA disease burden was investigated. Data from GBD 2021 were combined with SDI data for global and regional analysis. Scatter plots were generated for each indicator, including prevalence, number of deaths, and DALYs, which were compared with SDI. Different colors and shapes were used to distinguish various regions. LOESS smoothing was used to visualize trends and Spearman’s correlation test was used to quantify the relationship between the SDI and the disease burden indicators.

### Decomposition analysis

To gain insight into the factors contributing to changes in DALYs in the KOA from 1990 to 2021, this study disaggregates analyses by sex as well as by age distribution, population size, and epidemiological changes. First, the DALYs in the KOA were decomposed into subgroups by sex (female and male). In addition, to investigate how much population growth, ageing and epidemiological changes have influenced epidemiological changes in the KOA over the last 30 years, we decomposed the DALYs by population, age structure, and population- and age-standardised DALY rates (which we define here as epidemiological changes) [12].

### Cross-country inequality analysis and predictive analysis

Cross-country inequality analysis refers to the analysis of differences in the burden of disease, health outcomes and their correlates between different countries or regions, with the aim of quantifying and comparing the level of health, distribution of resources, and effectiveness of disease prevention between countries, in order to identify inequalities in global health. And to further improve relevant policies, programmes and practices to reduce disparities in health distribution. In this study, the Inequality Slope Index and the Concentration Index were used to measure the distribution of the KOA burden across countries [13].

The above analyses focused on the burden of KOA in the past decades, and this study used a Bayesian Age-Period-Cohort Analysis (BAPC) model with Integrated Nested Laplace Approximation (INLA) to make further projections of the future burden of KOA [14]. The BAPC model was selected over alternative forecasting approaches such as ARIMA or machine learning models due to its ability to simultaneously account for age, period, and cohort effects, which are crucial in chronic disease epidemiology like KOA where demographic transitions significantly influence disease burden. The Bayesian framework allows for incorporation of parameter uncertainty and provides credible intervals for projections, while the INLA approach offers computational efficiency while maintaining accuracy in Bayesian inference. To validate the model’s performance, we conducted a comprehensive comparison between the model’s predictions and actual observed data from 1990–2021 for all burden metrics. This validation analysis yielded exceptional results with R-squared values of 0.999 across all measures (DALYs, Prevalence, and Incidence), locations (Global, China, and India), and metric types. The Mean Absolute Percentage Error (MAPE) values were extremely low, ranging from 0.0000255% to 0.00254%, demonstrating the model’s remarkable accuracy. Additionally, Mean Absolute Error (MAE) and Root Mean Square Error (RMSE) metrics were calculated, showing consistently low values that further confirm the model’s precision.

### Software and packages

Throughout the entire research process, the following R packages were extensively utilized:

Data manipulation and analysis: dplyr, tidyr, stringr, and arrow.
Data visualization: ggplot2, ggmap, rgdal, RColorBrewer, patchwork, and ggrepel.
Geospatial analysis: rgdal.
Statistical analysis: stats.
File output: writexl.

These software packages were employed for various tasks, including data cleaning, reshaping, statistical computations, geographical mapping, correlation analyses, and creating complex visualizations.

### Patient and public involvement

Since this study was conducted based on a publicly available dataset, it received an exemption from the Ethics Committee of the First Affiliated Hospital of Anhui University of Traditional Chinese Medicine.

## Results

### Global level

The 2021 worldwide KOA prevalence reached 374.7 million cases, with an age-standardized prevalence rate of 4,294.27 cases per 100,000 people, an increase of 8.3% from 1990. In addition, there will be 3.0846 million new cases of KOA in 2021, with an age-standardized prevalence rate of 353.67 cases per 100,000 people, an increase of 7.1% from 1990. The worldwide number of DALYs attributed to KOA reached 12.01 million, corresponding to an age-standardized DALY rate of 137.59 per 100,000 people, reflecting an 8.2% elevation starting from 1990 (Table 1).

**Table 1:** KOA prevalence, incidence, and DALYs in 2021, with percentage changes in ASRs per 100,000 population by GBD, between 1990 and 2021.

	Prevalence (95% UI)			Incidence (95% UI)			DALYs (95% UI)		
**Location**	**No, in** **millions** **(95% UI)**	**ASR s per** **100 000** **(95% UI)**	**Percentage** **change in** **ASR s from** **1990–2021**	**No, in** **millions** **(95% UI)**	**ASR s per** **100 000** **(95% UI)**	**Percentage** **change in** **ASR s from** **1990–2021**	**No, in** **millions** **(95% UI)**	**ASR s per** **100 000** **(95% UI)**	**Percentage** **change in** **ASR s from** **1990–2021**
Global	374.739(322.000,428.000)	4294.27 (3695.04,4910.76)	8.3 (8.3,8.3)	30.846(26.500,35.200)	353.67 (304.56,402.5)	7.1 (7.1,7.1)	12.019(5.860,23.300)	137.59 (67.08,266.87)	8.2 (7.9,8)
High-income Asia Pacific	22.54(19.600,25.600)	5573.73 (4841.29,6334.33)	2.9 (2.8,3.4)	1.483(1.310,1.690)	458.22 (397.65,522.59)	3.8 (3.6,4)	0.722(0.354,1.450)	180.61 (88.02,356.06)	3.2 (3.4,4.3)
High-income North America	29.009(25.000,33.400)	4709.03 (4074.67,5394.69)	4.6 (4.6,5.3)	2.104(1.830,2.430)	389.08 (335.14,444.02)	5.9 (5.4,5.8)	0.912(0.450,1.810)	148.92 (73.19,294.55)	3.5 (3.5,3.7)
Western Europe	35.172(30.600,40.200)	4169.47 (3611.88,4769.26)	6 (5.9,6.1)	2.555(2.240,2.950)	357.68 (311.46,406.98)	6.9 (6.5,7.5)	1.119(0.553,2.230)	133.93 (65.73,263.33)	6.1 (6.2,6.2)
Australasia	2.389(2.070,2.740)	4808.67 (4147.42,5488.06)	12.1 (10.7,12.8)	0.177(0.153,0.205)	403.34 (348.15,463.45)	12.3 (12,12.5)	0.076(0.0377,0.153)	154.18 (75.68,309.09)	12.3 (11.8,13.7)
Andean Latin America	2.758(2.370,3.160)	4588.33 (3950.43,5244.03)	12.7 (12.4,12.5)	0.243(0.209,0.279)	386.9 (333.29,443.77)	11.1 (10.7,11.9)	0.089(0.0432,0.172)	147.55 (72.02,286.28)	12.5 (11.2,12)
Tropical Latin America	11.649(10.000,13.300)	4455.51 (3838.66,5095.51)	11.3 (11.1,11.4)	1.015(0.871,1.160)	380.8 (327.9,434.95)	9.9 (9.5,10.1)	0.369(0.181,0.713)	141.07 (69.25,272.84)	11.3 (10.5,11.4)
Central Latin America	11.499(9.890,13.100)	4492.79 (3881.3,5124.51)	9.2 (9.3,9.3)	1.014(0.871,1.160)	383.48 (330.54,435.99)	8.2 (7.7,8.2)	0.368(0.180,0.712)	143.61 (70.3,278.58)	9.3 (9.3,9.4)
Southern Latin America	3.943(3.420,4.500)	4617.51 (4009.6,5266.68)	11.9 (11.8,12.2)	0.316(0.274,0.361)	387.34 (336.02,441.16)	11.1 (10.5,11.6)	0.126(0.062,0.250)	148.13 (72.64,291.9)	11.5 (10.9,12.1)
Caribbean	2.404(2.070,2.740)	4454.36 (3840.02,5084.92)	9.2 (8.5,8.7)	0.2(0.173,0.230)	374.83 (324.54,430.56)	7.5 (8,8.5)	0.077(0.038,0.151)	142.84 (70.48,281.03)	8.5 (9.7,9.8)
Central Europe	6.775(5.840,7.840)	3230.75 (2788.51,3718.9)	7.5 (7.8,8)	0.529(0.459,0.612)	282.04 (242.21,323.09)	6.9 (6.7,6.9)	0.215(0.106,0.422)	103.21 (50.75,200.27)	7.9 (7.4,7.7)
Eastern Europe	11.763(10.100,13.500)	3409.25 (2919.69,3923.46)	8.1 (7.6,8.9)	0.958(0.824,1.110)	298.56 (256.44,342.29)	7.3 (6.8,7)	0.373(0.183,0.722)	108.31 (53.24,209.35)	8.1 (6.8,7.7)
Central Asia	2.301(1.970,2.660)	2700.22 (2336.33,3105.34)	6.6 (6.7,6.9)	0.222(0.189,0.256)	239.29 (205.97,274.09)	6.1 (5.7,6.5)	0.074(0.0366,0.143)	86.71 (42.6,169.71)	6.4 (7.3,7.7)
North Africa and Middle East	18.592(16.000,21.400)	3810.43 (3275.1,4375)	12.8 (12.5,12.7)	1.812(1.540,2.080)	325.34 (279.63,371.94)	11.3 (11.3,11.3)	0.597(0.290,1.150)	121.33 (59.3,235.41)	12.1 (11.8,12.2)
South Asia	58.791(50.300,67.100)	3818.04 (3282.09,4349.41)	10.9 (10.8,11.1)	5.405(4.660,6.150)	324.2 (280.14,369.04)	9.5 (9.6,9.7)	1.872(0.914,3.590)	120.81 (59.17,232.43)	11.3 (10.6,11.2)
Southeast Asia	22.707(19.300,26.200)	3238.01 (2773.46,3719.63)	12.2 (11.8,12.7)	2.051(1.740,2.370)	274.07 (235.74,314.07)	10.7 (10.6,11.1)	0.737(0.357,1.410)	104.35 (50.89,201.33)	12.4 (12,12.1)
East Asia	113.376(96.000,131.000)	5016.78 (4267.39,5758.88)	7.6 (6.9,7.5)	8.8(7.530,10.200)	406.19 (348.6,466.86)	7.6 (7.5,7.6)	3.678(1.770,7.080)	162.47 (78.29,314.12)	7.5 (7.4,7.8)
Oceania	0.339(0.286,0.391)	4025.21 (3433.77,4602.96)	7.9 (7,7.3)	0.034(0.0285,0.0391)	334.57 (286.05,387.71)	7 (6.1,7.8)	0.011(0.00525,0.0213)	128.75 (62.39,250.96)	7.6 (7.3,9)
Western Sub-Saharan Africa	8.097(6.880,9.290)	3801.12 (3250.23,4369.9)	8.7 (8.5,8.6)	0.832(0.706,0.954)	326.72 (280.96,375.13)	8.1 (7.9,8.3)	0.262(0.126,0.497)	121.72 (59.6,234.01)	9.2 (8.5,9.2)
Eastern Sub-Saharan Africa	6.219(5.300,7.140)	3446.18 (2959.9,3965.07)	7.1 (6.9,7)	0.652(0.555,0.748)	299.96 (257.48,343.27)	6.6 (6.3,6.4)	0.201(0.098,0.381)	110.05 (54.14,212.29)	7.5 (6.6,8)
Central Sub-Saharan Africa	2.054(1.760,2.360)	3433.3 (2947.25,3926.7)	4.4 (4.3,4.7)	0.218(0.186,0.248)	297.77 (256.63,338.31)	4 (2.7,4.4)	0.066(0.0321,0.128)	109.23 (53.33,213.55)	4.9 (4.7,5.3)
Southern Sub-Saharan Africa	2.364(2.020,2.720)	3913.26 (3363.99,4509.41)	7.9 (7.8,8.2)	0.228(0.195,0.261)	338.1 (290.72,388.21)	7.3 (6.8,7.8)	0.075(0.0366,0.145)	123.64 (60.67,240.41)	6.7 (6.6,6.8)

### Regional level

In 2021, the regions with the greatest age-standardized KOA prevalence per 100,000 individuals were high-income Asia Pacific (5,573.73), East Asia (5,016.78), and Australasia (4,808.67). Conversely, the minimum prevalence rates were found in Central Asia (2,700.22), Central Europe (3,230.75), and Southeast Asia (3,238.01) (Table 1). Regarding incidence rates, the top age-standardized incidence rates of KOA were assessed in high-income Asia Pacific (458.22), East Asia (406.19), and Australasia (403.34), whereas the smallest incidence rates were found in Central Asia (239.29), Southeast Asia (274.07), and Central Europe (282.04). In 2021, the top age-standardized DALY rates were found in high-income Asia Pacific (180.61), East Asia (162.47), and Australasia (154.18), while Central Asia (86.71), Central Europe (103.21), and Southeast Asia (104.35) demonstrated the lowest DALY rates (Table 1). S1–S3 Figs in S1 File depict the age-standardized KOA prevalence, incidence, and DALY rates stratified by gender. Notably, although women exhibited greater KOA prevalence, incidence, and DALYs than men, the percentage change in prevalence among men was greater than that among women from 1990 to 2021, especially in Africa, America, Australasia, and Western Europe.

From 1990 to 2021, the most notable elevations in age-standardized KOA prevalence were found in North Africa and the Middle East (12.8%), Andean Latin America (12.7%), and Southeast Asia (12.2%). The smallest increases were noted in high-income Asia Pacific (2.9%), central sub-Saharan Africa (4.4%), and high-income North America (4.6%) (Table 1). Age-standardized incidence increased in every region, with maximum rises observed in Australasia (12.3%), North Africa and the Middle East (11.3%), and Southern and Andean Latin America (11.1%). Age-standardized DALY rates rose across all regions, including Andean Latin America (12.5%), Southeast Asia (12.4%), and Australasia (12.3%) (Table 1). S4–S6 Figs in S1 File illustrate the age-standardized KOA prevalence, incidence, and DALY rates by gender (1990–2021).

From 1990 to 2021, the prevalence of KOA cases rose from 159.8 million to 374.7 million, with East Asia, Western Europe, and South Asia showing the highest prevalence both in 1990 and by 2021 (S1 Table in S1 File). Additionally, KOA incidence increased from 14.1 million to 30.8 million, with East Asia, South Asia, and Western Europe leading in 2021 (S2 Table in S1 File).

The DALYs attributed to KOA rose from 5.14 million to 12.01 million between 1990 and 2021, with East Asia, South Asia, and Western Europe continuing to report the top DALY counts in 2021 (S3 Table in S1 File).

### National level

**Fig 1:**
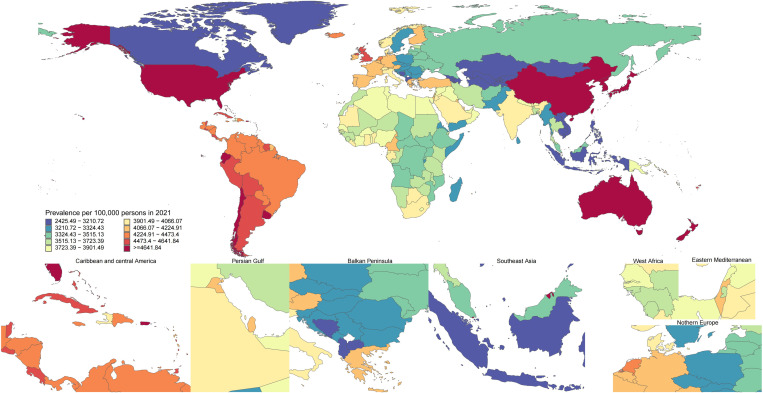
Age-standardized KOA prevalence per 100,000 individuals in 2021, by country. (Note: The maps were created using the R language and open source software packages and data.).

**Fig 2:**
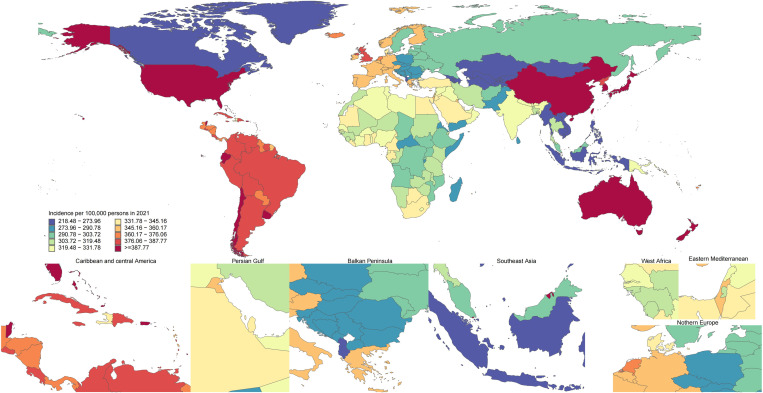
Age-standardized KOA incidence per 100,000 individuals in 2021, by country. (Note: The maps were created using the R language and open source software packages and data.).

The age-standardized prevalence of KOA in 2021 ranged from 2425.49 to 6201.62 cases per 100,000 people. The highest prevalence rates were found in the Republic of Korea (6201.62), Brunei Darussalam (5824.76), and Singapore (5810.72), and the lowest prevalence rates were found in Tajikistan (2425.49), Kyrgyzstan (2596.13), and Mongolia (2600.41) (Fig 1, and S1 Table in S1 File). The age-standardized KOA incidence in China varied from 218.48 cases to 491.74 cases per 100,000 individuals. The maximum incidence rates were found in the Republic of Korea (491.74), Brunei Darussalam (468.07), and Singapore (467.22), while the minimum was found in Tajikistan (218.48), Kyrgyzstan (231.57), and Mongolia (231.84) (Fig 2, and S2 Table in S1 File). Additionally, the age-standardized DALY rate for KOA nationwide varied from 78.21 to 199.93 patients per 100,000 individuals. Among them, the Republic of Korea (199.93), Singapore (189.49), and Brunei Darussalam (186.75) had the highest incidence rates, while Tajikistan (78.21), Mongolia (83.38), and Kyrgyzstan (83.75) reported the minimum DALY rates (S7 Fig and S3 Table in S1 File).

The percentage change in age-standardized prevalence between 1990 and 2021 varies significantly by country. Oman (21.71%), Equatorial Guinea (19.24%), and Thailand (18.1%) experienced the largest growth, while the Republic of Korea (0.67%), Japan (0.93%), and Burundi (1.83%) had the smallest growth (S1 Table in S1 File). Over the same timeframe, the age-standardized incidence rate also exhibited notable growth, with Oman (18.74%), Equatorial Guinea (16.59%), and Thailand (16.35%) showing the largest increases, while Burundi (1.66%), the Democratic Republic of the Congo (1.97%), and Japan (2.29%) had the smallest increases (S2 Table in S1 File). Regarding growth in the age-standardized DALY rates for KOA, Oman (21.62%), Equatorial Guinea (20.05%), and Thailand (18.57%) had the highest growth rates. In comparison, the Republic of Korea (1.02%), Japan (1.29%), and Burundi (1.83%) exhibited the minimum growth rates (S3 Table in S1 File).

### Age and sex patterns

**Fig 3:**
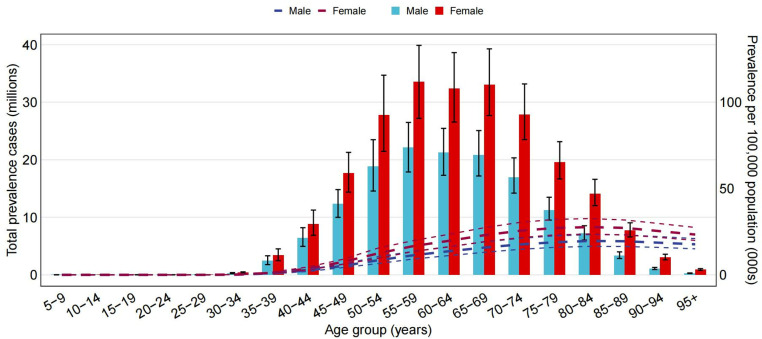
Global prevalence cases and KOA prevalence per 100,000 individuals, categorized by age and gender in 2021. The lines represent the prevalent cases along with 95% UIs for both sexes.

In 2021, there were no reported cases of KOA in individuals younger than 30. The worldwide KOA prevalence began to rise in 35–39 age group and peaked in 80–84 age group. Notably, the 65–69 age group recorded the highest number of cases, followed by a slight decline with age. The data showed that KOA was more common among women of all ages (Fig 3).

In 2021, the worldwide KOA incidence rate reached its peak in 55–59 age group, with a similarly high incidence in the 50–54 age group among both sexes, before declining with increasing age (S8 Fig in S1 File).

The overall DALY rate for KOA peaked in 75–79 age group and subsequently declined with age. The DALY rate among females surpassed that of males in all age groups. Furthermore, DALY number reached its peak in 55–59 age group, with females consistently recording higher numbers than males in each group (S9 Fig in S1 File).

### Association with the SDI

**Fig 4:**
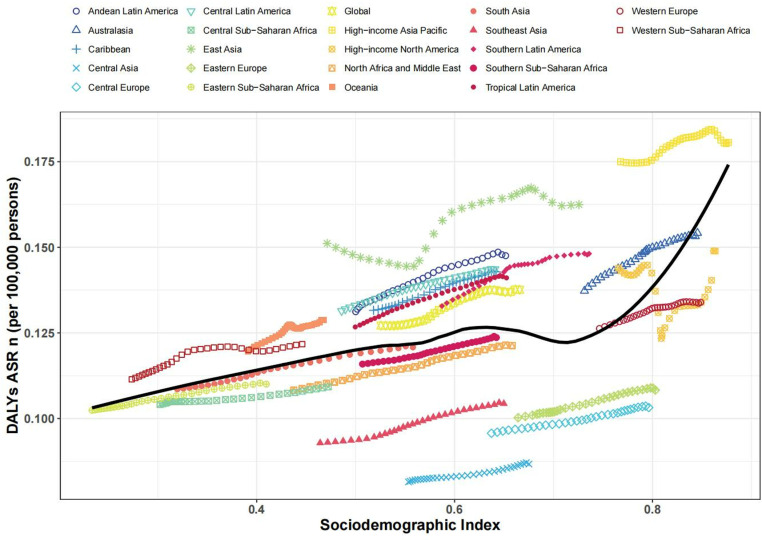
Age-standardized KOA DALY rates for the 21 GBD regions based on SDI,1990-2021. (Each region is represented by thirty data points, which depict the observed KOA DALY rates. The solid line signifies the anticipated values derived from SDI and disease rates across all locations. Regions that are above the solid line denote a greater burden of KOA than expected, while those beneath the line reflect a lesser burden than anticipated.).

On a regional scale, there was a generally positive correlation between SDI and age-standardized DALY rates for KOA between 1990 and 2021. As SDI rose, the DALY rate also increased accordingly, reaching its peak at around 0.6, before decreasing again at 0.7, and then showing an upward trend once more. Based on SDI from 1990 to 2021, the DALY rates in the following regions were higher than expected: high-income Asia Pacific, East Asia, Andean Latin America, Central Latin America, the Caribbean, Southern sub-Saharan Africa, Southern Latin America, Oceania, and Western sub-Saharan Africa. In contrast, the KOA burdens in Southern sub-Saharan Africa, North Africa and the Middle East, Central sub-Saharan Africa, South East Asia, Eastern Europe, Central Asia, and Central Europe were below expectations (Fig 4).

At a national level, KOA burden escalated with socioeconomic development in 2021, reaching an SDI of about 0.6. However, it then declined to an SDI of about 0.71 before rising again (S10 Fig in S1 File). As shown in S10 Fig, the KOA burdens in countries and territories such as South Korea, Singapore, and Brunei far exceeded expectations, while the KOA burdens in Tajikistan, Mongolia, and Kyrgyzstan were below expectations.

### Hazard factors

**Fig 5:**
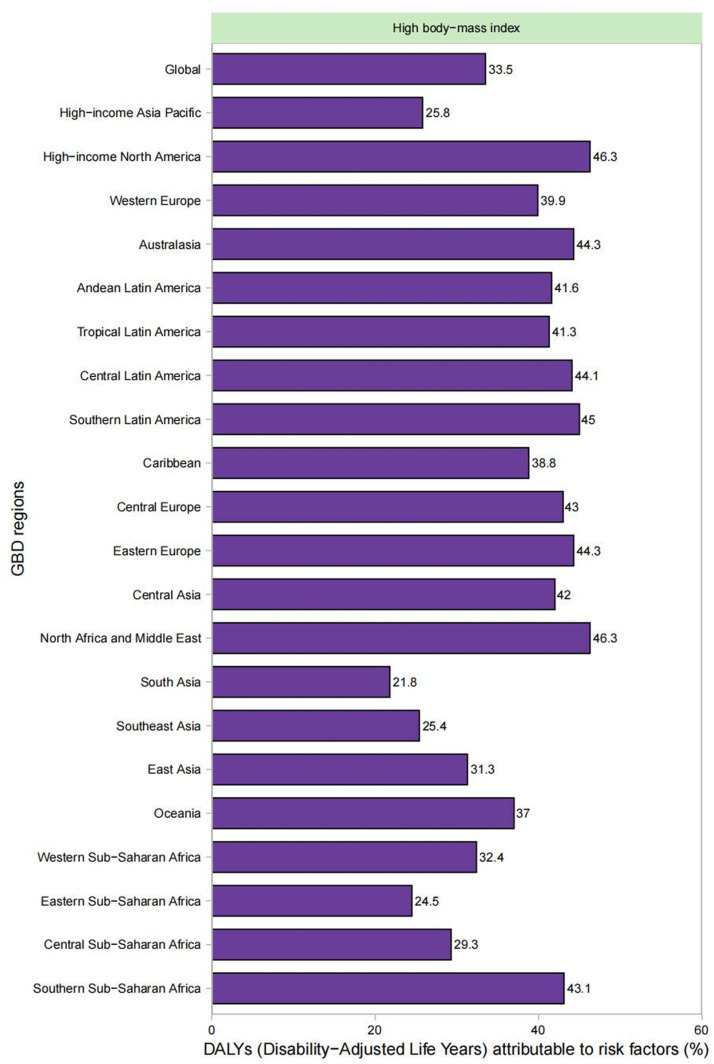
Percentage of KOA DALYs attributable to high BMI risk factor across the 21 GBD regions in 2021.

Among global KOA DALYs, high body mass index (BMI) constituted 33.5% (Fig 5). This proportion varied by region, and was higher among women (46.9%) globally. High-income North American men exhibited the maximum proportion of KOA DALYs linked to high BMI (46.9%), while women in North Africa and the Middle East showed the top proportion (48.7%) (S11 and S12 Figs in S1 File).

The proportion of KOA DALYs correlated with individual hazard factors varied across age groups, with the proportion linked to high BMI increasing with age. It peaked in the 60–64 age group at 34.7% (S13 Fig in S1 File). Our results indicated that males had the highest proportion (32.3%) in the 55–59 age group, while females had the highest proportion (36.4%) in the 60–64 age group (S14 and S15 Figs in S1 File).

### Health inequality analysis of knee osteoarthritis: Trends from 1990 to 2021

**Fig 6:**
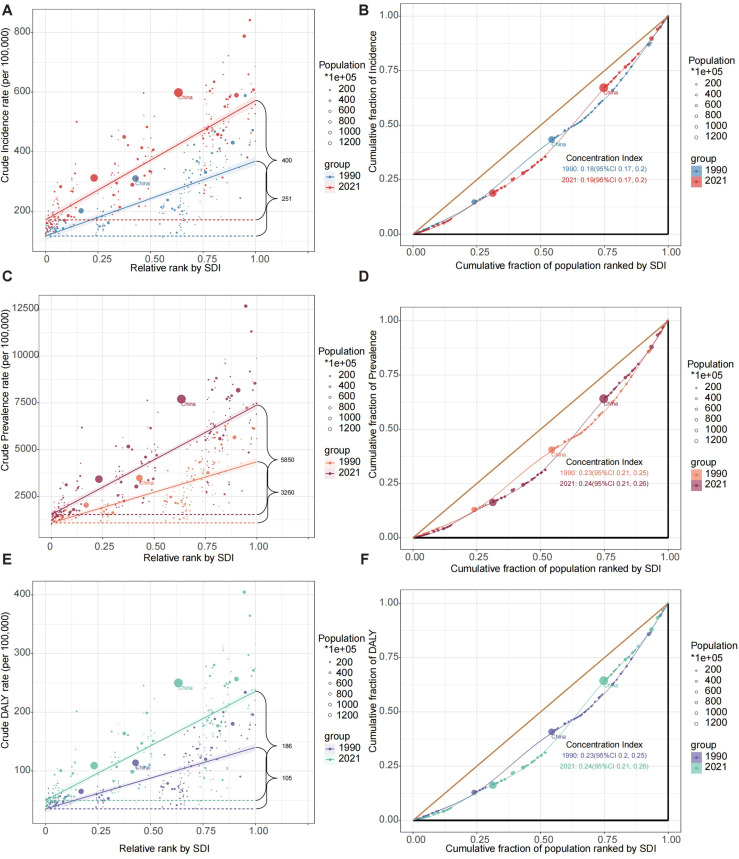
SDI-related health inequality regression and concentration curves for global KOA DALYs in 1990 and 2021.

Health inequality analyses for knee osteoarthritis revealed significant disparities across socio-demographic index (SDI) levels from 1990 to 2021 (Fig 6). The slope index of inequality (SII) for crude incidence rates increased from 251 per 100,000 in 1990–400 per 100,000 in 2021 (Fig 6A), indicating a widening gap between high and low SDI regions. The concentration index (CI) for incidence slightly increased from 0.18 (95% CI: 0.17, 0.20) to 0.19 (95% CI: 0.17, 0.20) over the same period (Fig 6B), suggesting a persistent concentration of incidence in higher SDI regions. For prevalence, the SII showed a substantial increase from 3,260 per 100,000 in 1990–5,850 per 100,000 in 2021 (Fig 6C), while the CI marginally increased from 0.23 (95% CI: 0.21, 0.25) to 0.24 (95% CI: 0.21, 0.26) (Fig 6D). The DALY rates exhibited similar trends, with the SII rising from 105 per 100,000 in 1990–186 per 100,000 in 2021 (Fig 6E), and the CI increasing slightly from 0.23 (95% CI: 0.20, 0.25) to 0.24 (95% CI: 0.21, 0.26) (Fig 6F). These results collectively demonstrate that the burden of knee osteoarthritis has not only increased over time but has also become more unequally distributed, with higher SDI regions bearing a disproportionately larger share of the disease burden. The observed changes in inequality measures over time were statistically significant (*P* < 0.05) based on bootstrapped confidence intervals, indicating that these trends represent meaningful shifts in the global distribution of KOA burden rather than random fluctuations.

### Decomposition analysis of global and regional trends in knee osteoarthritis burden

**Fig 7:**
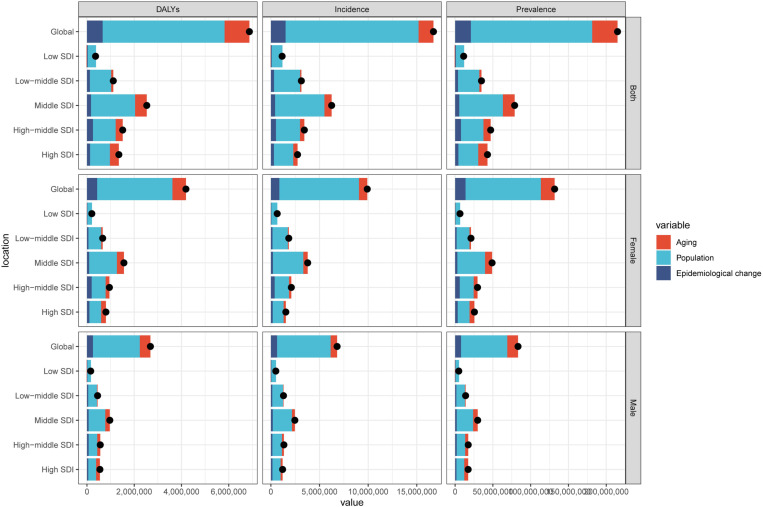
Changes in KOA DALYs by SDI quintile and sex subgroup, globally, from 1990 to 2021, based on aging, population growth, and epidemiological changes. Black dots indicate the total value of the change due to all three components.

Globally, the burden of knee osteoarthritis showed a substantial increase from 1990 to 2019. The overall difference in DALYs was 6,873,731.2, with population growth being the primary driver, accounting for 75.07% (5,160,219.61) of the increase. Aging contributed 15.29% (1,050,846.2), while epidemiological changes accounted for 9.64% (662,665.4) of the increase (Fig 7). It’s important to note that these factors are not entirely independent but interact with each other. For instance, aging populations often experience different epidemiological patterns due to changes in healthcare access, lifestyle modifications, and comorbidity profiles. Furthermore, the impact of population growth may vary across different age structures, potentially amplifying or mitigating the effects of aging on disease burden. The prevalence of knee osteoarthritis increased by 214,939,834.7 cases, with population growth again being the main factor (74.72%, 160,596,268.51 cases), followed by aging (15.6%, 33,523,206.1 cases) and epidemiological changes (9.69%, 20,820,360.07 cases). Incidence rates showed a similar pattern, with an overall increase of 16,711,606.9 cases, primarily driven by population growth (81.82%, 13,672,709.47 cases).

Across the five SDI regions, notable variations were observed. In the High SDI region, aging played a more significant role, contributing 28.11% (379,237.36) to the increase in DALYs, while population growth accounted for 62.27% (840,171.06). Conversely, in the Low SDI region, population growth was the dominant factor, responsible for 97.28% (353,734.76) of the DALY increase, with aging actually showing a negative contribution of −5.29% (−19,246.82). The Middle SDI region demonstrated patterns closer to the global average, with population growth contributing 73.74% (1,865,829.86) to the DALY increase. The High-middle SDI region showed the highest contribution from epidemiological changes at 16.76% (253,424.5) for DALYs. The Low-middle SDI region had a significant contribution from population growth at 81.03% (902,763.87) for DALYs, with epidemiological changes accounting for 11.01% (122,668.19) of the increase.

### Projected burden of knee osteoarthritis: Global, chinese, and indian trends to 2035

**Fig 8:**
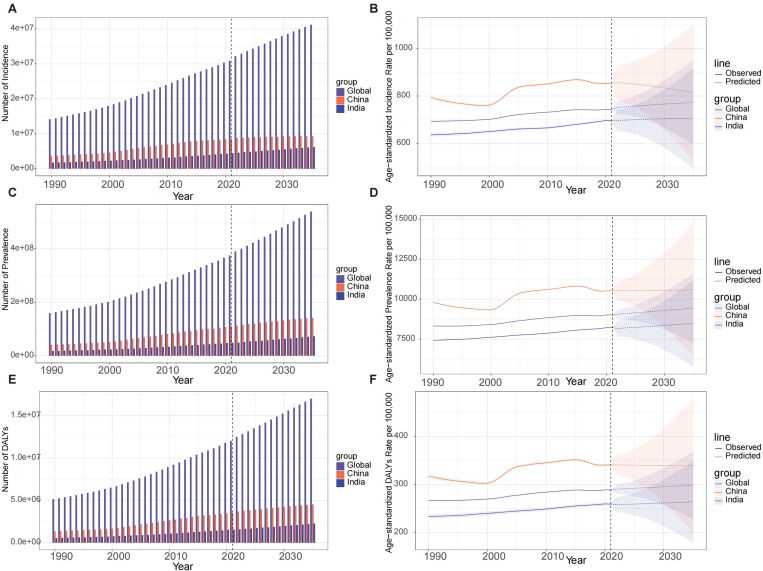
(A) number of incidence cases by 2035; (B) projected ASR for incidence from 2035; (C) number of prevalence cases by 2035; (D) projected ASR for prevalence by 2035; (E)number of cases by DALYs by 2035; (F) projected ASR for DALYs by 2035.

The burden of knee osteoarthritis is projected to increase substantially by 2035, as illustrated in Fig 8. Globally, the incidence is expected to rise from 30,845,727 cases in 2021–41,216,656 cases in 2035 (95% CI: 39,124,823−43,308,489), an increase of 33.6% compared to 2021 (Fig 8A and 8B). The prevalence is forecasted to grow from 374,738,652 cases to 538,765,346 cases, a 43.8% increase compared to 2021 (Fig 8C and 8D). DALYs are predicted to escalate from 12,019,010–16,992,687, a 41.4% increase (Fig 8E and 8F). In China, the incidence is projected to increase from 8,512,366–9,287,912 cases (9.1% increase), while prevalence is expected to rise from 109,575,349–141,619,364 cases (29.2% increase), and DALYs from 3,554,045–4,510,879 (26.9% increase). India is forecasted to experience a more rapid increase, with incidence rising from 4,416,593–6,231,760 cases (41.1% increase), prevalence from 48,463,944–72,402,620 cases (49.4% increase), and DALYs from 1,540,472–2,264,107 (47.0% increase). Notably, while the age-standardized rates (ASR) for incidence in China are projected to decrease slightly from 853.4 to 813.9 per 100,000, the global and Indian ASRs are expected to increase. This suggests that population growth and aging will be significant drivers of the increased burden, particularly in India and globally, while in China, demographic changes may play a more prominent role than epidemiological factors in the projected increase of knee osteoarthritis burden.

## Discussion

Utilizing data from the 2021 GBD, age-standardized rates of the population across 204 countries and regions was obtained and a detailed discussion on the most recent information regarding KOA prevalence, incidence, and DALYs from 1990 to 2021 was provided. GBD 2021 was collected and integrated more detailed data, providing the latest estimates of KOA epidemiology and enhancing data reliability by updating data sources. This study revealed a continuous rise in the overall global burden of KOA. The number of prevalence cases rose significantly from 159.8 million in 1990 to 374.7 million in 2021, reflecting a staggering growth rate of 134.48%. Similarly, incidence cases escalated from 14.13 million to 30.84 million, displaying a growth rate of 118.26%. Additionally, the number of KOA DALYs rose from 5.14 million to 12.01 million, representing an increase of 133.65%. Notably, there are substantial disparities in the prevalence of KOA among various geographical regions. This trend may provide important guidance for researchers and policymakers, helping clinicians and healthcare systems stratify management for different groups.

At the regional level, the age-standardized KOA prevalence, incidence, and DALY rates were highest in high-income Asia-Pacific region in 2021. Regarding gender differences, these indicators among women exceeded those of men globally and in most regions. However, it is noteworthy that in Southern Sub-Saharan Africa, these indicators showed that males were higher than females. This may be a phenomenon reported for the first time. From 1990 to 2021, East Asia had the maximum KOA prevalence, incidence, and DALYs. During this period, North Africa and the Middle East experienced the most significant elevation in age-standardized KOA prevalence, at 12.8%; Australasia saw the highest rise in age-standardized KOA incidence, reaching 12.3%; and Andean Latin America recorded the top elevation in age-standardized DALY, at 12.5%. Although the age-standardized KOA prevalence, incidence, and DALYs in women exceeded those in men, the percentage change in prevalence rate among men was greater than among women from 1990 to 2021, particularly in Africa, America, Australasia, and Western Europe. This finding indicated that the incidence rate in the male population needed continuous monitoring in the future.

In 2021, the national age-standardized prevalence of KOA varied from 2,425.49 to 6,201.62 cases per 100,000 individuals. The Republic of Korea emerged as the country with the highest prevalence rate, recording 6,201.62 cases per 100,000 individuals. Additionally, the Republic of Korea also topped the list regarding national age-standardized KOA incidence and DALY. Research has shown that Japan had the top prevalence and DALY in 2019 [15], suggesting the changes in the trend of KOA incidence across different countries over the past two years. Therefore, it is necessary to adjust health strategy layouts in a timely manner to address new challenges.

The incidence of KOA is positively linked to age. As the population continues to age, the incidence of KOA is increasing year by year [16,17]. In this study, all age groups were stratified into subgroups every 5 years to more thoroughly explore the relationship between age and KOA. The results revealed that there were no reported cases of KOA in the population under 30 years old. Our findings were consistent with the research results of Li E and others [15]. The global prevalence of KOA significantly increased starting from 35–39 age group, peaking in 80–84 age group. Concurrently, top number of KOA cases was found in 65–69 age group. Data also indicated that KOA was more common in females across all age groups, which aligned with previous studies [18,19] and revealed that KOA had a greater impact on females than on males. This gender difference may be attributed to multiple factors. Research has indicated notable disparities in knee kinematics between genders, with females demonstrating a greater range of knee valgus rotation compared to males. This physiological characteristic makes females more susceptible to knee injuries [20]. Additionally, the incidence of anterior cruciate ligament injuries in adolescent and adult females is 2–8 times higher than that in males during cutting, jumping, and rotating exercises [21].

Globally, the peak incidence of KOA occurred in 55–59 age group, while the top number of cases in both sexes was found in 50–54 age group. Overall KOA DALY rate was highest in 75–79 age group, whereas the actual number of DALYs reached its peak in 55–59 age group. Women exhibited higher numbers of DALYs than men in all age groups. Notably, the overall DALY rate of KOA in 75–79 age group declined after reaching its peak. This result may stems from survivor bias, leading to the conclusion that the disease burden of KOA patients over 80 years old may not necessarily be reduced. In addition, we noted a significant increase in the prevalence of KOA among 0–34, 35–39, and 40–44 age groups, suggesting a trend of KOA developing at younger ages. Obesity and a history of joint injuries are important influencing factors for KOA [22]. Ilana N Ackerman et al. [23] found that overweight and obesity promote OA advancement through both biomechanical factors (increased joint loading) and inflammatory processes. The increasing prevalence of obesity and sports injuries among young people may provide the most plausible explanation for the rise in KOA incidence in younger populations.

The decomposition of KOA DALYs shows that globally, the burden of KOA has increased substantially, with population growth being the main driver. The present study further stratifies the SDI countries into quintiles and shows that the role of aging is more pronounced in high SDI regions, and conversely, in low SDI regions, population growth is the main factor. From 1990 to 2021, the connection between age-standardized DALY rates and SDI was not linear. As SDI elevated, DALY rate initially rose, peaking at an SDI of approximately 0.6, then decreased to 0.7, and subsequently increased again. The burden of KOA increased alongside socio-economic development, with countries and regions like South Korea, Singapore, and Brunei experiencing a much higher burden than expected.

The conventional perception is that countries with high SDI levels are able to be equipped with better healthcare systems, which may reduce the burden of disease. However, this study showed through cross-national health inequality analysis that high SDI regions or countries bear a disproportionate burden of KOA, while morbidity is more significant in regions with higher SDI. This is consistent with the study by Safiri et al [24]. Surveys of countries with high SDI showed that medical expenses for OA accounted for 1% to 2.5% of GDP, with KOA comprising about 85% of this burden [25]. For instance, in the U.S., estimated total lifetime cost linked to opioid medications for KOA patients was around $1.4 billion [26]. The SDI serves as an economic indicator that reflects the overall level of development within a country [27]. The high disease burden of KOA in high SDI nations may be closely correlated with factors such as higher levels of economic development, more comprehensive social welfare and security systems, and improved capabilities for timely health diagnosis and treatment. In addition, the aging population and higher medical standards have also facilitated timely diagnosis and treatment of KOA, thereby enhancing quality of life and increasing life expectancy [6]. A cross-sectional study involving 513 railway workers in Malaysia revealed that 53.6% of participants had limited knowledge about KOA [28]. Moreover, in countries with low SDI, medical care was generally inadequate, and there was a lack of facilities for early diagnosis, which led to many patients with KOA being unable to receive timely treatment [29]. These observations indicated a significant gap in the levels of prevention and awareness of KOA among countries with different SDI levels.These observations indicated a significant gap in the levels of prevention and awareness of KOA among countries with different SDI levels. This disparity highlights the need for better integration of KOA management in primary care and undergraduate medical education, especially in low-and middle-income countries. Improving healthcare professionals’ knowledge about early detection and management of KOA could significantly reduce the disease burden in these regions. Furthermore, strengthening health systems to address chronic conditions like KOA should be a priority in global health policy initiatives aimed at reducing health inequalities.

Research indicated that most KOA patients in high SDI countries had mild cases, while in low SDI countries, most KOA patients experienced moderate to severe forms of the disease [30]. Differences in the level of health care between high SDI and low SDI countries may explain this phenomenon. To mitigate differences in cross-country inequalities, targeted policies should be developed to rationalize the allocation of resources. On the one hand, countries with a high SDI need to focus not only on new drug development and management measures but also on coping with the growing aging population. Meanwhile, the major challenges for KOA diseases in countries with lower SDIs are population growth and lack of healthcare resources. Notably, the results show that inequalities between high and low SDI regions are widening, suggesting that as societies develop and age, more attention and resources should be devoted to the prevention, management, and treatment of KOA.

On a global scale, high BMI served as leading contributor to DALY due to the impact of KOA, accounting for 33.5%. Research has indicated that a 5 kg/m2 elevation in BMI is linked to a 36% elevation in the risk of developing KOA [31,32]. Additionally, previous study [33] has found that BMI can affect walking speed and gait biomechanics, which are important factors influencing KOA. Therefore, weight management is considered a core treatment option for KOA, as it can effectively reduce the incidence of the disease [34,35]. This study predicts that the global burden of KOA will increase significantly by 2035. The increase in incidence and prevalence of KOA is more significant in India compared to China. Notably, while the age-standardized incidence rate (ASR) in China is projected to decrease slightly from 853.4 to 813.9 per 100,000 people, the ASR is projected to increase globally and in India. Combined with previous decomposition analyses, it is suggested that population growth and aging will be important drivers of the increased burden, particularly in India and globally. In China, on the other hand, demographic changes may play a more prominent role than epidemiological factors in the projected increase in the burden of knee osteoarthritis. Therefore, in the future, China should fully consider demographic changes as a factor, with special attention to the aging population and rational resource allocation.

## Advantages and limitations

The strengths stem from being the most recent analysis of global KOA epidemiology to date. It provides the latest and most thorough estimates of KOA levels and trends, as well as its hazard factors, at global, regional, and national levels between 1990 and 2021, including 204 countries, some of which have not been previously assessed. Health inequality analyses were performed using slope index of inequality (SII) and concentration index (CI). Decomposition analysis was conducted to understand the contributions of population growth, aging, and epidemiological changes to the increasing burden. Future projections were made for global, Chinese, and Indian trends to 2035.However, there are some limitations in this work. Firstly, although the GBD database covers 204 countries and regions, the data for certain specific areas may not be sufficiently detailed or accurate, potentially affecting the overall understanding of global health issues. The GBD methodology relies on varying data collection practices across countries, with potentially inconsistent diagnostic criteria and surveillance systems. These variations may introduce bias, particularly in regions with less robust healthcare infrastructure. While the GBD study employs statistical methods to address these limitations, caution should be exercised when interpreting results from countries with limited primary data sources. Secondly, the study is highly dependent on national health information systems and hospital records, potentially leading to an underestimation of true incidence of KOA. Finally, the database omits important information about risk factors like dietary habits and past joint injuries. This will result in an underestimation of the true numbers in the study cohort.

## Conclusions

KOA represents a great social health challenge, incurring heavy healthcare and economic costs. The global rise in prevalence, morbidity, and DALY rates, coupled with an aging population and social advancement, suggests that KOA continues to impose a significant economic burden and will likely become an even larger public health concern in the future. This study highlighted that KOA burden differed across nations, by gender, age, and income level. From 1990 to 2021, the change in prevalence among males was more pronounced than among females, especially in Africa, America, Australasia, and Western Europe. This finding underscored the need for greater attention to be paid to the male population in future health strategies. Analyses of health inequalities show growing national disparities between different SDI levels. The projected increase highlights the need for targeted interventions, especially in rapidly growing populations. Preventive measures should focus on reducing high body mass index, implementing gender-specific treatments and addressing regional disparities. This study can enhance public awareness and the understanding of national policymakers regarding KOA. This knowledge can guide policymakers in designing effective control measures and services to meet the growing medical demand for KOA and its comorbidities, thereby reducing the burden of future diseases.Addressing the growing burden of KOA requires comprehensive multidisciplinary rehabilitation approaches and early intervention strategies, particularly in resource-limited settings. Effective rehabilitation programs should emphasize patient education, exercise therapy, weight management, and pain control through both pharmacological and non-pharmacological means [36]. Patient empowerment through self-management education is especially crucial, as it can improve adherence to treatment regimens and reduce healthcare utilization. A structured approach to KOA management that incorporates both preventive measures and rehabilitation strategies can significantly reduce disease progression and improve quality of life for affected individuals.

## Supporting information

S1 File S1 Fig. The age-standardised point Prevalence of knee Osteoarthritis disease in 2021 for the 21 Global Burden of Disease regions, by sex. S2 Fig. The age-standardised point Incidence of knee Osteoarthritis disease in 2021 for the 21 Global Burden of Disease regions, by sex. S3 Fig. The age-standardised point DALYs of knee Osteoarthritis disease in 2021 for the 21 Global Burden of Disease regions, by sex. S4 Fig.The percentage change in the age-standardised point Prevalence of knee Osteoarthritis disease from 1990 to 2021 for the 21 Global Burden of Disease regions,by sex. S5 Fig. The percentage change in the age-standardised point Incidence of knee Osteoarthritis disease from 1990 to 2021 for the 21 Global Burden of Disease regions,by sex. S6 Fig. The percentage change in the age-standardised point DALYs of knee Osteoarthritis disease from 1990 to 2021 for the 21 Global Burden of Disease regions,by sex. S7 Fig. Age-standardised DALYs rate of knee Osteoarthritis disease per 100,000 population in 2021, by country. (Note: The maps were created using the R language and open source software packages and data.) S8 Fig. Global number of Incidence and Incidence rate of knee Osteoarthritis disease per 100,000 population, by age and sex, in 2021; Dotted and dashed lines indicate 95% upper and lower uncertainty intervals, respectively. S9 Fig. Global number of DALYs and DALYs rate of knee Osteoarthritis disease per 100,000 population, by age and sex, in 2021; Dotted and dashed lines indicate 95% upper and lower uncertainty intervals, respectively. S10 Fig. Age-standardised DALY rates of knee Osteoarthritis disease for 204 countries and territories, by SDI, in 2021; Expected values based on the Socio-demographic Index and disease rates in all locations are shown as the black line. Each point shows the observed agestandardised DALY rate for each country in 2021. S11 Fig. Percentage of DALYs due to knee Osteoarthritis disease attributable to risk factors among males for 21 GBD regions in 2021. DALY = disability adjusted life years. S12 Fig. Percentage of DALYs due to knee Osteoarthritis disease attributable to risk factors among Females for 21 GBD regions in 2021. DALY = disability adjusted life years. S13 Fig. Percentage of DALYs due to knee Osteoarthritis disease attributable to risk factor, by age, in 2021. DALY = disability adjusted life years. S14 Fig. Percentage of DALYs due to knee Osteoarthritis disease attributable to risk factor among males, by age, in 2021. DALY = disability adjusted life years. S15 Fig. Percentage of DALYs due to knee Osteoarthritis disease attributable to risk factor among Females, by age, in 2021. DALY = disability adjusted life years. S1 Table. Prevalent cases of knee Osteoarthritis disease in 1990 and 2021 the percentage change in the age-standardised rates (ASRs) per 100,000, by location. S2 Table. Incidence cases of knee Osteoarthritis disease in 1990 and 2021 the percentage change in the age-standardised rates (ASRs) per 100,000, by location. S3 Table. DALYs cases of knee Osteoarthritis disease in 1990 and 2021 the percentage change in the age-standardised rates (ASRs) per 100,000, by location.
(PDF)
