# Supplementary material for: Global burden of knee osteoarthritis from 1990 to 2021: Trends, inequalities, and projections to 2035
Source: PLoS One. 2025 Jun 11;20(6):e0320115. doi: 10.1371/journal.pone.0320115 (PMC12157667; doi:10.1371/journal.pone.0320115)
Supplement: S1 File — S1 Fig. The age-standardised point Prevalence of knee Osteoarthritis disease in 2021 for the 21 Global Burden of Disease regions, by sex. S2 Fig. The age-standardised point Incidence of knee Osteoarthritis disease in 2021 for the 21 Global Burden of Disease regions, by sex. S3 Fig. The age-standardised point DALYs of knee Osteoarthritis disease in 2021 for the 21 Global Burden of Disease regions, by sex. S4 Fig.The percentage change in the age-standardised point Prevalence of knee Osteoarthritis disease from 1990 to 2021 for the 21 Global Burden of Disease regions,by sex. S5 Fig. The percentage change in the age-standardised point Incidence of knee Osteoarthritis disease from 1990 to 2021 for the 21 Global Burden of Disease regions,by sex. S6 Fig. The percentage change in the age-standardised point DALYs of knee Osteoarthritis disease from 1990 to 2021 for the 21 Global Burden of Disease regions,by sex. S7 Fig. Age-standardised DALYs rate of knee Osteoarthritis disease per 100,000 population in 2021, by country. (Note: The maps were created using the R language and open source software packages and data.) S8 Fig. Global number of Incidence and Incidence rate of knee Osteoarthritis disease per 100,000 population, by age and sex, in 2021; Dotted and dashed lines indicate 95% upper and lower uncertainty intervals, respectively. S9 Fig. Global number of DALYs and DALYs rate of knee Osteoarthritis disease per 100,000 population, by age and sex, in 2021; Dotted and dashed lines indicate 95% upper and lower uncertainty intervals, respectively. S10 Fig. Age-standardised DALY rates of knee Osteoarthritis disease for 204 countries and territories, by SDI, in 2021; Expected values based on the Socio-demographic Index and disease rates in all locations are shown as the black line. Each point shows the observed agestandardised DALY rate for each country in 2021. S11 Fig. Percentage of DALYs due to knee Osteoarthritis disease attributable to risk factors among males for 21 GBD re [file pone.0320115.s001.pdf]

Global Burden of Knee Osteoarthritis from 1990 to 2021: Trends, Inequalities, and Projections to 2035

Junjie Chen [1¶], Xianshuai Chen [2¶], Tianshu Wang [1], Ming Li [3], Huanhuan Dai [4],

Shuangshuang Shang [3], Lili Cheng [1], Zhongfu Tang [1], Sidi Liu[3], Chuanbing Huang [3*].

S1 File:

Figure S1: The age-standardised point Prevalence of knee Osteoarthritis disease in 2021 for the 21 Global Burden of Disease regions, by sex.

Figure S2: The age-standardised point Incidence of knee Osteoarthritis disease in 2021 for the 21 Global Burden of Disease regions, by sex.

Figure S3: The age-standardised point DALYs of knee Osteoarthritis disease in 2021 for the 21 Global Burden of Disease regions, by sex.

Figure S4: The percentage change in the age-standardised point Prevalence of knee Osteoarthritis disease from 1990 to 2021 for the 21 Global Burden of Disease regions,by sex.

Figure S5: The percentage change in the age-standardised point Incidence of knee Osteoarthritis disease from 1990 to 2021 for the 21 Global Burden of Disease regions,by sex.

Figure S6: The percentage change in the age-standardised point DALYs of knee Osteoarthritis disease from 1990 to 2021 for the 21 Global Burden of Disease regions,by sex.

Figure S7: Age-standardised DALYs rate of knee Osteoarthritis disease per 100,000 population in 2021, by country. (Note: The maps were created using the R language and open source software packages and data)

Figure S8: Global number of Incidence and Incidence rate of knee Osteoarthritis disease per 100,000 population, by age and sex, in 2021; Dotted and dashed lines indicate 95% upper and lower uncertainty intervals, respectively.

Figure S9: Global number of DALYs and DALYs rate of knee Osteoarthritis disease

per 100,000 population, by age and sex, in 2021; Dotted and dashed lines indicate 95% upper and lower uncertainty intervals, respectively.

Figure S10: Age-standardised DALY rates of knee Osteoarthritis disease for 204 countries and territories, by SDI, in 2021; Expected values based on the Socio-demographic Index and disease rates in all locations are shown as the black line. Each point shows the observed agestandardised
DALY rate for each country in 2021.

Figure S11: Percentage of DALYs due to knee Osteoarthritis disease attributable to risk factors among males for 21 GBD regions in 2021. DALY=disability adjusted life years
Figure S12: Percentage of DALYs due to knee Osteoarthritis disease attributable to risk factors among Females for 21 GBD regions in 2021. DALY=disability adjusted life years

Figure S13: Percentage of DALYs due to knee Osteoarthritis disease attributable to risk factor, by age, in 2021. DALY=disability adjusted life years

Figure S14: Percentage of DALYs due to knee Osteoarthritis disease attributable to risk factor among males, by age, in 2021. DALY=disability adjusted life years

Figure S15: Percentage of DALYs due to knee Osteoarthritis disease attributable to risk factor among Females, by age, in 2021. DALY=disability adjusted life years

Table S1: Prevalent cases of knee Osteoarthritis disease in 1990 and 2021 the percentage change in the age-standardised rates (ASRs) per 100,000, by location.

Table S2 Incidence cases of knee Osteoarthritis disease in 1990 and 2021 the percentage change in the age-standardised rates (ASRs) per 100,000, by location.

Table S3 DALYs cases of knee Osteoarthritis disease in 1990 and 2021 the percentage change in the age-standardised rates (ASRs) per 100,000, by location.

Figure S1: The age-standardised point Prevalence of knee Osteoarthritis disease in 2021 for the 21 Global Burden of Disease regions, by sex.

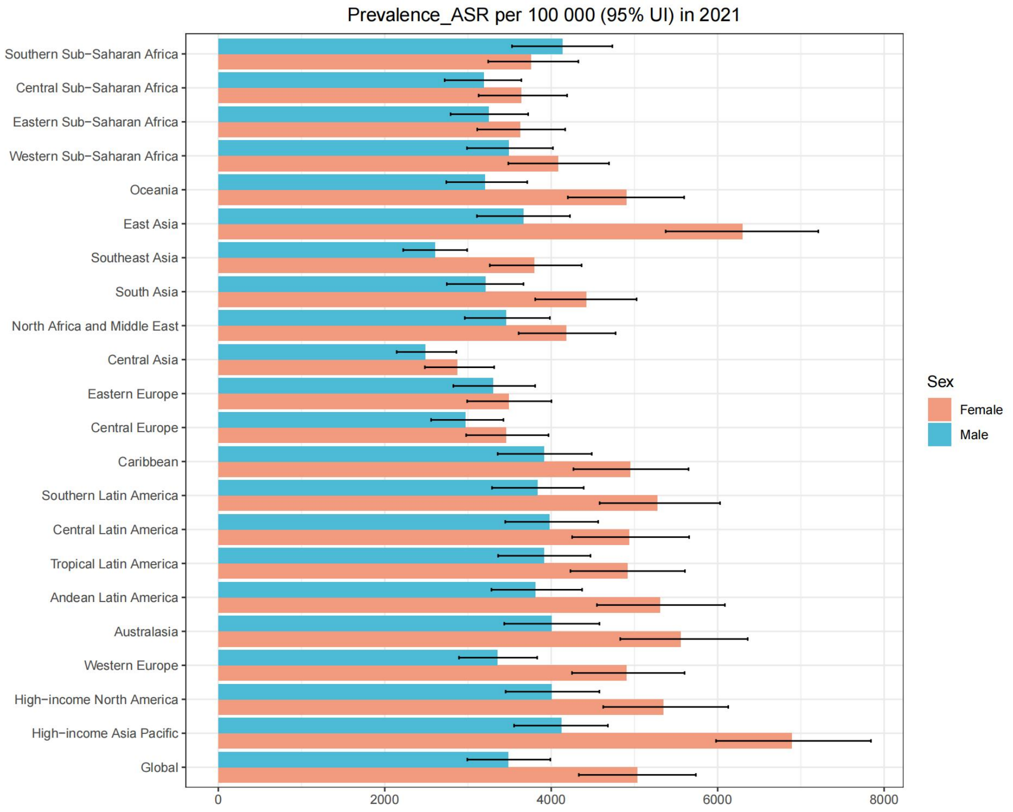

Figure S2: The age-standardised point Incidence of knee Osteoarthritis disease in 2021 for the 21 Global Burden of Disease regions, by sex.

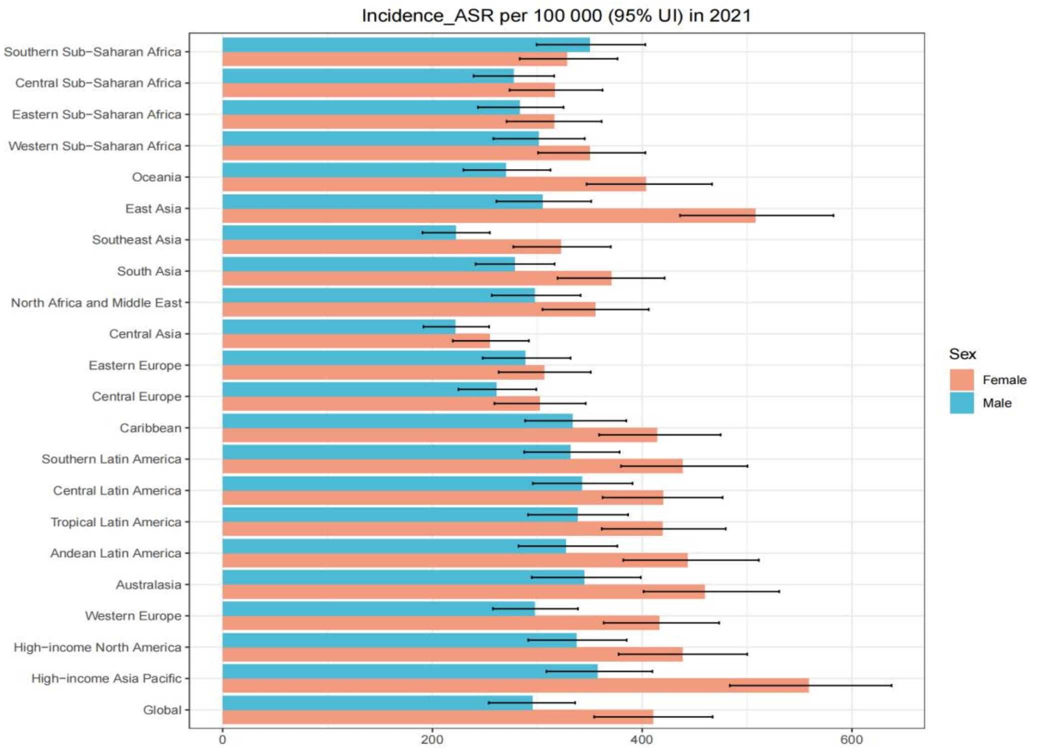

Figure S3: The age-standardised point DALYs of knee Osteoarthritis disease in 2021 for the 21 Global Burden of Disease regions, by sex.

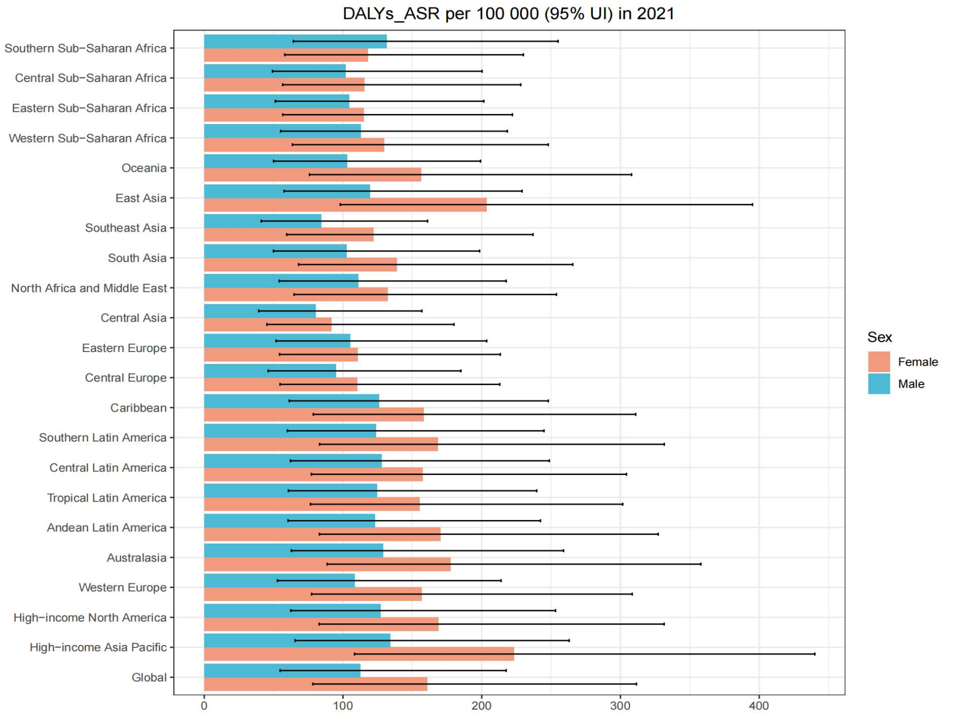

Figure S4: The percentage change in the age-standardised point Prevalence of knee Osteoarthritis disease from 1990 to 2021 for the 21 Global Burden of Disease regions,by sex.

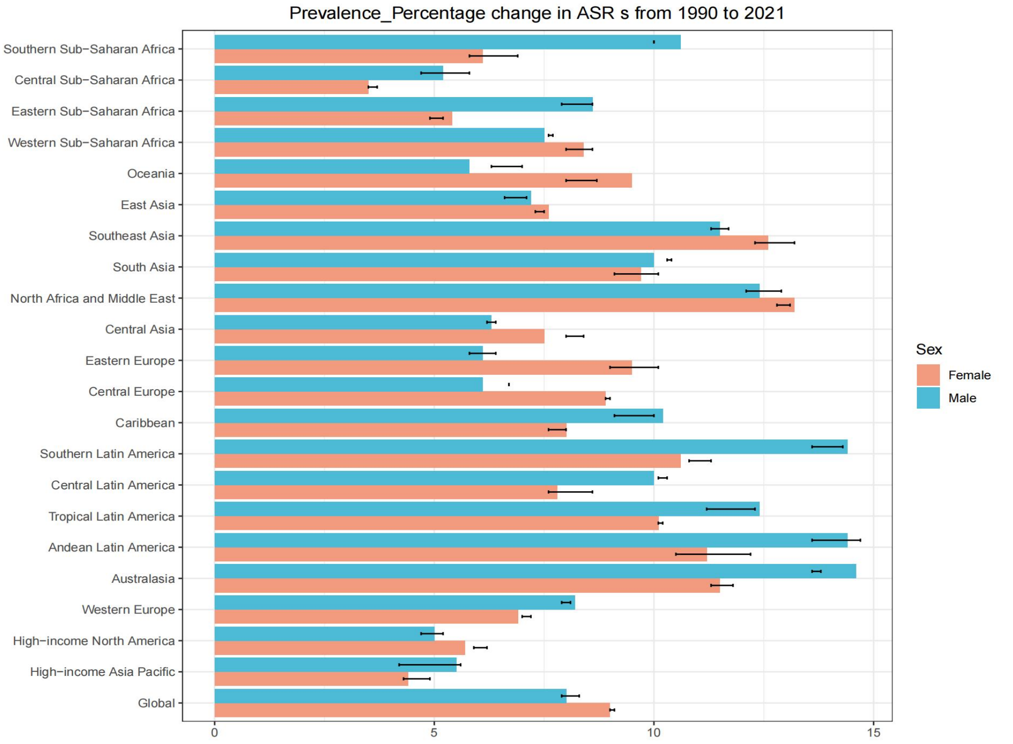

Figure S5: The percentage change in the age-standardised point Incidence of knee Osteoarthritis disease from 1990 to 2021 for the 21 Global Burden of Disease regions,by sex.

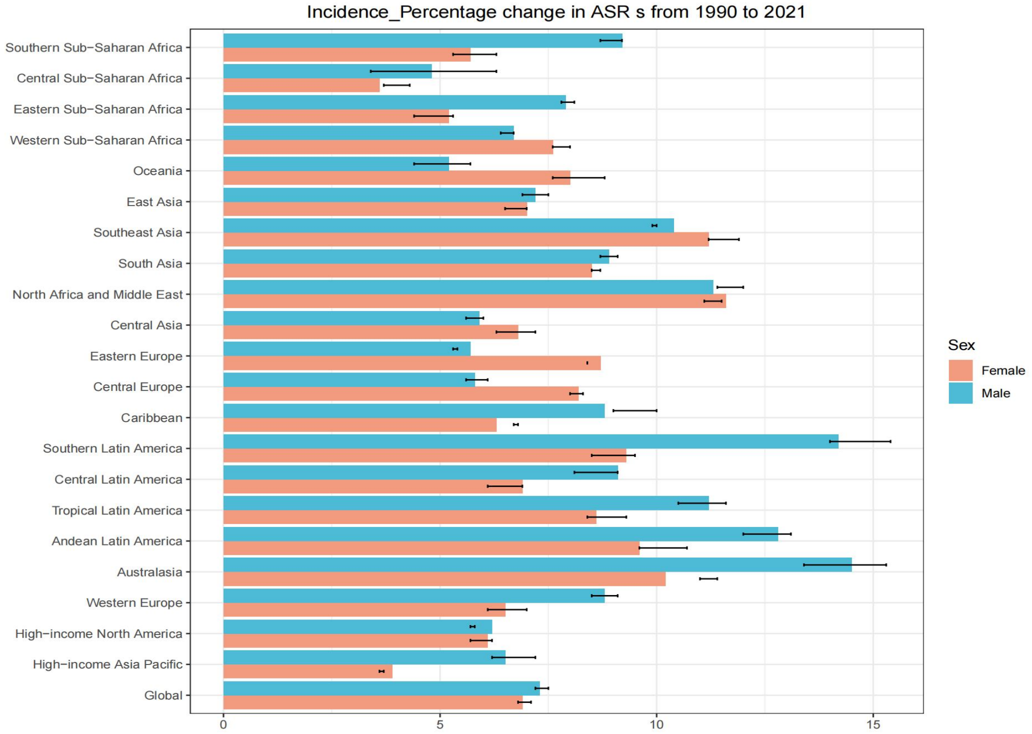

Figure S6: The percentage change in the age-standardised point DALYs of knee Osteoarthritis disease from 1990 to 2021 for the 21 Global Burden of Disease regions,by sex.

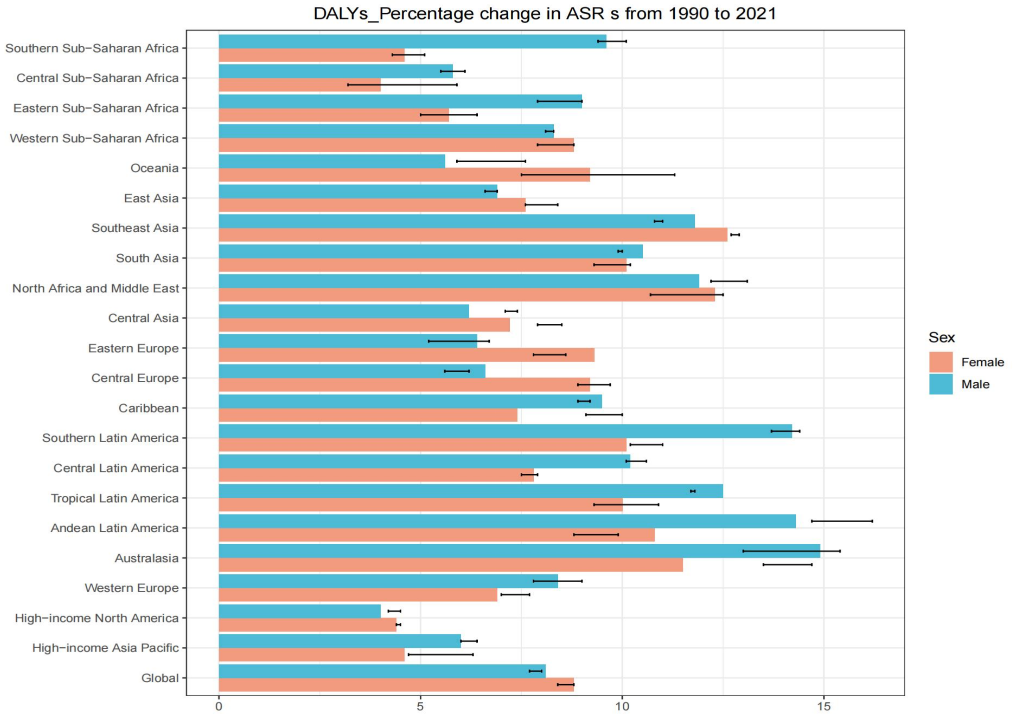

Figure S7: Age-standardised DALYs rate of knee Osteoarthritis disease per 100,000 population in 2021, by country.

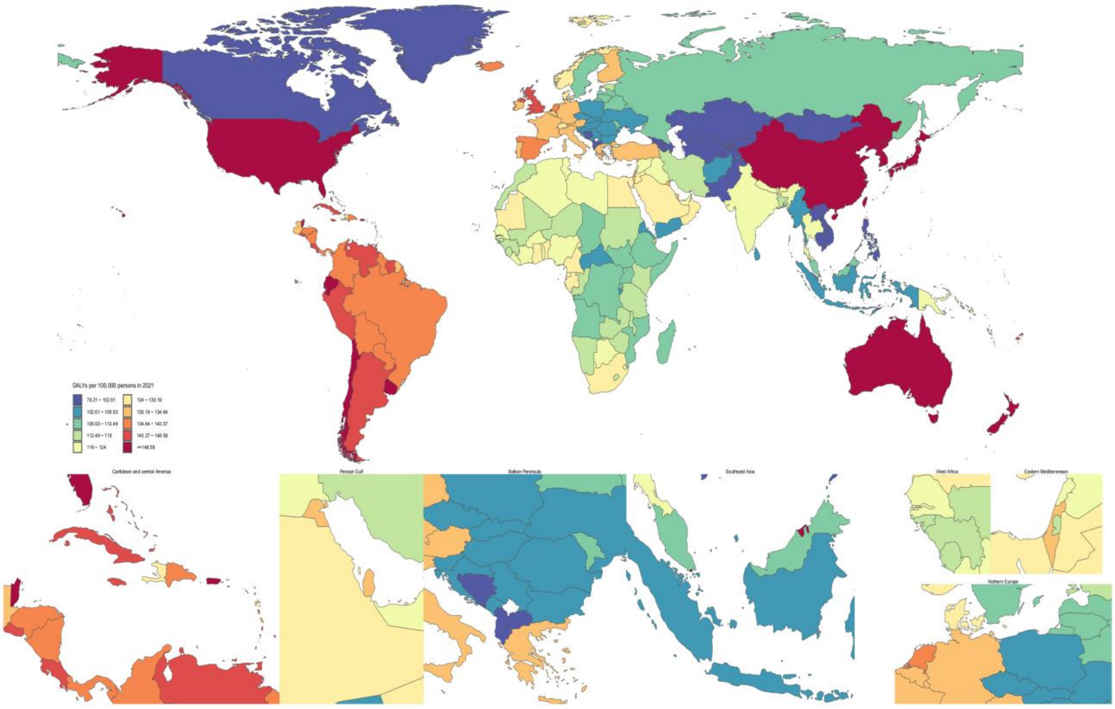

Figure S8: Global number of Incidence and Incidence rate of knee Osteoarthritis disease per 100,000 population, by age and sex, in 2021; Dotted and dashed lines indicate 95% upper and lower uncertainty intervals, respectively.

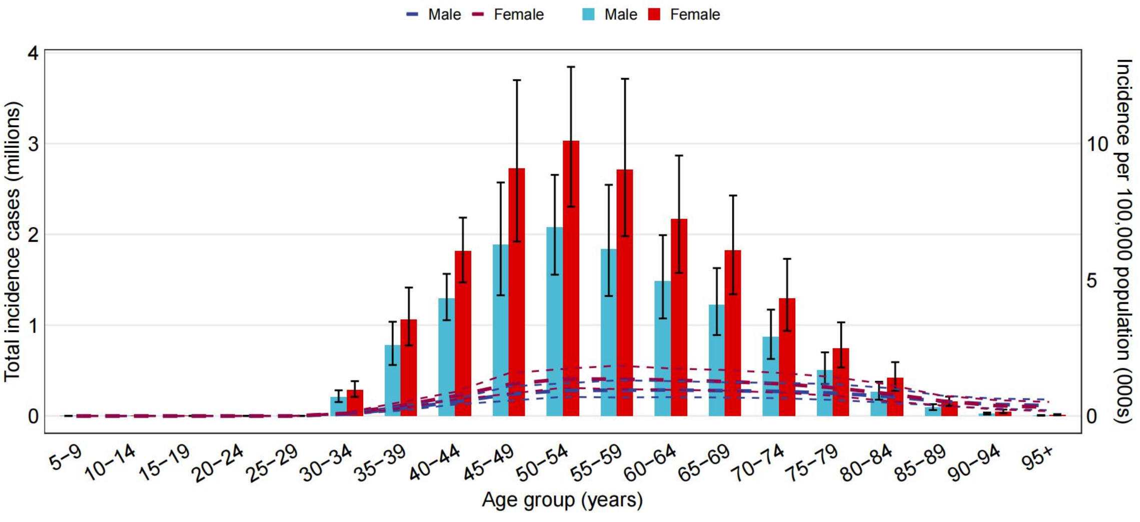

Figure S9: Global number of DALYs and DALYs rate of knee Osteoarthritis disease per 100,000 population, by age and sex, in 2021; Dotted and dashed lines indicate 95% upper and lower uncertainty intervals, respectively.

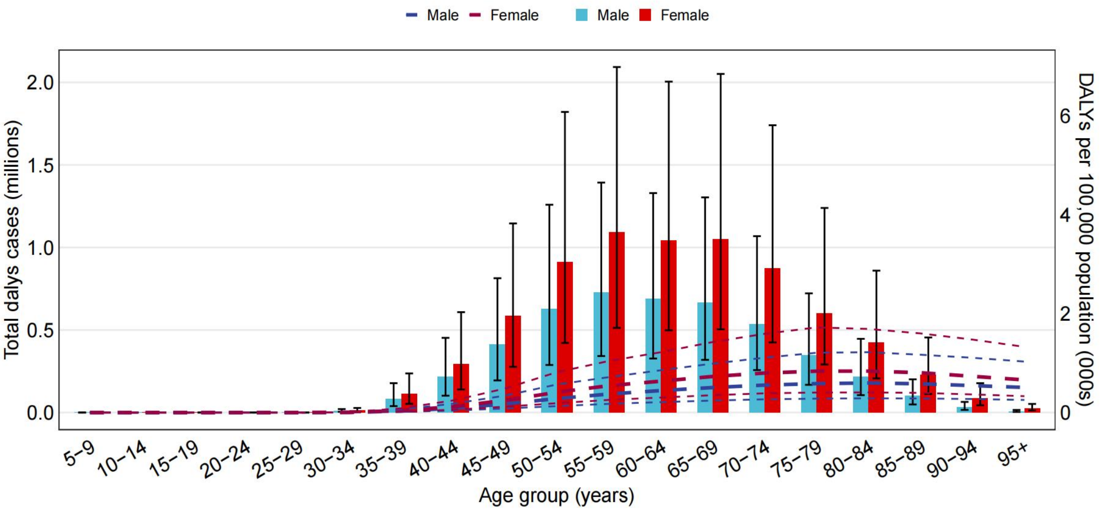

Figure S10: Age-standardised DALY rates of knee Osteoarthritis disease for 204 countries and territories, by SDI, in 2021;（Expected values based on the Socio-demographic Index and disease rates in all locations are shown as the black line. Each point shows the observed agestandardised DALY rate for each country in 2021.）

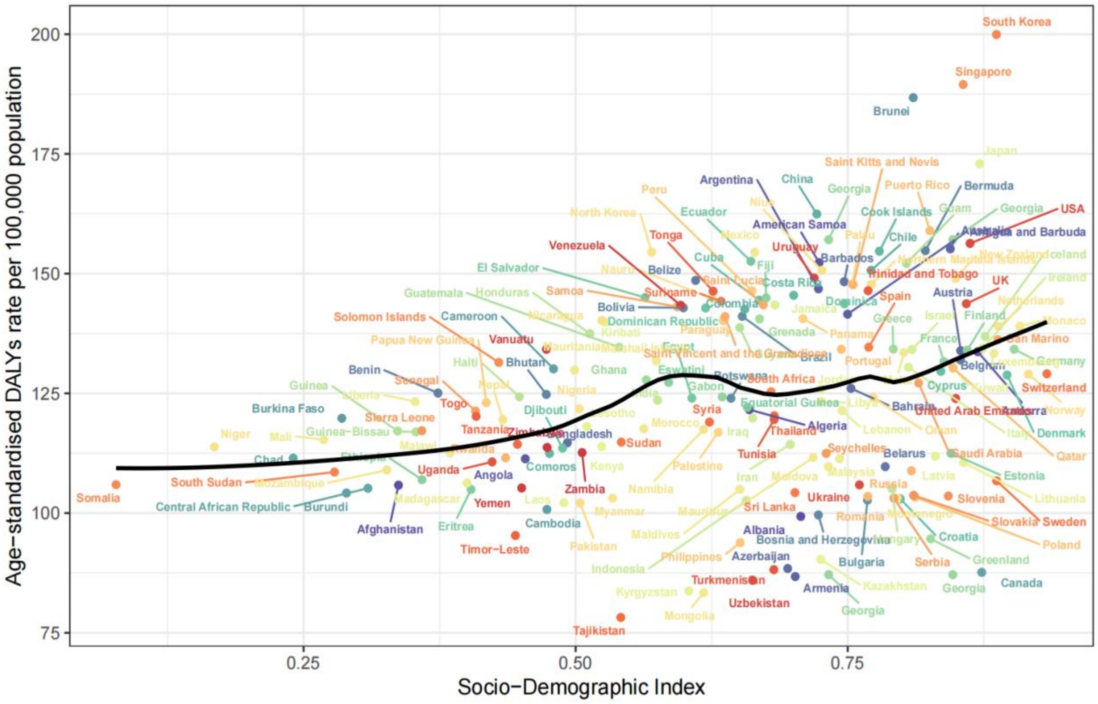

Figure S11: Percentage of DALYs due to knee Osteoarthritis disease attributable to risk factors among males for 21 GBD regions in 2021. DALY=disability adjusted life years

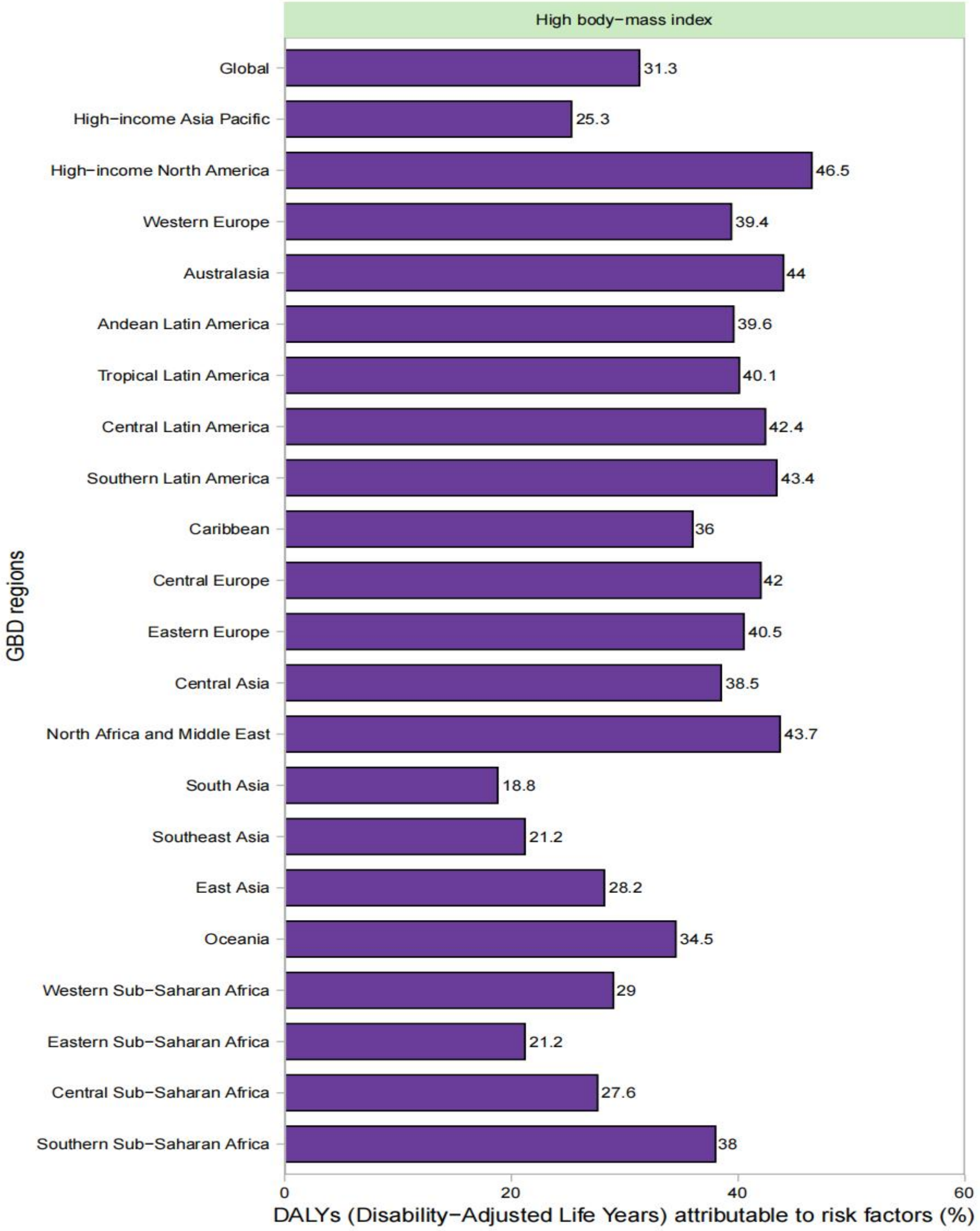

Figure S12: Percentage of DALYs due to knee Osteoarthritis disease attributable to risk factors among Females for 21 GBD regions in 2021. DALY=disability adjusted life years

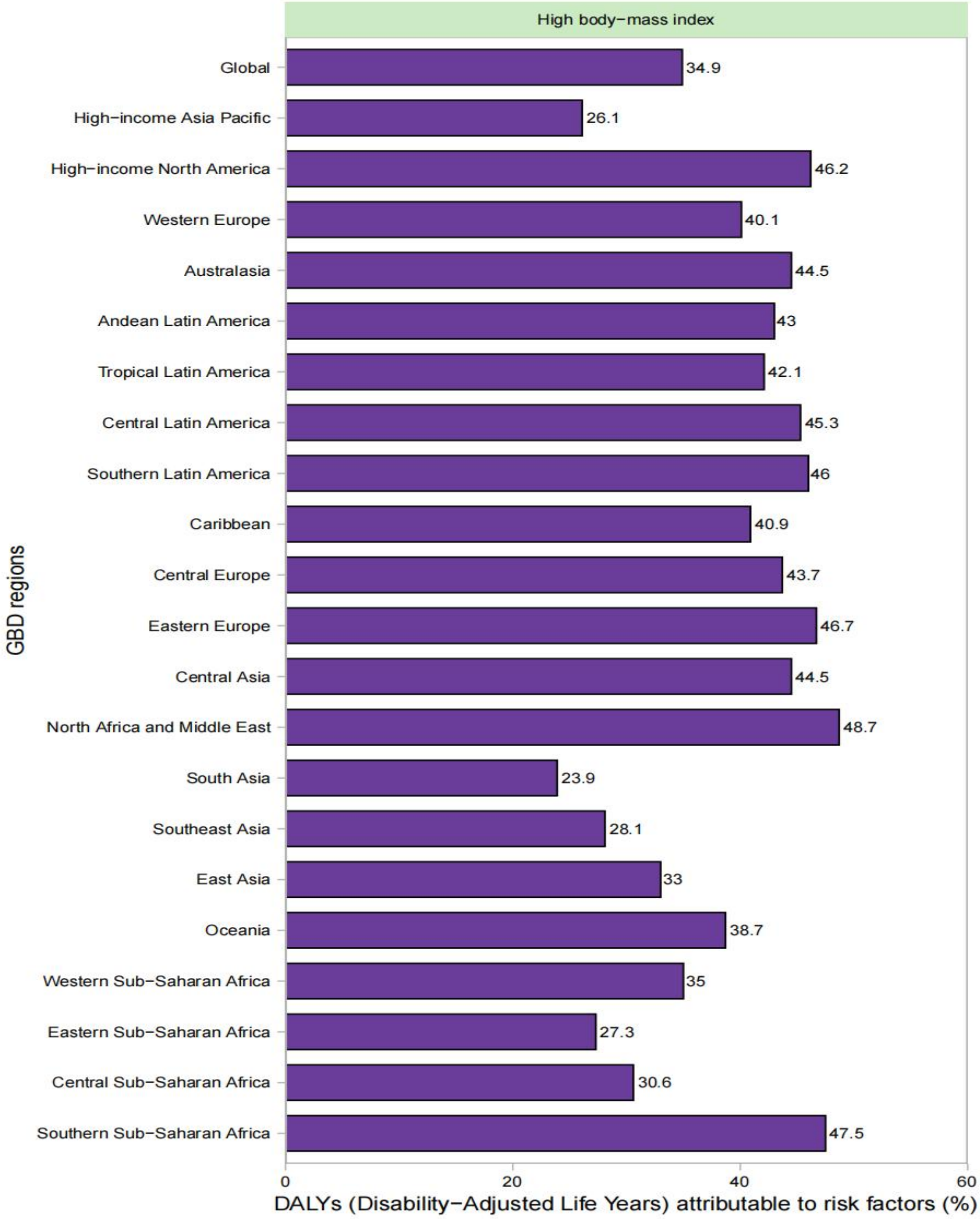

Figure S13: Percentage of DALYs due to knee Osteoarthritis disease attributable to risk factor, by age, in 2021. DALY=disability adjusted life years

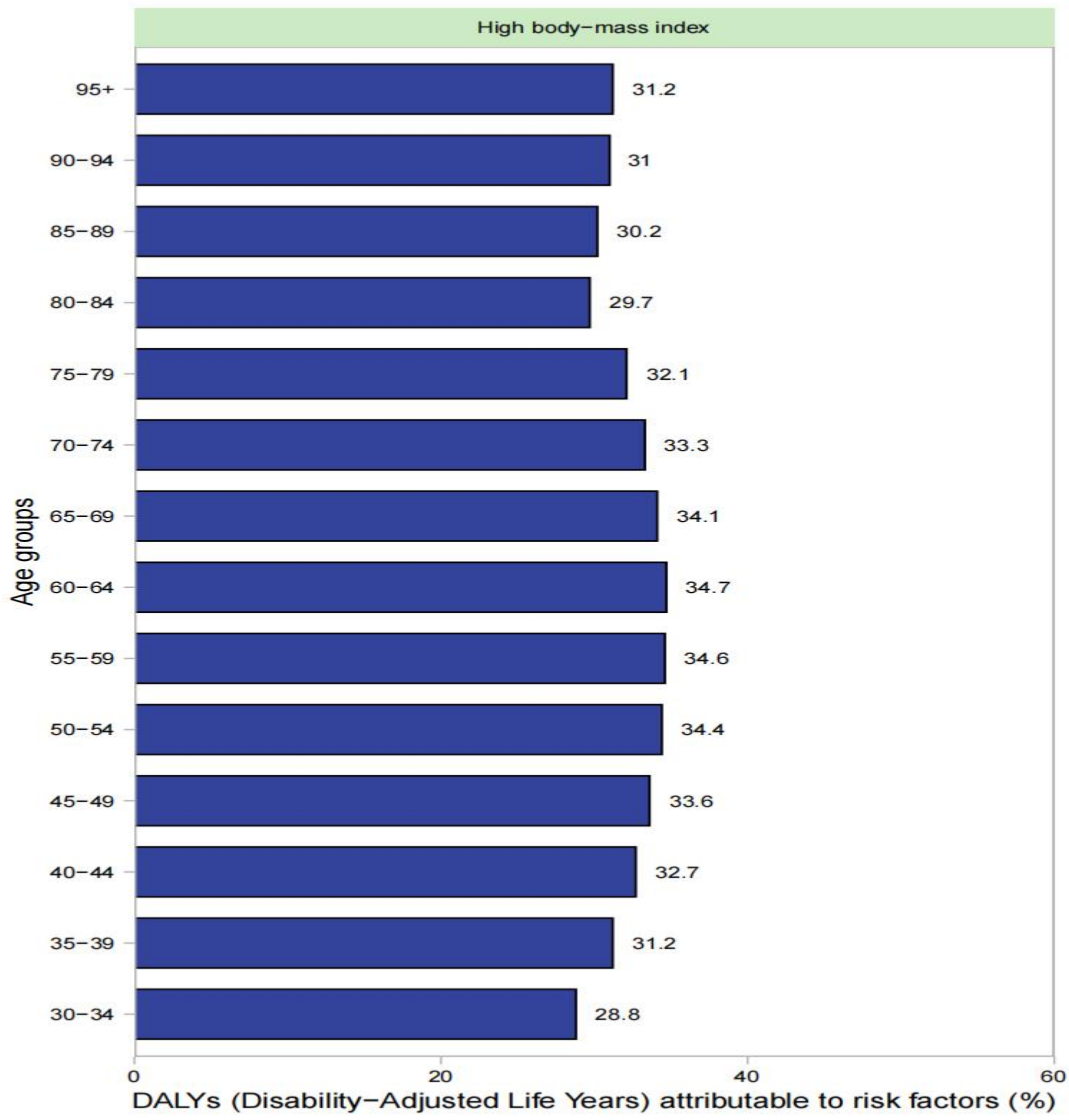

Figure S14: Percentage of DALYs due to knee Osteoarthritis disease attributable to risk factor among males, by age, in 2021. DALY=disability adjusted life years

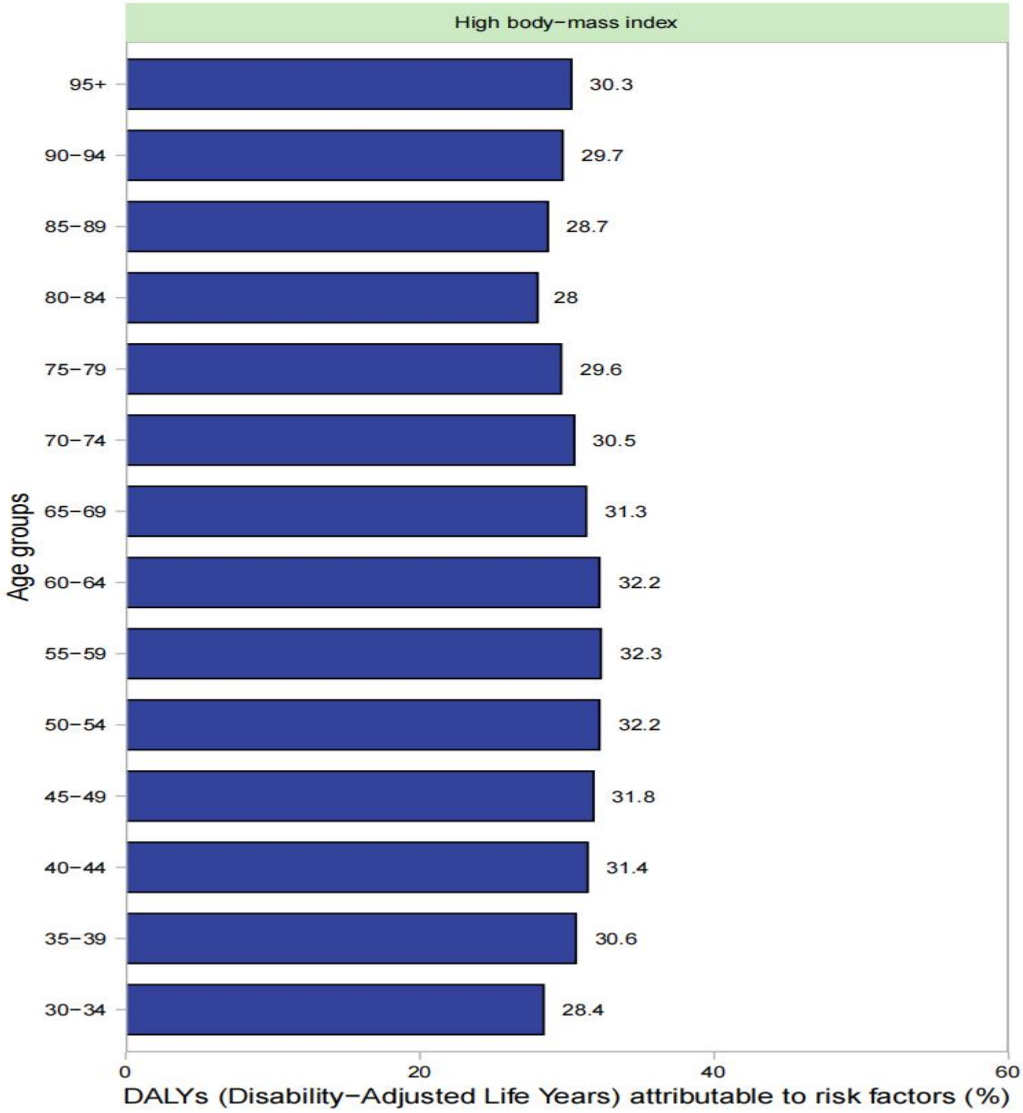

Figure S15: Percentage of DALYs due to knee Osteoarthritis disease attributable to risk factor among Females, by age, in 2021. DALY=disability adjusted life years

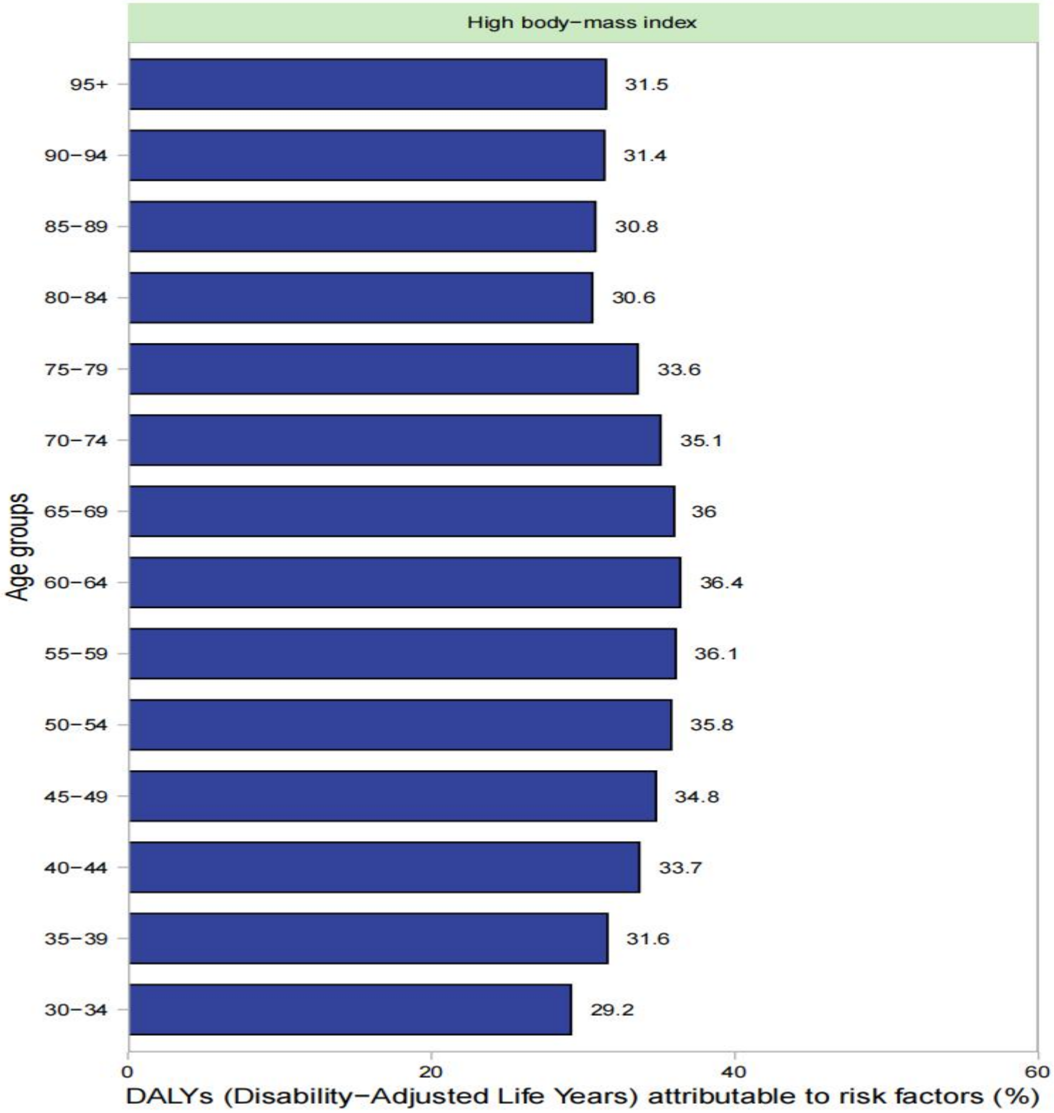

Table S1: Prevalent cases of knee Osteoarthritis disease in 1990 and 2021 the percentage change in the age-standardised rates (ASRs) per 100,000, by location.

	1990		2021		Percentage change in the ASRs per 100,000
	No (95% UI)	ASRs per 100,000 (95% UI)	No (95% UI)	ASRs per 100,000 (95% UI)	
**Global**	159798909 (137277437,182882554)	3964.75 (3411.86,4536.4)	374738744 (321858982,428353220)	4294.27 (3695.04,4910.76)	8.31 (8.25,8.3)
**High-income North America**	15143603 (13138164,17316618)	4500.08 (3894.98,5121.91)	29008914 (25038463,33373205)	4709.03 (4074.67,5394.69)	4.64 (4.61,5.33)
Canada	763567 (665048,872710)	2389.67 (2079.91,2727.73)	1817661 (1572491,2082024)	2722.94 (2357.99,3111.62)	13.95 (13.37,14.07)
Greenland	965 (825,1111)	2645.9 (2294.84,3060.27)	2146 (1818,2486)	2947.19 (2534.73,3400.08)	11.39 (10.45,11.1)
United States of America	14378724 (12464702,16442866)	4724.28 (4082.75,5379.27)	27188653 (23424909,31332329)	4949.15 (4275.55,5677.95)	4.76 (4.72,5.55)
**Australasia**	993266 (867688,1128276)	4288.08 (3746.3,4863.21)	2389034 (2066114,2736476)	4808.67 (4147.42,5488.06)	12.14 (10.71,12.85)
Australia	831099 (721746,942383)	4301.83 (3760,4860.33)	2016533 (1745326,2312719)	4838.82 (4179.84,5526.62)	12.48 (11.17,13.71)
New Zealand	162168 (140535,186972)	4217.34 (3656.32,4871.82)	372501 (320467,427080)	4650.96 (3996.64,5313.53)	10.28 (9.07,9.31)
**High-income Asia Pacific**	11135071 (9603769,12692418)	5416.8 (4683,6163.06)	22540484 (19570221,25550915)	5573.73 (4841.29,6334.33)	2.9 (2.78,3.38)
Brunei Darussalam	5804 (5033,6577)	5364.25 (4677.82,6069.33)	22212 (19106,25249)	5824.76 (5024.72,6623.85)	8.58 (7.42,9.14)
Japan	9097297 (7864578,10385046)	5281.71 (4574.98,6014.74)	16173245 (14066813,18316284)	5331.09 (4598.71,6051.31)	0.93 (0.52,0.61)
Singapore	128188 (109955,146159)	5559.36 (4791.6,6298.06)	509663 (438044,580344)	5810.72 (5004.32,6619.23)	4.52 (4.44,5.1)
Republic of Korea	1903781 (1628477,2205126)	6160.49 (5327.87,7052.88)	5835363 (5072731,6672902)	6201.62 (5389.51,7093.43)	0.67 (0.57,1.16)
**Western Europe**	21982300 (19056229,25147170)	3934.19 (3410.31,4494.01)	35172347 (30617674,40161660)	4169.47 (3611.88,4769.26)	5.98 (5.91,6.12)
Andorra	2245 (1936,2594)	3844.45 (3315.52,4443.59)	6349 (5503,7248)	4155.42 (3604.99,4740.56)	8.09 (6.68,8.73)
Austria	445917 (388835,511161)	3930.56 (3422.97,4498.89)	695927 (603795,795871)	4158.81 (3598.54,4757.78)	5.81 (5.13,5.75)
Belgium	574390 (494091,661544)	3872.12 (3351.77,4458.21)	873012 (765528,1006978)	4115.76 (3575.6,4732.95)	6.29 (6.16,6.68)
Cyprus	30822 (26705,35058)	3718.02 (3228.54,4234.67)	81855 (70941,94417)	4024.52 (3478.54,4627.78)	8.24 (7.74,9.28)

Denmark	286183 (248975,326949)	3723.45 (3231.38,4262.53)	433950 (376400,501332)	3987.37 (3449.18,4546.17)	7.09 (6.65,6.74)
Finland	276093 (238397,315027)	3956.76 (3413.89,4490.47)	469620 (405078,545172)	4177.96 (3620.74,4828.33)	5.59 (6.06,7.52)
France	3047416 (2644900,3479039)	3843.85 (3331.7,4356.53)	5118522 (4460457,5923102)	4100.74 (3547.57,4689.32)	6.68 (6.48,7.64)
Germany	4897596 (4248140,5602423)	3988.98 (3461.41,4566.2)	7294669 (6310536,8362362)	4185.74 (3634.76,4827.33)	4.93 (5.01,5.72)
Greece	585694 (506602,669450)	3905.01 (3403.26,4454.55)	880644 (757764,1003033)	4167.01 (3580.41,4744.11)	6.71 (5.21,6.5)
Iceland	10900 (9505,12597)	3956.39 (3416.3,4548.45)	23114 (20024,26528)	4229.86 (3666.61,4826.45)	6.91 (6.11,7.33)
Ireland	156088 (134007,179705)	3909.91 (3367.27,4517.75)	317602 (275005,361519)	4171.56 (3618.17,4738.47)	6.69 (4.89,7.45)
Israel	183686 (159563,211016)	3862.31 (3349.75,4427.67)	487869 (423741,557538)	4156.01 (3595.59,4754.83)	7.6 (7.34,7.39)
Italy	3361885 (2925969,3864633)	3875.64 (3373.53,4435.14)	5275983 (4606861,6017508)	4061.26 (3515.73,4618.4)	4.79 (4.13,4.22)
Luxembourg	21172 (18269,24438)	3944.75 (3414.39,4540.97)	42307 (36949,48000)	4136.34 (3612.23,4691.6)	4.86 (3.32,5.79)
Malta	16495 (14314,18708)	3852.25 (3351.51,4359.87)	36704 (32099,42341)	4140.39 (3614.23,4756.12)	7.48 (7.84,9.09)
Monaco	2600 (2256,3030)	4093.75 (3532.49,4757.69)	3741 (3214,4304)	4300.56 (3702.01,4898.03)	5.05 (2.95,4.8)
Netherlands	792773 (690006,903653)	4084.32 (3540.59,4662.41)	1408067 (1216956,1604086)	4311.29 (3722.66,4889.26)	5.56 (4.87,5.14)
Norway	238862 (206682,273108)	3775.32 (3274.65,4328.48)	373658 (324182,430815)	4010.67 (3478.87,4616.94)	6.23 (6.24,6.66)
Portugal	532111 (457985,605045)	3857.32 (3357.03,4379.17)	926560 (803853,1063052)	4178.17 (3628.09,4802.03)	8.32 (8.07,9.66)
San Marino	1344 (1160,1557)	3983.58 (3423.38,4573.39)	2826 (2452,3227)	4224.91 (3624.56,4835.89)	6.06 (5.74,5.88)
Spain	2103321 (1811409,2433974)	3940.09 (3386.92,4551.32)	3736142 (3252518,4271864)	4181.2 (3624.45,4811.83)	6.12 (5.72,7.01)
Sweden	429758 (367199,506534)	3053.54 (2588.45,3582.26)	638763 (534402,753804)	3312.3 (2766.78,3874.95)	8.47 (6.89,8.17)
Switzerland	384283 (330922,438909)	3870.53 (3325.93,4414.23)	666652 (576401,771112)	4026.29 (3474.53,4665.92)	4.02 (4.47,5.7)
United Kingdom	3582601 (3116475,4119177)	4174.32 (3627.99,4778.45)	5346842 (4647728,6099658)	4480.33 (3893.78,5098.98)	7.33 (6.71,7.33)
**Southern Latin America**	1920099 (1659204,2191582)	4128.09 (3574.76,4711.31)	3942908 (3422500,4496778)	4617.51 (4009.6,5266.68)	11.86 (11.79,12.16)
Uruguay	158091 (136165,179919)	4133.63	238369	4646.43	12.41

		(3568.7,4712.42)	(208096,274000)	(4035.17,5333.8)	(13.07,13.19)
Argentina	1329777 (1144882,1523632)	4094.38 (3531.14,4675.36)	2506329 (2162070,2852391)	4569.29 (3940.95,5225.46)	11.6 (11.61,11.77)
Chile	432139 (372442,499516)	4232.2 (3674.19,4889.58)	1197992 (1040854,1370000)	4714.37 (4096.4,5375.03)	11.39 (9.93,11.49)
**Eastern Europe**	8853765 (7574166,10111455)	3154.42 (2713.05,3603.85)	11762775 (10059205,13517483)	3409.25 (2919.69,3923.46)	8.08 (7.62,8.87)
Belarus	413994 (355113,474109)	3185.62 (2738.3,3657.52)	541760 (462469,627494)	3433.67 (2941.39,3964.55)	7.79 (7.42,8.39)
Estonia	66220 (56940,77124)	3234.22 (2784.18,3754.32)	85919 (74036,98759)	3515.13 (2992.55,4030.27)	8.69 (7.35,7.48)
Latvia	116754 (100005,136052)	3262.48 (2798.23,3783.17)	127599 (109617,146829)	3502.9 (3027.96,4013.9)	7.37 (6.1,8.21)
Lithuania	145934 (125952,166505)	3229.52 (2796.17,3674.17)	184271 (158593,213836)	3462.18 (2963.32,4000.86)	7.2 (5.98,8.89)
Republic of Moldova	138357 (117722,159612)	3108.29 (2661.41,3573.16)	201154 (172093,233714)	3426.84 (2939.9,3949.97)	10.25 (10.46,10.55)
Russian Federation	5693406 (4865290,6531356)	3138 (2695.01,3602.6)	8092104 (6934167,9299270)	3428.99 (2949.71,3939.79)	9.27 (9.36,9.45)
Ukraine	2279101 (1944838,2630430)	3180.29 (2718.32,3660.01)	2529968 (2160023,2923027)	3331.62 (2843.68,3849.75)	4.76 (4.61,5.18)
**Central Europe**	4512504 (3866745,5163412)	3005.2 (2586.99,3442.37)	6774519 (5844666,7842619)	3230.75 (2788.51,3718.9)	7.51 (7.79,8.03)
Albania	60340 (51813,68996)	2836.81 (2441.91,3228.51)	132531 (113298,153196)	3086.21 (2645.23,3555.66)	8.79 (8.33,10.13)
Bosnia and Herzegovina	122969 (104446,142468)	2878.62 (2454.01,3322.66)	188335 (161913,216493)	3128.74 (2693.56,3580.86)	8.69 (7.77,9.76)
Bulgaria	383783 (325745,440869)	3071.08 (2628.8,3528.5)	432007 (369658,503657)	3211.35 (2748.52,3723.95)	4.57 (4.55,5.54)
Croatia	187930 (161636,216927)	3012.74 (2599.33,3468.96)	265456 (228061,305841)	3219.92 (2754.19,3694.81)	6.88 (5.96,6.51)
Czechia	411217 (349506,475727)	3034.57 (2583.68,3487.64)	648726 (562837,742368)	3247.81 (2808.98,3716.97)	7.03 (6.58,8.72)
Hungary	453680 (389284,518086)	3117.21 (2672.31,3574.57)	600646 (515082,696103)	3290.05 (2822.44,3807.84)	5.54 (5.62,6.53)
Montenegro	19530 (16701,22530)	3077.25 (2641.01,3540.13)	31260 (26512,35974)	3218.33 (2735.58,3727.31)	4.58 (3.58,5.29)
North Macedonia	56816 (48316,64965)	2959.68 (2530.05,3406.64)	106211 (90264,122095)	3154.97 (2699.56,3630.34)	6.6 (6.57,6.7)
Poland	1301420 (1114310,1502244)	2983.22 (2561.68,3434.04)	2204604 (1895877,2552373)	3243.33 (2778.91,3732.74)	8.72 (8.48,8.7)
Romania	837338 (712366,966691)	2960.41 (2536.16,3398.26)	1130574 (971118,1302491)	3219.66 (2772.22,3724.14)	8.76 (9.31,9.59)

Serbia	350644 (297685,401102)	3009.87 (2581.77,3436.16)	503533 (432137,583590)	3224.1 (2767.64,3727.05)	7.12 (7.2,8.47)
Slovakia	180083 (154628,208829)	3039.12 (2611.79,3511.42)	300055 (255138,349169)	3245.2 (2760.55,3758.97)	6.78 (5.7,7.05)
Slovenia	74593 (64009,85641)	3033.02 (2611.61,3475.54)	131975 (114507,152187)	3242.46 (2793.14,3734.55)	6.91 (6.95,7.45)
**Central Asia**	1192276 (1022977,1370278)	2533.07 (2186.47,2911.04)	2301085 (1969099,2659417)	2700.22 (2336.33,3105.34)	6.6 (6.67,6.85)
Armenia	68518 (58314,78562)	2463.37 (2103.7,2820.74)	115968 (100557,133873)	2698.2 (2345.63,3106.37)	9.53 (10.13,11.5)
Azerbaijan	127027 (108248,145409)	2509.29 (2143.68,2856.54)	299059 (252802,345606)	2743.78 (2352.31,3175.04)	9.34 (9.73,11.15)
Georgia	328224 (283077,375067)	2624.23 (2241.46,3023.06)	157520 (134139,183640)	2722.08 (2322.61,3165.66)	3.73 (3.62,4.72)
Kazakhstan	335773 (286944,388021)	2633.54 (2252.16,3050.59)	524726 (445801,602127)	2829.07 (2426.61,3239.31)	7.42 (6.19,7.75)
Kyrgyzstan	73666 (63111,84837)	2478.85 (2124.46,2839.34)	131156 (111755,150331)	2596.13 (2232.44,2957.17)	4.73 (4.15,5.08)
Mongolia	25690 (21820,29436)	2439.88 (2087.52,2792.23)	64605 (54980,74465)	2600.41 (2239.64,2958.12)	6.58 (5.94,7.29)
Tajikistan	64397 (55427,73905)	2349.15 (2031.07,2697.74)	151585 (129545,173088)	2425.49 (2101.17,2773.29)	3.25 (2.8,3.45)
Turkmenistan	48118 (40817,55574)	2511.52 (2134.11,2896.62)	114907 (97831,131377)	2732.86 (2349.02,3125.16)	8.81 (7.89,10.07)
Uzbekistan	283780 (242051,326935)	2469.29 (2114.65,2843.79)	741559 (629539,859047)	2673.49 (2306.68,3116.56)	8.27 (9.08,9.59)
**Central Latin America**	3520770 (3020725,4018304)	4114.19 (3551.89,4687.11)	11498876 (9894419,13149726)	4492.79 (3881.3,5124.51)	9.2 (9.27,9.33)
Colombia	727430 (624241,835622)	3999.73 (3452.66,4596.7)	2456831 (2128652,2827233)	4431.24 (3846.08,5099.92)	10.79 (10.95,11.39)
Costa Rica	72936 (62727,83855)	4125.05 (3551.51,4732.83)	251057 (215967,290299)	4542.72 (3916.42,5249.56)	10.13 (10.27,10.92)
El Salvador	121168 (103491,138779)	4049.15 (3482.57,4631.44)	275821 (240206,314658)	4528.27 (3928.7,5166.45)	11.83 (11.55,12.81)
Guatemala	138417 (117395,159527)	3819.63 (3270.99,4379.74)	477914 (411558,545062)	4239.1 (3649.81,4845.24)	10.98 (10.63,11.58)
Honduras	81897 (69522,93542)	3897.21 (3308.68,4425.53)	285498 (245698,327845)	4310.1 (3699.5,4944.41)	10.59 (11.72,11.81)
Mexico	77342 (66100,88411)	4626.9 (3936.43,5300.32)	178176 (154204,204973)	4904.9 (4213.07,5630.81)	6.01 (6.24,7.03)
Nicaragua	62666 (53894,71621)	3968.23 (3413.06,4543.17)	222878 (190363,254152)	4396.99 (3772.45,5016.02)	10.8 (10.41,10.53)
Panama	58083 (50058,66127)	3840.54 (3316.35,4381.42)	193692 (166733,220672)	4376.1 (3772.28,4986)	13.94 (13.75,13.8)

Venezuela (Bolivarian Republic of)	411280 (353058,471535)	4130.66 (3543.38,4739.14)	1382427 (1185082,1587522)	4467.86 (3847.08,5127.32)	8.16 (8.19,8.57)
**Andean Latin America**	853460 (738034,976452)	4070.11 (3516.08,4660.84)	2758218 (2370967,3163149)	4588.33 (3950.43,5244.03)	12.73 (12.35,12.51)
Bolivia (Plurinational State of)	130682 (111962,149840)	3959.6 (3414.71,4534.59)	423951 (360458,487416)	4473.4 (3834.08,5124.94)	12.98 (12.28,13.02)
Ecuador	231156 (200312,263664)	4258.87 (3688.22,4856.53)	788971 (677373,901237)	4753.34 (4079.78,5423.74)	11.61 (10.62,11.68)
Peru	491622 (422625,565510)	4016.65 (3462.25,4641.56)	1545296 (1319661,1780663)	4540.07 (3880.48,5217.4)	13.03 (12.08,12.41)
**Caribbean**	1066131 (923195,1224744)	4080.07 (3540.14,4677.22)	2403792 (2070228,2740275)	4454.36 (3840.02,5084.92)	9.17 (8.47,8.72)
Antigua and Barbuda	2086 (1804,2393)	4087.85 (3521.38,4684.26)	4929 (4228,5633)	4425.15 (3806.19,5058.23)	8.25 (7.98,8.09)
Bahamas	6802 (5837,7837)	4317.12 (3712.6,4970.55)	19801 (16847,22654)	4609.41 (3940.04,5254.46)	6.77 (5.71,6.13)
Barbados	11767 (10019,13582)	4329.32 (3701.18,4980.22)	23281 (19855,26498)	4620.98 (3946.01,5269.45)	6.74 (5.81,6.61)
Belize	3834 (3295,4392)	4128.19 (3532.85,4739.46)	14567 (12525,16855)	4621.04 (3999.59,5297.33)	11.94 (11.77,13.21)
Bermuda	2893 (2484,3293)	4572.76 (3936.94,5200.28)	6120 (5223,7046)	4798.09 (4091.41,5509.6)	4.93 (3.92,5.95)
Cuba	415811 (358678,476870)	4064.82 (3496.46,4659.26)	860421 (737457,986448)	4484.75 (3839.78,5121.43)	10.33 (9.82,9.92)
Dominica	2416 (2076,2768)	4196.95 (3615.72,4815.81)	3821 (3272,4371)	4480.28 (3850.37,5117.2)	6.75 (6.26,6.49)
Dominican Republic	150851 (130194,173912)	3966.5 (3421.96,4552.88)	450799 (388215,516222)	4447.93 (3835.78,5095.94)	12.14 (11.93,12.09)
Grenada	2717 (2341,3122)	4007.18 (3436.34,4594.68)	5211 (4441,6001)	4396.1 (3773.14,5053.19)	9.71 (9.8,9.98)
Guyana	15584 (13398,17864)	3998.53 (3428.16,4566.43)	29569 (25431,33923)	4390.4 (3773.35,5000.62)	9.8 (9.51,10.07)
Haiti	123331 (105167,140803)	3701.53 (3171.88,4239.19)	301617 (256108,347558)	3913.76 (3348.87,4499.51)	5.73 (5.58,6.14)
Jamaica	70156 (60503,79975)	4074.34 (3520.67,4626.21)	137720 (118808,158584)	4454.13 (3842.91,5127.03)	9.32 (9.15,10.83)
Puerto Rico	163281 (140856,188940)	4522.24 (3903.55,5228.2)	316244 (272839,362469)	4957.79 (4258.87,5636.66)	9.63 (7.81,9.1)
Saint Kitts and Nevis	1497 (1295,1724)	4228.46 (3614.57,4832.65)	3447 (2920,3977)	4618.37 (3958.03,5318.13)	9.22 (9.5,10.05)
Saint Lucia	3554 (3047,4087)	4095.87 (3500.68,4726.59)	11015 (9383,12614)	4491.77 (3830.98,5135.22)	9.67 (8.65,9.44)

Saint Vincent and the Grenadines	2808 (2390,3226)	4013.87 (3421.05,4615.59)	6430 (5479,7357)	4414.21 (3776.78,5031.68)	9.97 (9.01,10.4)
Suriname	10740 (9261,12315)	4089.85 (3536.02,4681.38)	29546 (25322,33817)	4523.48 (3885.29,5157.56)	10.6 (9.88,10.17)
Trinidad and Tobago	35869 (30887,41681)	4225.65 (3631.05,4902.09)	89459 (76903,103166)	4589.04 (3951.12,5280.51)	8.6 (7.72,8.81)
United States Virgin Islands	4033 (3444,4683)	4485.92 (3846.08,5172.98)	8448 (7205,9728)	4854.26 (4165.75,5513.4)	8.21 (6.58,8.31)
**Tropical Latin America**	3760894 (3229593,4307856)	4003.22 (3445.06,4588.46)	11648775 (10018943,13328778)	4455.51 (3838.66,5095.51)	11.3 (11.05,11.43)
Brazil	3669907 (3151434,4202859)	4002.44 (3443.05,4588.75)	11386850 (9787425,13035647)	4456.95 (3837.48,5098.63)	11.36 (11.11,11.46)
Paraguay	90987 (77404,103800)	4038.13 (3448.24,4602.39)	261925 (225199,300001)	4386.47 (3780,5030.18)	8.63 (9.29,9.62)
**East Asia**	42602441 (35969751,49229166)	4662.94 (3992.86,5359.08)	113375622 (96002567,130995916)	5016.78 (4267.39,5758.88)	7.59 (6.88,7.46)
China	41044009 (34636657,47406915)	4667.29 (3996.06,5359.85)	109575472 (92723351,126639049)	5016.52 (4265.22,5758.38)	7.48 (6.74,7.44)
Democratic People's Republic of Korea	799391 (679386,927274)	4555.29 (3917.5,5249.59)	1631136 (1388699,1880963)	4751.33 (4072.88,5461.25)	4.3 (3.97,4.03)
Taiwan (Province of China)	759041 (644296,879006)	4575.66 (3911,5280.57)	2169014 (1859175,2462678)	5263.61 (4510.48,5989.03)	15.03 (13.42,15.33)
**Southeast Asia**	7773368 (6600060,8940996)	2885.33 (2480.85,3300.1)	22706789 (19313885,26216287)	3238.01 (2773.46,3719.63)	12.22 (11.79,12.71)
Cambodia	138530 (117410,160560)	2900.88 (2486.87,3361.62)	415435 (352913,480035)	3138.79 (2687.24,3602.34)	8.2 (7.16,8.06)
Indonesia	3019331 (2558413,3482089)	2872.4 (2464.84,3286.94)	8455534 (7153574,9778458)	3186.42 (2737.63,3663.66)	10.93 (11.07,11.46)
Lao People's Democratic Republic	62479 (53298,72328)	2883.96 (2473.46,3315.61)	160168 (135102,185302)	3162.03 (2704.98,3639.18)	9.64 (9.36,9.76)
Malaysia	305669 (259783,353648)	3080.82 (2631.03,3564.78)	1029003 (879590,1191162)	3461.55 (2979.9,4005.92)	12.36 (12.37,13.26)
Maldives	2784 (2337,3267)	2792.59 (2383.92,3243.97)	12808 (10811,14913)	3243.86 (2775.43,3750.72)	16.16 (15.62,16.42)
Mauritius	23628 (20026,27150)	3132.58 (2678.79,3587.9)	65395 (56034,75597)	3494.9 (3012.26,4024.03)	11.57 (12.16,12.45)
Myanmar	691310 (587687,802417)	2874.45 (2466.43,3303.11)	1665523 (1409884,1932984)	3210.72 (2736.45,3689.26)	11.7 (10.95,11.69)

Philippines	807456 (684598,931822)	2558.32 (2191.01,2945)	2560788 (2167721,2960350)	2924.64 (2506.01,3372.69)	14.32 (14.38,14.52)
Seychelles	1791 (1532,2059)	3244.9 (2768.71,3727.15)	4398 (3707,5069)	3509.68 (2993.61,4022.97)	8.16 (7.94,8.12)
Sri Lanka	336455 (286663,388295)	2930.89 (2507.46,3385.08)	897956 (765420,1025712)	3262.98 (2799.04,3714.06)	11.33 (9.72,11.63)
Thailand	1209104 (1018955,1397805)	3150.75 (2693.63,3612.62)	4101374 (3501606,4731345)	3720.96 (3189.55,4297.32)	18.1 (18.41,18.95)
Timor-Leste	9096 (7699,10548)	2808.1 (2410.67,3230.36)	26093 (22373,30144)	2972.71 (2557.3,3426.43)	5.86 (6.07,6.08)
Viet Nam	1154490 (988840,1322473)	2872.27 (2466.33,3287.59)	3280643 (2784356,3782298)	3098.78 (2649.06,3558.56)	7.89 (7.41,8.24)
**Oceania**	119873 (101127,139191)	3730.88 (3201.2,4301.16)	338675 (285507,391076)	4025.21 (3433.77,4602.96)	7.89 (7.02,7.27)
American Samoa	1082 (911,1250)	4394.39 (3743.9,5036.7)	2492 (2123,2884)	4794.96 (4115.6,5519.1)	9.12 (9.58,9.93)
Cook Islands	554 (469,639)	4198.12 (3598.36,4814.78)	1244 (1061,1434)	4844.99 (4139.89,5580.52)	15.41 (15.05,15.9)
Fiji	15877 (13463,18425)	3988.89 (3409.05,4622.44)	37918 (32061,44138)	4554.43 (3901.75,5281.44)	14.18 (14.26,14.45)
Guam	3526 (2985,4083)	4173.95 (3561.64,4824.89)	9901 (8429,11455)	4696.5 (4007.64,5400.01)	12.52 (11.92,12.52)
Kiribati	1544 (1309,1786)	3980.87 (3416.91,4587.81)	3480 (2939,4001)	4389.98 (3768.05,5002.06)	10.28 (9.03,10.28)
Marshall Islands	627 (535,724)	3729.59 (3200.37,4300.07)	1645 (1399,1887)	4146.65 (3550.51,4724.54)	11.18 (9.87,10.94)
Micronesia (Federated States of)	1962 (1682,2255)	3993.07 (3452.23,4590.43)	3622 (3047,4189)	4425.29 (3769.33,5052.05)	10.82 (9.19,10.06)
Nauru	199 (168,229)	3940.32 (3377.82,4529.85)	286 (242,331)	4546.58 (3873.76,5250.08)	15.39 (14.68,15.9)
Niue	90 (77,103)	4188.29 (3585.03,4838.67)	104 (89,119)	4720.19 (4062.7,5402.23)	12.7 (11.65,13.32)
Northern Mariana Islands	895 (754,1042)	4143.3 (3566.19,4744.05)	2728 (2294,3164)	4584.79 (3944.31,5259.22)	10.66 (10.6,10.86)
Palau	423 (361,487)	4188.61 (3590.68,4797.4)	1190 (997,1372)	4641.84 (3959.09,5315.6)	10.82 (10.26,10.8)
Papua New Guinea	71082 (60102,83051)	3593.31 (3080.04,4153.91)	223924 (188761,259235)	3845.92 (3289.1,4416.57)	7.03 (6.32,6.79)
Samoa	3688 (3098,4233)	4174.39 (3554.97,4780.1)	6827 (5763,7893)	4479 (3815.11,5165.2)	7.3 (7.32,8.06)
Solomon Islands	5432 (4579,6317)	3645.58 (3116.34,4199.5)	15814 (13333,18402)	4098.69 (3516.91,4709.91)	12.43 (12.15,12.85)

Tokelau	52 (44,60)	3946.29 (3368.13,4530.32)	66 (57,76)	4500.31 (3852.28,5158.46)	14.04 (13.87,14.37)
Tonga	2384 (2019,2773)	4107.59 (3498.54,4753.39)	3762 (3209,4318)	4557.91 (3901.75,5221.75)	10.96 (9.85,11.53)
Tuvalu	275 (232,315)	3948.23 (3378.26,4519.12)	473 (404,544)	4380.84 (3762.6,5011.29)	10.96 (10.89,11.38)
Vanuatu	2533 (2142,2926)	3741.42 (3186.53,4293.62)	7991 (6728,9257)	4171.74 (3566.38,4792.92)	11.5 (11.63,11.92)
**North Africa and Middle East**	5916998 (5065219,6807128)	3378.07 (2909.97,3880.84)	18591752 (15988433,21426733)	3810.43 (3275.1,4375)	12.8 (12.55,12.73)
Afghanistan	218392 (185106,252498)	3054.97 (2604.28,3512.71)	367487 (311043,426796)	3399.78 (2909.72,3904.96)	11.29 (11.17,11.73)
Algeria	423263 (359168,491821)	3327.58 (2843.92,3847.89)	1447429 (1231558,1671316)	3811.37 (3254.92,4380.45)	14.54 (13.84,14.45)
Bahrain	7290 (6128,8460)	3597.7 (3077.58,4169.88)	47506 (39539,54790)	3982.96 (3410.03,4546.62)	10.71 (9.03,10.8)
Egypt	999604 (848502,1165571)	3457.09 (2971.6,3969.69)	2702515 (2292126,3154778)	3883.05 (3322.01,4491.44)	12.32 (11.79,13.14)
Iran (Islamic Republic of)	880006 (743025,1011517)	3227.86 (2770.04,3703.38)	2940263 (2520062,3368168)	3595.94 (3094.19,4140.59)	11.4 (11.7,11.81)
Iraq	288596 (246516,331137)	3571.54 (3054.85,4073.3)	992738 (846877,1149354)	3803.13 (3226.04,4377.09)	6.48 (5.6,7.46)
Jordan	49993 (43025,57617)	3479.83 (2986.01,4001.22)	332662 (283508,385073)	3956.28 (3378.63,4517.59)	13.69 (12.91,13.15)
Kuwait	25658 (21586,29713)	3629.92 (3098.88,4194.06)	152120 (127811,177115)	4103.51 (3499.68,4741.94)	13.05 (12.93,13.06)
Lebanon	75840 (64454,88592)	3363.65 (2879.78,3912.16)	224446 (193463,257843)	3837.62 (3289.82,4415.64)	14.09 (12.87,14.24)
Libya	69287 (59210,80370)	3540.68 (3024.61,4071.73)	226868 (191953,260963)	3883.93 (3318.01,4443.16)	9.69 (9.12,9.7)
Morocco	474891 (404637,543312)	3274.9 (2802.47,3732.28)	1335410 (1140249,1551251)	3713.8 (3186.98,4316.77)	13.4 (13.72,15.66)
Oman	23778 (20222,27661)	3201.73 (2748.75,3676.21)	97626 (83255,112954)	3896.83 (3357.37,4450.96)	21.71 (21.07,22.14)
Palestine	30128 (25618,34815)	3423.01 (2919.05,3960.81)	102885 (88018,118574)	3685.71 (3182.02,4239.86)	7.67 (7.05,9.01)
Qatar	5840 (4819,6841)	3671.08 (3118.6,4257.86)	64293 (53657,74410)	4116.78 (3529.89,4751.62)	12.14 (11.6,13.19)
Saudi Arabia	215917 (182941,250091)	3414.2 (2894.75,3944.19)	987955 (828285,1149038)	4023.43 (3436.35,4620.42)	17.84 (17.14,18.71)
Sudan	292018 (250113,336125)	3075.36 (2630.42,3533.51)	758628 (647046,878301)	3591.14 (3065.99,4121.58)	16.77 (16.56,16.64)
Syrian Arab	183305 (156684,211533)	3378.1	539745	3728.93	10.39

Republic		(2912.62,3887.75)	(456008,625242)	(3179.23,4297.81)	(9.15,10.55)
Tunisia	173096 (146930,199767)	3350.37 (2864.68,3860.78)	516592 (444113,594564)	3757.41 (3239.13,4299.18)	12.15 (11.36,13.07)
Türkiye	1302667 (1111261,1500768)	3616.91 (3108.74,4185.48)	3933251 (3382509,4552904)	4066.07 (3515.09,4690.46)	12.42 (12.07,13.07)
United Arab Emirates	20717 (17267,24217)	3450.79 (2950.47,3957.2)	302515 (250446,355876)	3885.45 (3316.69,4445.81)	12.6 (12.35,12.41)
Yemen	153476 (130282,177993)	3013.69 (2573.39,3485.51)	501476 (428203,577144)	3301.67 (2826.77,3793.51)	9.56 (8.84,9.85)
**South Asia**	21001741 (17861857,24088981)	3441.76 (2953.66,3924.8)	58791056 (50326960,67052871)	3818.04 (3282.09,4349.41)	10.93 (10.82,11.12)
Bangladesh	1608381 (1367349,1839584)	3272.66 (2812.54,3741.38)	5140114 (4372193,5862993)	3582.95 (3067.62,4069.09)	9.48 (8.76,9.07)
Bhutan	9088 (7710,10397)	3478.23 (2982.99,3989.31)	24402 (20903,27857)	3880.5 (3327.27,4423.54)	11.57 (10.88,11.54)
India	17412254 (14805069,19971633)	3538.82 (3043.58,4028.26)	48463957 (41607887,55215872)	3911.42 (3368.5,4448.98)	10.53 (10.44,10.68)
Nepal	331909 (283442,376808)	3316.5 (2847.56,3760.78)	912049 (783876,1044685)	3762.47 (3255.34,4301.22)	13.45 (14.32,14.37)
Pakistan	1640110 (1366597,1932085)	2821.45 (2349.77,3324.24)	4250535 (3535463,4966744)	3213.7 (2693.11,3748.38)	13.9 (12.76,14.61)
**Southern Sub-Saharan Africa**	1002298 (855477,1155712)	3626.52 (3109.81,4181.9)	2363886 (2022846,2722791)	3913.26 (3363.99,4509.41)	7.91 (7.83,8.17)
Botswana	19845 (17007,22822)	3423.94 (2945.33,3923.76)	62286 (53272,71430)	3911.53 (3359.74,4485.45)	14.24 (14.07,14.32)
Eswatini	11263 (9538,13118)	3723.31 (3158.77,4292.29)	24040 (20368,27558)	4033.25 (3425.81,4640.02)	8.32 (8.1,8.45)
Lesotho	29321 (25144,33836)	3399.01 (2906.22,3921.06)	41892 (35678,48001)	3750.94 (3233.81,4282.48)	10.35 (9.22,11.27)
Namibia	22687 (19337,26266)	3394.59 (2916.9,3910.94)	53863 (46328,62343)	3679.63 (3168.58,4214.31)	8.4 (7.76,8.63)
South Africa	774248 (658540,891776)	3685.35 (3152.19,4255.43)	1917337 (1640743,2211862)	3971.89 (3413.32,4567.72)	7.78 (7.34,8.28)
Zimbabwe	144933 (124368,166808)	3432.78 (2951.07,3931.44)	264469 (224604,304783)	3569.41 (3056.52,4110.59)	3.98 (3.57,4.56)
**Western Sub-Saharan Africa**	3179864 (2716760,3652589)	3496.16 (2995.24,4022.03)	8096695 (6882245,9292419)	3801.12 (3250.23,4369.9)	8.72 (8.51,8.65)
Benin	70618 (59924,81702)	3499.85 (2974.17,4036.08)	216260 (182858,250330)	3906.34 (3333.79,4504.34)	11.61 (11.6,12.09)
Burkina Faso	151875 (129801,173958)	3394.55 (2920.73,3893.13)	364727 (310444,421219)	3723.39 (3164.93,4270.1)	9.69 (8.36,9.68)
Cabo Verde	7804 (6681,9042)	3563.58	18400 (15508,21328)	3965.99	11.29

		(3056.62,4129.19)		(3358.58,4574.49)	(9.88,10.78)
Cameroon	177268 (150249,204928)	3781.2 (3215.34,4364.02)	555674 (474232,637988)	4066.17 (3487.26,4668.74)	7.54 (6.98,8.46)
Chad	94371 (80813,108902)	3317.42 (2831.87,3828.17)	216396 (184901,251753)	3484.52 (2964.82,4041.53)	5.04 (4.69,5.57)
Côte d'Ivoire	153955 (131252,178607)	3537.6 (3033.48,4082.15)	471219 (400510,544117)	3809.3 (3269.11,4418.26)	7.68 (7.77,8.23)
Gambia	12712 (10742,14701)	3468.34 (2937.94,4009.01)	39169 (33424,45070)	3806.01 (3241.59,4367.38)	9.74 (8.94,10.34)
Ghana	228914 (193866,266163)	3480.1 (2980.07,4018.7)	717795 (608288,827958)	3971.54 (3381.31,4554.93)	14.12 (13.34,13.46)
Guinea	115448 (98317,133364)	3428.51 (2922.32,3944.21)	215046 (183953,247996)	3648.64 (3142.27,4175.64)	6.42 (5.87,7.53)
Guinea-Bissau	14078 (11919,16275)	3439.33 (2918.81,3995.45)	28800 (24420,33128)	3653.92 (3115.86,4212.35)	6.24 (5.43,6.75)
Liberia	41505 (35542,47709)	3563.1 (3048.1,4092.98)	92742 (78346,107508)	3901.49 (3335.12,4544.29)	9.5 (9.42,11.03)
Mali	139968 (118141,162045)	3359.76 (2871.21,3856.83)	340281 (288934,390685)	3609.45 (3102.06,4120.09)	7.43 (6.83,8.04)
Mauritania	37455 (31975,42960)	3681.67 (3148.52,4228.43)	91359 (78215,105768)	4025.79 (3469.67,4648.61)	9.35 (9.94,10.2)
Niger	98866 (84284,114015)	3346.16 (2880,3864.36)	311987 (265585,363171)	3542.89 (3041.4,4119.44)	5.88 (5.6,6.6)
Nigeria	1598941 (1363956,1833070)	3506.89 (3006.89,4024.57)	3796910 (3214277,4359135)	3807.39 (3253.69,4369.2)	8.57 (8.21,8.56)
Sao Tome and Principe	2343 (1994,2705)	3593.16 (3077.25,4145.25)	4777 (4076,5501)	3932.58 (3369.85,4520.61)	9.45 (9.06,9.51)
Senegal	117902 (99921,136625)	3544.71 (3032.63,4108.31)	310501 (262844,358435)	3792.46 (3213.76,4355.86)	6.99 (5.97,6.03)
Sierra Leone	70651 (60694,80839)	3413.85 (2927.59,3912.54)	146761 (125017,168931)	3661.3 (3122.7,4203.61)	7.25 (6.66,7.44)
Togo	45085 (38405,51860)	3450.78 (2961.82,3982.21)	157798 (133712,181501)	3740.77 (3218.5,4314.53)	8.4 (8.35,8.67)
**Eastern Sub-Saharan Africa**	2491439 (2121796,2866357)	3216.44 (2769.51,3707.07)	6218769 (5299896,7142986)	3446.18 (2959.9,3965.07)	7.14 (6.87,6.96)
Burundi	75209 (64211,86182)	3190.83 (2734.12,3659.71)	170394 (144398,197028)	3240.87 (2766.07,3732.76)	1.57 (1.17,2)
Comoros	6772 (5758,7875)	3296.43 (2822.19,3789.54)	18135 (15508,20730)	3509.56 (3018.15,4002.97)	6.47 (5.63,6.94)
Djibouti	4798 (4049,5560)	3190.35 (2728.24,3637.24)	25514 (21550,29498)	3528.87 (3023.13,4071.53)	10.61 (10.81,11.94)
Eritrea	38289 (32156,44386)	3122.88 (2677.63,3600.21)	100071 (85520,115107)	3297.18 (2851.83,3793.18)	5.58 (5.36,6.51)

Ethiopia	646084 (549386,747051)	3145.96 (2715.19,3634.65)	1528816 (1310890,1762268)	3355.66 (2881.74,3875.43)	6.67 (6.13,6.62)
Kenya	277936 (236953,318257)	3279.31 (2804.39,3775.64)	876838 (744710,1006917)	3569.94 (3058.54,4110.65)	8.86 (8.87,9.06)
Madagascar	164987 (141132,191791)	3165.22 (2723.11,3662.69)	411396 (344172,475845)	3307 (2818.24,3800.32)	4.48 (3.49,3.76)
Malawi	130113 (110363,150091)	3265.16 (2787.72,3761.93)	276585 (234559,317633)	3517.8 (2993.71,4046.61)	7.74 (7.39,7.57)
Mozambique	201668 (171171,231774)	3203.3 (2740.03,3688.16)	411261 (347402,472241)	3444.43 (2930.11,3973.76)	7.53 (6.94,7.74)
Rwanda	94495 (80665,108099)	3255.48 (2794.73,3718.5)	235781 (200526,270812)	3480.18 (2976.96,3987.41)	6.9 (6.52,7.23)
Somalia	83803 (71238,96684)	3206.89 (2738.49,3716.77)	222003 (188308,255289)	3319.84 (2850.49,3818)	3.52 (2.72,4.09)
South Sudan	83561 (71077,96648)	3217.86 (2748.54,3710.36)	147378 (124240,170165)	3433.35 (2917.58,3949.03)	6.7 (6.15,6.43)
Uganda	212429 (182240,245211)	3199.56 (2755.77,3682)	546939 (467171,631007)	3459.97 (2962.97,3971.14)	8.14 (7.52,7.85)
United Republic of Tanzania	371041 (315003,428986)	3309.6 (2822.88,3829.44)	974971 (825614,1125651)	3568.24 (3045.74,4092.26)	7.81 (6.86,7.89)
Zambia	98475 (83748,113144)	3303.37 (2809.17,3804.77)	267275 (227279,309997)	3534.91 (3047.13,4080.59)	7.01 (7.25,8.47)
**Central Sub-Saharan Africa**	776748 (661806,895686)	3288.2 (2816.19,3766.21)	2053774 (1756352,2357159)	3433.3 (2947.25,3926.7)	4.41 (4.26,4.65)
Angola	131942 (111407,153171)	3185.91 (2727.69,3659.36)	453310 (382775,524462)	3498.42 (2993.53,3990.41)	9.81 (9.05,9.75)
Central African Republic	38317 (32303,44293)	3199.62 (2730.82,3666.4)	80806 (68444,93300)	3324.43 (2842.53,3830.04)	3.9 (4.09,4.46)
Congo	37480 (31769,43178)	3408.12 (2908.81,3910.41)	109711 (92786,126668)	3655.4 (3143.98,4173.09)	7.26 (6.72,8.08)
Democratic Republic of the Congo	542601 (461159,627481)	3305.79 (2828.21,3807.15)	1344398 (1147927,1542546)	3382.47 (2897.15,3861.86)	2.32 (1.44,2.44)
Equatorial Guinea	6511 (5510,7588)	3228.6 (2764.52,3734.87)	21470 (18195,24755)	3849.83 (3292.97,4445.22)	19.24 (19.02,19.12)
Gabon	19897 (16867,22919)	3457.5 (2948.04,3982.4)	44078 (37661,51154)	3905.88 (3372.92,4537.44)	12.97 (13.94,14.41)

Table S2: Incidence cases of Osteoarthritis knee disease in 1990 and 2021 the percentage change in the age-standardised rates (ASRs) per 100,000, by location.

	1990		2021		Percentage change in the ASRs per 100,000
	No (95% UI)	ASRs per 100,000 (95% UI)	No (95% UI)	ASRs per 100,000 (95% UI)	
Global	14134284 (12150933,16079381)	330.26 (284.34,375.75)	30845891 (26534151,35188905)	353.67 (304.56,402.5)	7.09 (7.11,7.12)
High-income North America	1160725 (1017358,1319031)	367.48 (317.93,419.67)	2103504 (1830759,2428582)	389.08 (335.14,444.02)	5.88 (5.41,5.8)
Canada	66728 (58059,76170)	212.78 (184.78,244.68)	142784 (123124,164885)	242.01 (210.54,275.05)	13.74 (12.41,13.94)
Greenland	102 (88,117)	230.72 (199.7,264.46)	195 (168,231)	259.22 (226.22,296.58)	12.35 (12.15,13.28)
United States of America	1093868 (959538,1243508)	384.67 (332.96,439.61)	1960492 (1707253,2261850)	406.51 (350.48,464.22)	5.68 (5.26,5.6)
**Australasia**	80753 (70372,92095)	359.26 (310.77,412.04)	177000 (153385,205240)	403.34 (348.15,463.45)	12.27 (12.03,12.48)
Australia	67582 (58768,76997)	359.63 (310.78,412.57)	148591 (128797,171863)	404.82 (349.2,465.56)	12.57 (12.36,12.84)
New Zealand	13171 (11494,15112)	357.26 (308.1,411.27)	28409 (24525,32809)	395.46 (342.58,452.77)	10.69 (10.09,11.19)
**High-income Asia Pacific**	928187 (799581,1062050)	441.54 (382.22,504.23)	1482839 (1312650,1690590)	458.22 (397.65,522.59)	3.78 (3.64,4.04)
Brunei Darussalam	606 (522,688)	434.19 (378.46,496.57)	2159 (1866,2465)	468.07 (407.83,533.21)	7.8 (7.38,7.76)
Japan	740432 (636892,848733)	432.03 (373.15,493.42)	1005565 (887299,1143421)	441.94 (382.84,503.95)	2.29 (2.13,2.6)
Singapore	12026 (10304,13776)	446.98 (385.51,511.34)	41341 (35907,47285)	467.22 (405.99,533.81)	4.53 (4.39,5.31)
Republic of Korea	175124 (150561,200592)	480.61 (416.52,552.19)	433774 (378392,498229)	491.74 (427.19,560.51)	2.32 (1.51,2.56)
**Western Europe**	1708316 (1486092,1964832)	334.48 (289.7,381.98)	2555151 (2238318,2953418)	357.68 (311.46,406.98)	6.94 (6.54,7.51)
Andorra	199 (172,232)	329.64 (283.88,385.94)	521 (449,604)	356.21 (308.03,408.4)	8.06 (5.82,8.51)
Austria	34076 (29563,38990)	332.33 (288.3,378.14)	51703 (44889,59759)	355.39 (308.57,407.86)	6.94 (7.03,7.86)
Belgium	44565 (38478,51315)	328.54 (282.48,378.48)	63987 (55538,73570)	352.59 (306.19,401.86)	7.32 (6.18,8.39)
Cyprus	2650 (2293,3040)	319.93 (277.09,365.96)	6708 (5888,7658)	347.41 (303.87,398.15)	8.59 (8.8,9.66)
Denmark	22161 (19186,25324)	319.51	31897 (27875,36767)	344	7.66 (7.03,7.71)

		(277.62,366.22)		(299.02,391.96)	
Finland	22072 (19332,25199)	334.07 (291,383)	32672 (28598,37499)	356.47 (311.02,409.69)	6.71 (6.88,6.97)
France	237055 (206503,272083)	327.17 (282.23,374.9)	366042 (317330,422304)	351.29 (305.13,401.98)	7.37 (7.22,8.11)
Germany	377450 (326328,436453)	335.54 (290.6,383.76)	518788 (453049,601412)	357.4 (310.48,407.04)	6.51 (6.07,6.84)
Greece	46996 (40857,54487)	333.97 (291.04,383.04)	62266 (54254,71220)	356.86 (309.41,407.92)	6.85 (6.31,6.5)
Iceland	881 (769,1013)	336.87 (292.31,386.55)	1781 (1549,2038)	361.71 (314.16,412.49)	7.37 (6.71,7.47)
Ireland	12533 (10873,14416)	332.84 (287.47,384.13)	25223 (22007,29036)	357.97 (311.27,409.67)	7.55 (6.65,8.28)
Israel	15259 (13306,17554)	331.99 (286.14,381.98)	38576 (33465,44150)	355.97 (309.05,408.55)	7.22 (6.96,8.01)
Italy	265489 (230968,305715)	333.01 (288.6,379.73)	379595 (332840,437987)	351.76 (306.14,401.52)	5.63 (5.74,6.08)
Luxembourg	1694 (1470,1943)	333.66 (289.06,383.24)	3394 (2991,3903)	353.59 (310.7,403.24)	5.97 (5.22,7.49)
Malta	1417 (1232,1631)	329.49 (285.31,380.29)	2630 (2309,3012)	354.38 (310.46,404.66)	7.55 (6.41,8.81)
Monaco	181 (158,209)	346.28 (299.5,400.34)	260 (226,301)	366.8 (317.43,417.24)	5.93 (4.22,5.99)
Netherlands	63111 (54668,72071)	344.33 (296.72,393.23)	103319 (89701,119147)	367.58 (317.34,418.82)	6.75 (6.51,6.95)
Norway	17919 (15722,20467)	324.99 (281.18,369.91)	28578 (24956,32980)	348.03 (303.04,397.69)	7.09 (7.51,7.77)
Portugal	42763 (36956,49397)	328.8 (286.14,378.04)	66043 (58141,76227)	356.7 (311.91,406.82)	8.49 (7.61,9.01)
San Marino	104 (91,120)	338.5 (294.88,386.54)	206 (179,238)	363.14 (313.94,414.45)	7.28 (6.46,7.22)
Spain	164279 (142313,190084)	333.2 (287.55,384.57)	274790 (239368,316480)	355.54 (311.34,407.8)	6.7 (6.04,8.27)
Sweden	33469 (29005,39221)	272.38 (232.79,319.82)	47999 (41451,56247)	296.18 (252.23,346.27)	8.74 (8.27,8.35)
Switzerland	29917 (25920,34432)	329.14 (282.45,380.2)	49808 (43142,57702)	346.06 (298.82,398.71)	5.14 (4.87,5.8)
United Kingdom	270669 (236137,311172)	355.05 (308.14,405.62)	396116 (346846,456600)	383.77 (333.09,436.78)	8.09 (7.68,8.1)
**Southern Latin America**	163441 (141110,187399)	348.5 (300.96,399.28)	315715 (274143,360911)	387.34 (336.02,441.16)	11.14 (10.49,11.65)
Uruguay	12477 (10831,14376)	348.58 (302.8,399.49)	17665 (15404,20210)	388.86 (336.29,444.78)	11.56 (11.06,11.34)

Argentina	112317 (96777,128791)	345.81 (298.79,396.14)	202138 (175236,230539)	383.85 (331.09,438.73)	11 (10.75,10.81)
Chile	38639 (33521,44445)	356.36 (309.54,409.76)	95895 (83094,111518)	394.63 (343.81,454.98)	10.74 (11.04,11.07)
**Eastern Europe**	769391 (659450,894233)	278.19 (239.66,320.43)	958135 (823739,1109492)	298.56 (256.44,342.29)	7.32 (6.82,7)
Belarus	35178 (30289,41125)	277.89 (239.63,319.39)	43766 (37720,51287)	297.84 (256.24,342.19)	7.18 (6.93,7.14)
Estonia	5577 (4817,6459)	281.57 (243.97,325.76)	6330 (5476,7247)	303.49 (260.51,349.21)	7.78 (6.78,7.2)
Latvia	9774 (8314,11375)	283.84 (242.68,325.97)	9390 (8084,10966)	302.49 (259.76,345.39)	6.57 (5.96,7.04)
Lithuania	12365 (10650,14311)	281.59 (243.45,322.43)	13636 (11779,15990)	299.75 (258.41,345.15)	6.45 (6.14,7.05)
Republic of Moldova	12497 (10698,14515)	272.38 (235.06,314.72)	16544 (14272,19234)	297.29 (256.34,342.44)	9.15 (8.81,9.05)
Russian Federation	499866 (427543,578018)	277.26 (238.61,319.18)	663536 (571706,769157)	300.35 (258.12,344.46)	8.33 (7.92,8.18)
Ukraine	194133 (165380,228725)	280.6 (240.54,326.1)	204934 (175903,236527)	292.95 (249.91,335.84)	4.4 (2.99,3.9)
**Central Europe**	392665 (336818,453197)	263.75 (226.58,302.68)	529220 (459378,611643)	282.04 (242.21,323.09)	6.93 (6.74,6.9)
Albania	5841 (5023,6691)	250.22 (216.44,287.34)	10669 (9191,12473)	271.28 (232.62,313.43)	8.42 (7.48,9.08)
Bosnia and Herzegovina	11681 (9831,13553)	253.94 (217.06,292.03)	14976 (12867,17359)	273.96 (234.64,315.52)	7.88 (8.04,8.1)
Bulgaria	32546 (27920,38042)	268.85 (232.4,308.34)	32938 (28790,38021)	279.87 (242.2,321.54)	4.1 (4.22,4.28)
Croatia	16600 (14204,19228)	263.62 (227.64,302.03)	19979 (17218,23146)	280.6 (241.43,322.44)	6.44 (6.06,6.76)
Czechia	34692 (29852,39891)	265.56 (227.18,306.26)	49899 (43491,57277)	282.63 (243.55,323.29)	6.43 (5.56,7.21)
Hungary	37877 (32545,44157)	272.09 (234.56,314.58)	46060 (39846,53765)	285.94 (245.54,331.06)	5.09 (4.68,5.24)
Montenegro	1751 (1499,2027)	269.45 (230.85,309.83)	2572 (2211,2999)	280.74 (241.89,325.06)	4.19 (4.78,4.92)
North Macedonia	5269 (4501,6201)	260.68 (223.39,304.77)	9099 (7788,10594)	275.71 (236.3,317.8)	5.77 (4.28,5.78)
Poland	113616 (97853,131066)	263.46 (227.16,303.92)	174134 (151398,201862)	284.62 (245.14,327.01)	8.03 (7.6,7.92)
Romania	73125 (62201,86273)	260.31 (222.96,302.07)	87688 (75710,100657)	280.65 (241,321.27)	7.81 (6.36,8.09)
Serbia	31477 (26716,36713)	264.01 (225.94,302.23)	39275 (33813,45400)	280.95 (240.51,322.92)	6.42 (6.45,6.85)

Slovakia	15502 (13435,17823)	266.14 (228.74,306.2)	24243 (20800,28253)	282.27 (241.52,328.41)	6.06 (5.59,7.25)
Slovenia	6409 (5534,7439)	265.44 (228.63,306.55)	9986 (8633,11637)	282.27 (242.27,327.33)	6.34 (5.97,6.78)
**Central Asia**	111442 (96037,128400)	225.44 (194.93,257.32)	222031 (188796,255746)	239.29 (205.97,274.09)	6.14 (5.66,6.52)
Armenia	6470 (5563,7648)	220.31 (190.66,254.24)	9809 (8459,11455)	239.62 (209.11,276.64)	8.76 (8.81,9.68)
Azerbaijan	12041 (10164,13995)	223.76 (191.09,256.48)	28981 (24494,33705)	243.18 (209.77,280.09)	8.68 (9.21,9.78)
Georgia	14542 (12524,17075)	384.95 (332.58,440.77)	12757 (10940,14968)	409.14 (353.99,467.56)	6.28 (6.08,6.44)
Kazakhstan	31618 (27246,36362)	233.2 (201.32,268.54)	49374 (42028,56635)	249.14 (214.38,285.64)	6.84 (6.37,6.49)
Kyrgyzstan	6808 (5858,7933)	221.13 (191.87,255.89)	13014 (11188,14968)	231.57 (199.58,266.61)	4.72 (4.02,4.19)
Mongolia	2495 (2136,2874)	218.32 (187.25,253.5)	6736 (5790,7796)	231.84 (200.31,266.61)	6.19 (5.17,6.97)
Tajikistan	6133 (5271,7027)	211 (181.5,241.1)	15836 (13484,18307)	218.48 (189.9,250.18)	3.55 (3.77,4.63)
Turkmenistan	4671 (3987,5379)	223.75 (192.65,258.01)	11259 (9509,13163)	241.92 (207.8,277.27)	8.12 (7.46,7.86)
Uzbekistan	26663 (22906,30751)	220.6 (190.52,252.82)	74265 (63347,86037)	237.73 (203.94,274.72)	7.77 (7.04,8.66)
**Central Latin America**	340356 (292287,386909)	354.35 (305.58,404.67)	1014222 (871109,1156490)	383.48 (330.54,435.99)	8.22 (7.74,8.17)
Colombia	70664 (61360,80548)	343.74 (296.52,393.38)	207041 (179811,238409)	376.06 (326.06,431.58)	9.4 (9.71,9.96)
Costa Rica	6787 (5872,7723)	353.34 (306.21,404.11)	21166 (18367,24367)	384.54 (333.55,440.64)	8.83 (8.93,9.04)
El Salvador	11089 (9569,12635)	348.06 (299.85,398.25)	23114 (20070,26654)	384.55 (333.76,443.26)	10.48 (11.3,11.31)
Guatemala	13808 (11857,15777)	330.96 (286.41,380.32)	43808 (37757,50068)	363.33 (311.83,417.15)	9.78 (8.88,9.68)
Honduras	7941 (6773,9129)	336.35 (288.67,388.78)	27065 (23336,30822)	367.99 (316.72,420.41)	9.41 (8.14,9.72)
Mexico	6160 (5371,7027)	379.59 (326.86,436.13)	12457 (10807,14262)	404.7 (351.86,461.07)	6.62 (5.72,7.65)
Nicaragua	6133 (5273,6960)	341.27 (294.12,388)	20722 (17775,23742)	373.74 (321.5,430.12)	9.51 (9.31,10.86)
Panama	5418 (4686,6160)	332.06 (286.92,379.71)	16487 (14266,18841)	372.48 (322.36,424.82)	12.17 (11.88,12.35)
Venezuela (Bolivarian Republic of)	39848 (34065,45350)	353 (305.3,403.53)	120295 (102819,136989)	378.51 (325.33,429.38)	7.23 (6.41,6.56)

**Andean Latin America**	80832 (69708,92097)	348.24 (300.96,396.58)	242602 (209062,279223)	386.9 (333.29,443.77)	11.1 (10.74,11.9)
Bolivia (Plurinational State of)	12671 (10970,14571)	339.77 (294.71,393.53)	38829 (33682,44575)	378.27 (329.74,438.3)	11.33 (11.38,11.89)
Ecuador	21814 (18889,24751)	362.28 (314.16,414.57)	68020 (58665,77886)	398.74 (344.44,456.51)	10.06 (9.64,10.12)
Peru	46348 (39804,52965)	344.36 (297.75,395.18)	135754 (116593,156640)	383.76 (329.78,442.03)	11.44 (10.76,11.86)
**Caribbean**	94617 (81658,107550)	348.79 (300.57,396.78)	199696 (172770,230459)	374.83 (324.54,430.56)	7.47 (7.97,8.51)
Antigua and Barbuda	170 (149,193)	349.59 (301.53,401.45)	431 (369,500)	375.78 (323.32,432.73)	7.49 (7.23,7.79)
Bahamas	644 (556,740)	365.86 (315.29,420.77)	1777 (1503,2052)	388.62 (332.86,447.31)	6.22 (5.57,6.31)
Barbados	905 (788,1026)	367.12 (315.19,421.21)	1793 (1538,2091)	389.66 (335.28,447.18)	6.14 (6.17,6.37)
Belize	343 (298,388)	352.71 (305.15,400.64)	1381 (1190,1592)	390.91 (338.05,447.17)	10.83 (10.78,11.61)
Bermuda	252 (217,286)	383.55 (330.03,434.9)	445 (386,514)	401.17 (344.91,460.11)	4.59 (4.51,5.8)
Cuba	35827 (31030,41025)	348.65 (301.68,401.09)	67270 (57860,77896)	379.4 (330.04,436.72)	8.82 (8.88,9.4)
Dominica	198 (172,226)	356.73 (307.94,408.67)	318 (273,370)	377.82 (327.15,433.11)	5.91 (5.98,6.24)
Dominican Republic	14412 (12459,16398)	341.84 (295.45,389.88)	39798 (34175,46250)	377.3 (323.84,440)	10.37 (9.61,12.86)
Grenada	212 (186,242)	343.28 (296.68,394.1)	454 (388,526)	371.76 (321.28,425.89)	8.3 (8.07,8.29)
Guyana	1524 (1320,1737)	343.4 (297.54,394.62)	2700 (2307,3110)	372.81 (322.16,427.05)	8.56 (8.22,8.27)
Haiti	12204 (10542,13996)	321.15 (277.19,367.18)	31043 (26669,35514)	339.2 (294.21,386.43)	5.62 (5.24,6.14)
Jamaica	5792 (5022,6587)	349.04 (299.58,399.91)	11594 (9987,13387)	377.94 (326.02,434.47)	8.28 (8.64,8.83)
Puerto Rico	13663 (11874,15666)	382.14 (331.17,438.91)	21696 (18926,24952)	413.31 (357.6,472.52)	8.16 (7.66,7.98)
Saint Kitts and Nevis	114 (99,130)	359.25 (307.04,414.37)	309 (263,358)	387.77 (334.65,441.85)	7.94 (6.63,8.99)
Saint Lucia	307 (267,351)	350.14 (303.26,400.98)	927 (796,1074)	380.05 (327.06,435.93)	8.54 (7.85,8.72)
Saint Vincent and the Grenadines	238 (207,274)	343.8 (296.04,395.82)	538 (461,627)	375.04 (322.48,431.65)	9.09 (8.93,9.05)
Suriname	990 (849,1141)	350.6 (301.7,402.91)	2549 (2180,2957)	381.91 (328.92,438.35)	8.93 (8.8,9.02)

Trinidad and Tobago	3232 (2793,3666)	359.52 (308.69,410.83)	7315 (6338,8499)	386.59 (335.82,442.11)	7.53 (7.61,8.79)
United States Virgin Islands	384 (330,441)	378.48 (326.94,432.59)	600 (519,700)	405.65 (351.34,466.11)	7.18 (7.46,7.75)
**Tropical Latin America**	362189 (310489,413559)	346.45 (297.89,397.37)	1014593 (871428,1161138)	380.8 (327.9,434.95)	9.91 (9.46,10.07)
Brazil	353720 (303116,404186)	346.47 (297.75,397.59)	990960 (850953,1134433)	381 (328.11,435.26)	9.97 (9.47,10.2)
Paraguay	8468 (7279,9612)	345.61 (296.47,393.69)	23633 (20363,26969)	371.56 (320.57,424.14)	7.51 (7.73,8.13)
**East Asia**	3787399 (3238108,4358778)	377.47 (324.32,433.77)	8800266 (7530686,10174644)	406.19 (348.6,466.86)	7.61 (7.49,7.63)
China	3650857 (3122264,4200440)	377.93 (324.79,434.28)	8512397 (7279974,9840885)	406.42 (348.7,467.23)	7.54 (7.36,7.59)
Democratic People's Republic of Korea	70692 (59950,81929)	364.54 (312.97,417.9)	132067 (113167,153053)	378.9 (325.1,435.11)	3.94 (3.88,4.12)
Taiwan (Province of China)	65849 (56320,76186)	371.4 (319.54,431.4)	155802 (133559,179740)	418.79 (361.81,477.52)	12.76 (10.69,13.23)
**Southeast Asia**	750827 (639280,860351)	247.53 (213.1,282.67)	2050674 (1744848,2371714)	274.07 (235.74,314.07)	10.72 (10.62,11.11)
Cambodia	13597 (11527,15671)	249.14 (212.51,285.96)	38624 (33063,44357)	266.76 (229.25,305.66)	7.07 (6.89,7.88)
Indonesia	299769 (254846,345015)	247.63 (212.7,282.94)	807366 (686278,936248)	273.05 (234.69,312)	10.27 (10.27,10.34)
Lao People's Democratic Republic	5976 (5089,6896)	247.23 (212.25,284.83)	15647 (13248,18150)	269.08 (230.15,312.03)	8.84 (8.43,9.55)
Malaysia	29980 (25583,34541)	261.49 (225.13,301.16)	91610 (78457,106272)	290.78 (250.65,337.31)	11.2 (11.34,12)
Maldives	273 (228,317)	242.63 (206.95,278.22)	1370 (1159,1584)	271.95 (232.49,315.36)	12.08 (12.34,13.35)
Mauritius	2214 (1906,2545)	264.51 (228.41,305.68)	5198 (4431,6028)	292.51 (250.77,336.87)	10.59 (9.79,10.2)
Myanmar	65107 (55500,74676)	246.19 (212.07,283.17)	150459 (128999,174064)	272.5 (234.6,313.41)	10.69 (10.62,10.68)
Philippines	81177 (68633,93294)	222.37 (190.84,255.09)	242884 (205674,280637)	251.52 (216.08,288.24)	13.11 (13,13.23)
Seychelles	148 (128,169)	271.97 (235.02,313.05)	382 (324,442)	292.1 (250.08,336.64)	7.4 (6.41,7.54)
Sri Lanka	32599 (27771,37578)	251.25 (213.72,288.95)	74277 (64182,85392)	276.31 (238.93,317.73)	9.97 (9.96,11.8)
Thailand	115965 (98404,134134)	266.23 (226.95,305.78)	322176 (275913,376817)	309.76 (266.39,357)	16.35 (16.75,17.38)
Timor-Leste	996 (839,1154)	241.5 (206.52,277.57)	2365 (2011,2734)	255.62 (218.14,294.33)	5.85 (5.63,6.04)

Viet Nam	101939 (87431,116993)	245.97 (210.17,281.79)	295456 (250554,341989)	263.31 (225.17,303.44)	7.05 (7.14,7.68)
**Oceania**	11950 (10219,13792)	312.73 (269.5,359.55)	33635 (28494,39060)	334.57 (286.05,387.71)	6.98 (6.14,7.83)
American Samoa	105 (90,121)	358.29 (307.17,411.51)	212 (183,245)	389.31 (336.72,448.09)	8.66 (8.89,9.62)
Cook Islands	50 (42,57)	346.2 (296.82,397.14)	92 (78,107)	394.52 (335.65,451.29)	13.96 (13.08,13.63)
Fiji	1626 (1384,1886)	331.6 (285.07,383.29)	3351 (2857,3902)	370.46 (318.85,427.34)	11.72 (11.49,11.85)
Guam	344 (293,397)	342.98 (293.92,396.51)	749 (642,871)	381.7 (328.51,442.42)	11.29 (11.58,11.77)
Kiribati	149 (127,171)	329.72 (284.92,379.58)	330 (280,380)	360.17 (307.5,414.32)	9.24 (7.93,9.15)
Marshall Islands	63 (54,72)	311.22 (267.81,355.82)	163 (138,188)	345.16 (296.04,394.62)	10.91 (10.54,10.9)
Micronesia (Federated States of)	181 (155,206)	330.43 (283.09,379.43)	329 (277,383)	362.41 (310.29,417.85)	9.68 (9.61,10.13)
Nauru	20 (17,24)	329.57 (284.12,380.75)	28 (24,32)	370.9 (319.45,426.67)	12.54 (12.06,12.43)
Niue	7 (6,8)	343.63 (297.96,396.28)	8 (7,9)	382.24 (329.74,441.54)	11.24 (10.67,11.42)
Northern Mariana Islands	108 (91,126)	338.54 (292.18,387.16)	236 (201,278)	373.88 (321.08,430.24)	10.44 (9.89,11.13)
Palau	39 (34,45)	345.07 (294.59,396.44)	101 (86,120)	376.41 (323.09,435.76)	9.08 (9.67,9.92)
Papua New Guinea	7138 (6097,8260)	303.57 (260.45,348.99)	23184 (19568,26961)	324.49 (277.33,377.32)	6.89 (6.48,8.12)
Samoa	328 (278,377)	343.67 (293.84,393.75)	600 (510,702)	366.61 (313.86,427.44)	6.68 (6.81,8.56)
Solomon Islands	535 (453,623)	308.69 (264.18,357.18)	1602 (1356,1890)	341.61 (292.13,391.5)	10.66 (9.61,10.58)
Tokelau	4 (4,5)	330.37 (284.48,382.44)	5 (5,6)	368.59 (316.91,422.22)	11.57 (10.4,11.4)
Tonga	210 (179,245)	341.15 (292.49,394.48)	320 (273,368)	373.23 (318.03,429.39)	9.4 (8.73,8.85)
Tuvalu	25 (21,29)	329.8 (283.85,380.12)	40 (34,46)	358.25 (308.78,411.47)	8.63 (8.25,8.78)
Vanuatu	255 (216,293)	314.4 (266.57,361.08)	773 (655,899)	346.3 (298.14,399.67)	10.15 (10.69,11.84)
**North Africa and Middle East**	578152 (495303,662749)	292.26 (251.18,334.1)	1811718 (1543012,2081311)	325.34 (279.63,371.94)	11.32 (11.33,11.33)
Afghanistan	19990 (16985,23406)	268.84 (230.88,309.03)	41067 (34271,47879)	294.91 (254.11,337.45)	9.7 (9.2,10.06)

Algeria	39966 (34217,45966)	288.95 (248.23,331.29)	136739 (116454,156716)	326.14 (277.77,375.71)	12.87 (11.9,13.41)
Bahrain	851 (726,976)	305.89 (263.09,351.36)	5249 (4368,6078)	333.98 (287.08,380.46)	9.18 (8.28,9.12)
Egypt	101598 (86890,116817)	298.3 (257.81,344.66)	269387 (226860,312111)	331.78 (285.65,383.43)	11.22 (10.8,11.25)
Iran (Islamic Republic of)	86974 (74332,100448)	282.83 (243.4,324.87)	285543 (244647,326619)	312.39 (268.62,357.31)	10.45 (9.99,10.36)
Iraq	27711 (23676,31513)	305.98 (261.54,350.96)	99904 (84429,115797)	323.86 (275.22,370.11)	5.84 (5.23,5.46)
Jordan	5137 (4356,5858)	299.36 (258.15,339.06)	33489 (28613,38510)	335.18 (290.55,382.63)	11.97 (12.55,12.85)
Kuwait	3049 (2585,3509)	308.26 (263.91,354.69)	17412 (14594,20128)	347.46 (299.8,397.15)	12.72 (11.97,13.6)
Lebanon	7013 (6001,8101)	290.87 (249.39,333.57)	19060 (16367,21887)	327.85 (279.89,380.52)	12.71 (12.23,14.08)
Libya	6677 (5670,7694)	302.97 (258.56,349.68)	23239 (19603,26846)	330.44 (283.41,378.05)	9.07 (8.11,9.61)
Morocco	44946 (38648,51584)	284.74 (244.51,329.85)	121776 (103711,141056)	318.44 (272.04,367.01)	11.84 (11.26,11.27)
Oman	2665 (2277,3064)	276.03 (237.17,317.58)	11710 (9903,13473)	327.76 (282.29,379.71)	18.74 (19.02,19.56)
Palestine	2820 (2410,3231)	295.86 (254.59,340.32)	10367 (8850,11947)	315.79 (270.91,364.09)	6.74 (6.41,6.98)
Qatar	814 (677,953)	309.82 (267.27,357.14)	8832 (7426,10302)	343.01 (294.2,396.91)	10.71 (10.08,11.14)
Saudi Arabia	23227 (19733,26608)	292.96 (252.01,333.13)	118181 (98498,138331)	340.16 (292.65,389.44)	16.11 (16.13,16.9)
Sudan	28618 (24652,32623)	269.7 (231.77,307.97)	78318 (67224,90415)	310.39 (267.81,360.14)	15.09 (15.55,16.94)
Syrian Arab Republic	17878 (15307,20448)	292.6 (251.15,335.16)	50241 (42723,58153)	320.6 (274.64,364.04)	9.57 (8.62,9.35)
Tunisia	16117 (13924,18517)	290.21 (250.92,331.91)	45562 (39223,52163)	321.5 (278.2,367.5)	10.78 (10.72,10.87)
Türkiye	123161 (104949,142079)	309.08 (267.55,355.91)	339742 (290165,391937)	342.72 (294.38,393.33)	10.88 (10.03,10.51)
United Arab Emirates	2910 (2436,3401)	292.99 (250.04,341.49)	41219 (34417,48957)	329.15 (283.49,377.72)	12.34 (10.61,13.38)
Yemen	15714 (13445,18094)	263.54 (226.65,302.44)	52993 (45210,60683)	287.92 (246.68,331.92)	9.25 (8.84,9.75)
**South Asia**	2074360 (1774240,2358850)	296.19 (255.45,336.72)	5405132 (4656883,6153076)	324.2 (280.14,369.04)	9.46 (9.6,9.67)
Bangladesh	157676 (135883,178672)	281.85 (243.79,318.73)	465724 (404057,526279)	306.77 (266.11,346.54)	8.84 (8.73,9.16)

Bhutan	917 (784,1049)	295.2 (256.62,335.7)	2242 (1954,2540)	326.53 (283.84,374.08)	10.61 (10.61,11.43)
India	1723865 (1477512,1963448)	302.9 (261.25,344.42)	4416602 (3806875,5017935)	331.38 (286.04,376.48)	9.4 (9.31,9.49)
Nepal	32779 (28155,37137)	284.69 (246.22,322.6)	82910 (72417,94336)	319.76 (279.61,362.7)	12.32 (12.43,13.56)
Pakistan	159122 (133809,184427)	250.45 (211.9,291.24)	437654 (367264,505132)	282.24 (238.27,326.16)	12.69 (11.99,12.44)
**Southern Sub-Saharan Africa**	96752 (82606,111033)	315.17 (269.79,363.44)	227947 (195149,260792)	338.1 (290.72,388.21)	7.28 (6.82,7.76)
Botswana	1967 (1690,2257)	297.52 (255.96,343.81)	6399 (5492,7336)	334.76 (289.67,386.2)	12.52 (12.33,13.17)
Eswatini	1154 (986,1340)	319.37 (274.58,370.15)	2463 (2115,2821)	343.78 (296.53,394.27)	7.64 (6.52,7.99)
Lesotho	2764 (2361,3211)	295.5 (252.96,344.05)	4074 (3521,4664)	323.02 (279.68,372.27)	9.31 (8.2,10.56)
Namibia	2215 (1905,2545)	295.16 (254.62,338.98)	5396 (4600,6268)	317.84 (271.23,369.61)	7.68 (6.52,9.04)
South Africa	74306 (63379,85286)	320.58 (274.25,369.21)	181939 (156284,208194)	343.76 (295.83,394.13)	7.23 (6.75,7.87)
Zimbabwe	14346 (12302,16455)	297.96 (256.25,344.7)	27675 (23559,31925)	309.77 (265.12,358.16)	3.96 (3.46,3.9)
**Western Sub-Saharan Africa**	310356 (264842,356491)	302.36 (259.52,347.66)	831905 (706430,953808)	326.72 (280.96,375.13)	8.06 (7.9,8.26)
Benin	6757 (5794,7737)	302.6 (259.19,345.95)	22034 (18694,25258)	333.07 (284.78,380.41)	10.07 (9.87,9.96)
Burkina Faso	14567 (12404,16666)	294.48 (252.52,336.81)	36539 (31105,42298)	319.48 (274.65,371.6)	8.49 (8.76,10.33)
Cabo Verde	622 (542,725)	307.86 (264.48,358.26)	1702 (1435,1982)	336.02 (286.9,388.53)	9.15 (8.45,8.48)
Cameroon	17393 (14801,20020)	322.89 (277.77,372.71)	57492 (48938,66138)	344.74 (295.14,395.41)	6.77 (6.09,6.25)
Chad	8855 (7575,10255)	288.96 (247.13,335.69)	22448 (19184,25944)	302.5 (259.8,349.03)	4.69 (3.97,5.13)
Côte d'Ivoire	16563 (14082,19178)	304.63 (263.16,351.41)	50403 (42746,58562)	325.4 (279.86,377)	6.82 (6.35,7.28)
Gambia	1303 (1107,1494)	299.05 (256.02,346.86)	3973 (3370,4554)	325.63 (278.32,375.12)	8.89 (8.15,8.71)
Ghana	23169 (19769,26938)	300.17 (258.05,346.15)	72773 (61958,83998)	337.69 (289.41,389.59)	12.5 (12.15,12.55)
Guinea	10718 (9220,12396)	297.29 (255.59,343.71)	21389 (18325,24510)	315.11 (270.45,360.97)	5.99 (5.02,5.81)
Guinea-Bissau	1417 (1197,1636)	298.26 (253.73,347.69)	3123 (2644,3608)	314.58 (270.12,362.29)	5.47 (4.2,6.46)

Liberia	3907 (3382,4463)	306.96 (265.25,354.38)	10220 (8677,11946)	332.59 (285.96,382.71)	8.35 (7.81,7.99)
Mali	13799 (11711,15932)	291.93 (251.12,336.73)	34432 (29545,39920)	311.58 (267.02,358.42)	6.73 (6.33,6.44)
Mauritania	3506 (3007,4026)	315.1 (270.77,362.96)	8729 (7434,10077)	342.68 (295.8,394.76)	8.75 (8.76,9.24)
Niger	10376 (8821,11914)	290.3 (249.18,333.16)	31537 (26848,36392)	306.87 (264.61,354.24)	5.71 (6.19,6.33)
Nigeria	154398 (132562,177990)	303.57 (261.99,349.88)	392935 (333643,451627)	328.28 (282.57,378.37)	8.14 (7.86,8.14)
Sao Tome and Principe	206 (177,238)	308.5 (264.96,355.14)	485 (415,557)	334.58 (286.17,383.72)	8.45 (8,8.05)
Senegal	11394 (9743,13116)	305.86 (261.6,354.95)	30251 (25636,34872)	324.94 (277.71,375.05)	6.24 (5.66,6.16)
Sierra Leone	6699 (5770,7614)	295.66 (253.38,338.77)	14924 (12772,17111)	314.99 (271.78,361.27)	6.54 (6.64,7.26)
Togo	4696 (3985,5460)	298.99 (257.15,347.22)	16507 (14016,18943)	320.26 (275.08,367.1)	7.11 (5.73,6.97)
**Eastern Sub-Saharan Africa**	252480 (215755,289261)	281.41 (242.27,322.52)	652313 (555382,747784)	299.96 (257.48,343.27)	6.59 (6.28,6.43)
Burundi	7390 (6366,8455)	278.92 (240.04,321.4)	18192 (15396,20934)	283.54 (243.39,328.64)	1.66 (1.4,2.25)
Comoros	674 (577,779)	287.19 (248.74,332.29)	1770 (1511,2039)	303.72 (260.3,350.03)	5.76 (4.65,5.34)
Djibouti	538 (456,618)	278.38 (238.86,320.55)	2792 (2360,3240)	305.45 (263.05,355.23)	9.72 (10.13,10.82)
Eritrea	4294 (3622,4986)	273 (236.27,315.04)	10964 (9328,12626)	287.53 (246.84,329.86)	5.32 (4.47,4.7)
Ethiopia	67012 (56962,77311)	277.07 (238.67,316.55)	159439 (135970,183353)	294.68 (252.17,338.09)	6.36 (5.66,6.8)
Kenya	27982 (23900,32099)	287.81 (247.39,331.66)	91278 (77743,105051)	311.51 (267.23,358.61)	8.23 (8.02,8.13)
Madagascar	16334 (13891,18759)	277.21 (237.35,318.95)	44510 (37631,51409)	288.87 (247.56,333.28)	4.21 (4.3,4.49)
Malawi	13122 (11210,15086)	284.5 (244.85,324.23)	28840 (24494,33110)	304.18 (260.52,349.83)	6.92 (6.4,7.9)
Mozambique	20448 (17455,23329)	280 (240.14,322.66)	43137 (36697,49372)	298.98 (256.13,346.19)	6.78 (6.66,7.29)
Rwanda	9474 (8114,10868)	283.67 (243.68,325.71)	24192 (20590,27821)	301.71 (258.74,347.37)	6.36 (6.18,6.65)
Somalia	9643 (8147,11147)	279.9 (240.07,323.46)	25303 (21447,29156)	289.11 (247.97,332.35)	3.29 (2.75,3.29)
South Sudan	8078 (6939,9349)	280.88 (240.97,324.96)	15818 (13278,18281)	299.05 (256.5,342.94)	6.47 (5.53,6.44)

Uganda	21044 (17916,24075)	279.39 (240.24,320.89)	57144 (48496,65544)	300.15 (257.86,347.36)	7.43 (7.33,8.25)
United Republic of Tanzania	36222 (30892,41616)	287.86 (246.91,331.04)	99374 (85167,115443)	308.44 (266.52,356.13)	7.15 (7.58,7.94)
Zambia	10043 (8566,11545)	288.03 (248.31,331.89)	28993 (24633,33446)	305.74 (261.9,352.6)	6.15 (5.47,6.24)
**Central Sub-Saharan Africa**	79094 (67356,90793)	286.39 (245.77,329.26)	217592 (185701,248119)	297.77 (256.63,338.31)	3.97 (2.75,4.42)
Angola	14096 (11987,16348)	278.46 (239.35,321.06)	48488 (41396,55817)	303.14 (260.9,349.17)	8.86 (8.76,9)
Central African Republic	3980 (3388,4611)	279.21 (240.38,323.05)	8839 (7439,10255)	289.53 (249.51,335.13)	3.7 (3.74,3.8)
Congo	3677 (3112,4236)	294.94 (252.24,338.88)	11717 (9970,13588)	314.59 (271.69,363.49)	6.66 (7.26,7.71)
Democratic Republic of the Congo	54889 (46596,63245)	288.16 (247.73,331.16)	141872 (121356,162084)	293.84 (253.58,335.58)	1.97 (1.33,2.36)
Equatorial Guinea	653 (556,757)	281.98 (242.9,327.18)	2339 (1986,2679)	328.75 (282.88,379.49)	16.59 (15.99,16.46)
Gabon	1801 (1532,2091)	298.26 (254.48,344.14)	4336 (3720,4992)	333.07 (290.16,382.17)	11.67 (11.05,14.02)

Table S3: DALYs cases of Osteoarthritis knee disease in 1990 and 2021 the percentage change in the age-standardised rates (ASRs) per 100,000, by location.

	1990		2021		Percentage change in the ASRs per 100,000
	**No (95% UI)**	**ASRs per 100,000 (95% UI)**	**No (95% UI)**	**ASRs per 100,000 (95% UI)**	
**Global**	5145339 (2507481,9953258)	127.14 (62.17,246.99)	12019070 (5858108,23267858)	137.59 (67.08,266.87)	8.22 (7.9,8.05)
**High-income North America**	482468 (237787,957742)	143.91 (70.69,283.92)	911621 (449630,1807879)	148.92 (73.19,294.55)	3.48 (3.54,3.74)
Canada	24628 (12065,48831)	77.19 (37.83,152.5)	58109 (28731,114079)	87.62 (43.44,170.24)	13.51 (11.63,14.83)
Greenland	31 (15,60)	84.55 (41.86,167.5)	69 (33,134)	94.62 (45.73,182.76)	11.91 (9.11,9.25)
United States of America	457798 (225498,909620)	151.02 (74.13,298.17)	853428 (420640,1694293)	156.32 (76.78,309.62)	3.51 (3.57,3.84)
**Australasia**	31751 (15676,63068)	137.26 (67.72,271.84)	76128 (37728,153473)	154.18 (75.68,309.09)	12.33 (11.75,13.7)
Australia	26596 (13086,52885)	137.84 (67.8,273.27)	64246 (32026,129852)	155.17 (76.55,312.08)	12.57 (12.91,14.2)
New Zealand	5155 (2544,10185)	134.3 (66.21,264.63)	11881 (5739,23580)	149.04 (71.52,294.32)	10.98 (8.02,11.22)
**High-income Asia Pacific**	360521 (174917,701559)	174.98 (85.11,341.43)	722000 (353789,1446533)	180.61 (88.02,356.06)	3.22 (3.42,4.28)
Brunei Darussalam	189 (92,376)	172.54 (83.33,345.57)	721 (348,1382)	186.75 (91.37,362.74)	8.24 (4.97,9.65)
Japan	294380 (143191,572927)	170.73 (83.19,332.69)	516730 (252520,1038663)	172.94 (84.12,338.87)	1.29 (1.12,1.86)
Singapore	4187 (2003,8083)	180.34 (86.99,351.05)	16639 (7934,32771)	189.49 (90.34,372.91)	5.07 (3.85,6.23)
Republic of Korea	61765 (29630,120272)	197.92 (96.25,386.35)	187910 (91977,373887)	199.93 (97.42,398.67)	1.02 (1.22,3.19)
**Western Europe**	702719 (344993,1392177)	126.28 (61.88,248.06)	1119462 (552620,2232285)	133.93 (65.73,263.33)	6.06 (6.16,6.22)
Andorra	73 (36,144)	124.16 (60.92,245.86)	204 (99,406)	133.69 (64.59,266.35)	7.68 (6.02,8.33)
Austria	14228 (6962,28344)	126.03 (61.55,249.93)	22227 (10965,44212)	133.91 (65.83,264.35)	6.25 (5.77,6.95)
Belgium	18380 (8925,36700)	124.42 (60.17,247.59)	27736 (13568,55345)	131.97 (64.77,261.92)	6.07 (5.79,7.65)
Cyprus	992 (488,1947)	119.45	2630 (1307,5124)	129.59	8.49 (7.37,8.54)

		(59.04,234.05)		(64.08,251.31)	
Denmark	9137 (4518,18175)	119.5 (58.85,234.23)	13922 (6826,28168)	128.83 (62.84,261.27)	7.81 (6.78,11.54)
Finland	8807 (4384,17434)	126.59 (62.86,249.24)	14890 (7217,29729)	133.99 (64.33,264.62)	5.85 (2.34,6.17)
France	97176 (47476,191946)	123.22 (60.04,240.88)	162584 (79891,327792)	131.62 (64.49,260.94)	6.82 (7.41,8.33)
Germany	156565 (77308,311069)	127.99 (63.16,252.34)	231584 (114594,458428)	134.22 (66.06,262.59)	4.87 (4.06,4.59)
Greece	18833 (9078,37247)	125.79 (60.91,247.97)	28081 (13784,56505)	134.25 (65.69,267.61)	6.73 (7.85,7.92)
Iceland	351 (172,694)	127.75 (62.62,250.99)	744 (364,1467)	136.89 (67.2,268.07)	7.15 (6.81,7.31)
Ireland	5014 (2457,10022)	125.9 (61.73,250.72)	10195 (5030,20164)	134.37 (66.12,264.26)	6.73 (5.4,7.11)
Israel	5920 (2878,11919)	124.69 (60.39,249.91)	15676 (7704,31340)	134.15 (65.8,266.51)	7.59 (6.64,8.96)
Italy	107085 (52915,211524)	123.85 (61.14,243.49)	167636 (82461,335989)	130.44 (63.53,257.36)	5.32 (3.91,5.7)
Luxembourg	679 (337,1377)	126.72 (62.78,257.06)	1359 (674,2689)	133.32 (65.96,263.44)	5.21 (2.48,5.07)
Malta	533 (266,1054)	124.43 (62.24,246.46)	1174 (577,2331)	133.51 (64.92,266.47)	7.3 (4.31,8.12)
Monaco	84 (41,167)	132.69 (65.63,264.31)	120 (59,238)	139.02 (68.03,272.43)	4.77 (3.07,3.66)
Netherlands	25516 (12402,50214)	131.87 (63.9,257.97)	45063 (21860,92076)	138.94 (67.19,282.57)	5.36 (5.15,9.54)
Norway	7579 (3725,15067)	120.73 (59.02,235.92)	11935 (5873,23859)	129.02 (63.15,255.23)	6.87 (7,8.18)
Portugal	17039 (8321,33912)	123.68 (60.43,245.07)	29502 (14316,59595)	134.19 (65.21,267.76)	8.5 (7.91,9.26)
San Marino	43 (21,85)	128.59 (63.16,252.68)	90 (45,182)	136.2 (67.19,271.18)	5.92 (6.38,7.32)
Spain	67480 (32731,134997)	126.8 (61.43,252.33)	119290 (58113,239202)	134.64 (65.43,268.95)	6.18 (6.51,6.59)
Sweden	13756 (6680,27910)	98.45 (47.81,198.19)	20366 (9790,40597)	106.73 (51.7,214.8)	8.41 (8.14,8.38)
Switzerland	12251 (5979,24388)	124.04 (60.65,244.74)	21202 (10378,42190)	129.08 (62.8,254.19)	4.06 (3.54,3.86)
United Kingdom	114621 (56460,228189)	134.22 (65.93,264.35)	170266 (84003,337772)	143.71 (70.54,281.89)	7.07 (6.64,6.99)
**Southern Latin America**	61860 (30519,121117)	132.85 (65.49,260.41)	126220 (61981,249769)	148.13 (72.64,291.9)	11.5 (10.92,12.09)

Uruguay	5086 (2502,10044)	133.26 (65.37,263.38)	7602 (3788,14814)	149.07 (73.92,289.97)	11.86 (10.1,13.08)
Argentina	42867 (21102,83641)	131.87 (64.81,257.55)	80377 (39368,158658)	146.84 (71.81,289.41)	11.35 (10.8,12.37)
Chile	13904 (6722,27550)	135.77 (65.85,269.09)	38234 (18693,77073)	150.69 (73.39,303.93)	10.99 (11.45,12.95)
**Eastern Europe**	281591 (138411,548173)	100.24 (49.43,195.97)	372649 (183304,722199)	108.31 (53.24,209.35)	8.05 (6.83,7.71)
Belarus	13245 (6472,26018)	101.92 (49.76,200.77)	17264 (8318,34019)	109.69 (52.83,216.39)	7.62 (6.17,7.78)
Estonia	2113 (1020,4122)	103.27 (50.1,201.72)	2727 (1323,5331)	112.49 (54.32,218.33)	8.93 (8.23,8.42)
Latvia	3717 (1826,7249)	103.97 (51.11,202.53)	4041 (2014,8014)	111.88 (55.63,219.71)	7.61 (8.48,8.84)
Lithuania	4657 (2299,9237)	103.12 (50.89,204.98)	5839 (2848,11480)	110.56 (53.62,215.33)	7.21 (5.05,5.36)
Republic of Moldova	4434 (2171,8657)	99.32 (48.9,195.9)	6424 (3145,12500)	109.69 (53.78,213.18)	10.44 (8.82,9.98)
Russian Federation	180978 (88673,350851)	99.62 (49,194.07)	256203 (126319,498011)	108.83 (53.62,211.13)	9.25 (8.79,9.43)
Ukraine	72447 (35302,142316)	101.08 (49.27,199.33)	80151 (39438,154260)	105.89 (52.12,202.91)	4.76 (1.8,5.78)
**Central Europe**	143854 (70890,278718)	95.69 (47.26,186)	215177 (106271,422092)	103.21 (50.75,200.27)	7.86 (7.38,7.67)
Albania	1951 (944,3787)	91.22 (44.58,178.69)	4253 (2042,8595)	99.3 (47.97,200.61)	8.86 (7.6,12.27)
Bosnia and Herzegovina	3955 (1928,7750)	92 (45.14,180.9)	5971 (2935,11572)	99.6 (48.77,192.94)	8.26 (6.66,8.04)
Bulgaria	12290 (5937,23729)	98.29 (47.48,189.85)	13728 (6750,26930)	102.7 (50.41,198.79)	4.49 (4.71,6.17)
Croatia	6023 (2958,11892)	96.25 (47.15,190.95)	8419 (4105,16399)	102.96 (50.42,198.34)	6.97 (3.87,6.94)
Czechia	13040 (6447,25248)	96.38 (47.48,186.09)	20512 (9962,40962)	103.48 (50.37,204.47)	7.37 (6.09,9.88)
Hungary	14369 (7077,28081)	98.84 (48.48,193.09)	19055 (9423,36956)	105.11 (51.7,203.86)	6.34 (5.58,6.64)
Montenegro	628 (309,1211)	98.77 (48.46,191.43)	999 (491,1957)	103 (50.62,201.4)	4.28 (4.46,5.21)
North Macedonia	1826 (891,3578)	94.7 (46.48,186.89)	3393 (1658,6626)	100.67 (49.45,196.81)	6.3 (5.31,6.39)
Poland	41229 (20378,80488)	94.48 (46.75,184.66)	69832 (34359,136543)	103.31 (50.64,200.25)	9.35 (8.32,8.44)
Romania	26849 (13167,52774)	94.77 (46.67,187.23)	36137 (17727,71550)	103.48 (50.64,202.78)	9.19 (8.31,8.51)

Serbia	11294 (5516,21504)	96.52 (47.19,186.17)	16014 (7794,31639)	103.12 (50.21,203.02)	6.84 (6.4,9.05)
Slovakia	5730 (2815,11121)	96.74 (47.49,187.92)	9556 (4709,18597)	103.69 (50.99,200.43)	7.18 (6.66,7.37)
Slovenia	2369 (1149,4716)	96.37 (46.75,192.03)	4179 (2072,8307)	103.56 (51.21,202.39)	7.46 (5.39,9.54)
**Central Asia**	38487 (18666,73649)	81.49 (39.7,157.58)	74350 (36555,143465)	86.71 (42.6,169.71)	6.41 (7.3,7.7)
Armenia	2213 (1081,4257)	79.14 (38.58,153.17)	3722 (1843,7312)	86.74 (42.89,170.72)	9.6 (11.17,11.46)
Azerbaijan	4124 (2003,8076)	81.11 (39.88,160.53)	9708 (4742,18495)	88.42 (43.31,170.71)	9.01 (6.34,8.6)
Georgia	5339 (2608,10222)	84.64 (41.16,161.76)	5020 (2480,9878)	87.14 (42.82,169.53)	2.95 (4.03,4.8)
Kazakhstan	10801 (5193,20651)	84.36 (40.71,162.94)	16847 (8206,33048)	90.33 (44.02,179.02)	7.08 (8.13,9.87)
Kyrgyzstan	2375 (1140,4601)	79.69 (38.5,155.89)	4261 (2026,8187)	83.75 (40.22,161.24)	5.09 (3.43,4.47)
Mongolia	829 (402,1595)	78.35 (38.19,151.98)	2092 (1020,3953)	83.38 (40.98,162.01)	6.42 (6.6,7.31)
Tajikistan	2087 (994,4133)	75.86 (36.2,151.75)	4935 (2417,9778)	78.21 (38.74,156.27)	3.1 (2.98,7.02)
Turkmenistan	1560 (759,3037)	80.99 (39.57,157.96)	3733 (1820,7344)	88.18 (43.22,175.04)	8.88 (9.22,10.81)
Uzbekistan	9159 (4465,17938)	79.51 (38.62,156.43)	24032 (11727,46756)	85.96 (42.01,169.34)	8.11 (8.25,8.78)
**Central Latin America**	113158 (55122,217637)	131.42 (64.27,254.9)	368356 (179925,711864)	143.61 (70.3,278.58)	9.28 (9.29,9.38)
Colombia	23527 (11385,45058)	128.55 (61.96,247.24)	79043 (38116,153867)	142.58 (68.71,277.9)	10.91 (10.89,12.4)
Costa Rica	2357 (1142,4651)	132.93 (64.61,262.42)	8041 (3923,15769)	145.48 (70.95,286.03)	9.44 (9,9.81)
El Salvador	3903 (1905,7522)	130.08 (63.64,250.97)	8819 (4302,17539)	145.01 (70.69,288.1)	11.48 (11.08,14.79)
Guatemala	4462 (2135,8842)	122.05 (59.02,241.98)	15231 (7434,30404)	134.63 (65.58,267.88)	10.31 (10.7,11.11)
Honduras	2650 (1300,5101)	125.32 (61.38,240.79)	9160 (4487,17958)	137.46 (67.67,270.02)	9.69 (10.25,12.14)
Mexico	2461 (1196,4880)	147.54 (71.91,291.01)	5566 (2733,11041)	154.47 (75.57,303.7)	4.7 (4.36,5.09)
Nicaragua	2024 (990,3869)	127.34 (62.35,245.13)	7141 (3503,13903)	140.24 (69,274.93)	10.13 (10.67,12.16)
Panama	1879 (905,3661)	123.81 (59.76,242.05)	6223 (3041,12053)	140.61 (68.7,272.54)	13.57 (12.6,14.96)

Venezuela (Bolivarian Republic of)	13309 (6484,26195)	132.91 (64.96,262.94)	44474 (21700,85825)	143.37 (69.93,277.15)	7.87 (5.4,7.65)
**Andean Latin America**	27640 (13612,53615)	131.2 (64.79,255.6)	88874 (43242,172157)	147.55 (72.02,286.28)	12.46 (11.16,12)
Bolivia (Plurinational State of)	4218 (2051,8362)	126.93 (62.35,251.08)	13606 (6634,26815)	142.87 (70.17,281.93)	12.56 (12.29,12.54)
Ecuador	7494 (3696,14624)	137.45 (67.85,268.19)	25363 (12259,48621)	152.58 (73.79,292.4)	11.01 (8.75,9.03)
Peru	15928 (7860,30926)	129.6 (64.08,252.92)	49906 (24296,97319)	146.4 (71.47,285.33)	12.96 (11.53,12.81)
**Caribbean**	34448 (16791,66909)	131.6 (64.27,255.98)	77063 (38028,151478)	142.84 (70.48,281.03)	8.54 (9.66,9.79)
Antigua and Barbuda	67 (32,132)	131.91 (63.78,256.72)	158 (78,311)	141.53 (69.6,279.91)	7.29 (9.03,9.13)
Bahamas	221 (107,428)	139.64 (67.87,269.85)	639 (314,1229)	148.02 (73.07,288)	6 (6.73,7.66)
Barbados	379 (182,746)	140.14 (67.51,274.13)	745 (359,1459)	148.31 (71.51,289.94)	5.83 (5.77,5.93)
Belize	124 (61,240)	133.72 (65.68,258.22)	471 (230,917)	148.58 (72.5,292.35)	11.11 (10.38,13.22)
Bermuda	94 (46,184)	148.18 (72.65,291.34)	196 (95,391)	154.81 (75.32,307.35)	4.47 (3.68,5.5)
Cuba	13453 (6513,26695)	131.47 (63.65,260.31)	27640 (13579,55736)	144.45 (70.86,292.05)	9.87 (11.33,12.19)
Dominica	78 (38,149)	135.42 (66.32,256.41)	123 (59,236)	143.71 (69.89,277.48)	6.12 (5.38,8.22)
Dominican Republic	4900 (2391,9565)	128.27 (62.62,249.87)	14500 (7069,28749)	142.81 (69.86,283.56)	11.34 (11.56,13.48)
Grenada	87 (43,170)	129.16 (63.41,249.82)	167 (81,333)	140.54 (68.28,281.36)	8.81 (7.68,12.63)
Guyana	498 (242,998)	127.02 (61.7,256.03)	939 (460,1852)	138.72 (67.86,274.75)	9.21 (7.31,9.98)
Haiti	3961 (1934,7670)	118.05 (57.89,229.61)	9660 (4750,18354)	124.28 (61.81,239.32)	5.28 (4.23,6.77)
Jamaica	2268 (1099,4466)	132.05 (63.74,259.51)	4433 (2160,8863)	143.46 (69.88,286.97)	8.64 (9.63,10.58)
Puerto Rico	5266 (2559,10274)	145.89 (70.96,283.53)	10051 (4946,19810)	158.99 (77.74,309.27)	8.98 (9.08,9.55)
Saint Kitts and Nevis	48 (23,93)	135.65 (66.11,261.08)	111 (54,216)	147.7 (72.28,291.17)	8.88 (9.33,11.53)
Saint Lucia	114 (56,224)	131.05 (64.84,257.19)	352 (171,694)	143.48 (69.78,283.36)	9.48 (7.62,10.18)
Saint Vincent and the Grenadines	90 (44,178)	129.15 (62.66,253.02)	206 (100,403)	141.03 (68.78,277.31)	9.2 (9.6,9.77)

Suriname	348 (169,675)	131.82 (63.96,258.14)	944 (466,1810)	144.22 (71.03,278.43)	9.41 (7.86,11.05)
Trinidad and Tobago	1155 (566,2241)	135.7 (66.65,262.31)	2853 (1394,5679)	146.43 (71.6,292.18)	7.91 (7.43,11.39)
United States Virgin Islands	131 (63,255)	144.74 (70.62,282.31)	269 (132,522)	155.25 (76.17,299.19)	7.26 (5.98,7.86)
**Tropical Latin America**	119829 (58969,229561)	126.74 (62.67,245)	369382 (181038,712660)	141.07 (69.25,272.84)	11.31 (10.5,11.36)
Brazil	116896 (57537,223889)	126.66 (62.65,244.8)	360985 (176855,696257)	141.08 (69.23,272.85)	11.38 (10.5,11.46)
Paraguay	2933 (1432,5722)	129.69 (63.52,253.77)	8398 (4150,16404)	140.18 (69.46,274.98)	8.09 (8.36,9.35)
**East Asia**	1390648 (668816,2682918)	151.13 (72.88,291.29)	3677623 (1773767,7079261)	162.47 (78.29,314.12)	7.5 (7.42,7.84)
China	1339566 (644356,2583887)	151.24 (72.96,291.47)	3554153 (1715777,6842994)	162.44 (78.35,314.13)	7.41 (7.39,7.77)
Democratic People's Republic of Korea	26231 (12587,50986)	148.28 (71.75,285.53)	53199 (25426,102393)	154.48 (74.25,298.56)	4.18 (3.48,4.56)
Taiwan (Province of China)	24851 (11783,48933)	148.97 (70.96,294.47)	70271 (33639,136870)	170.98 (81.66,333.52)	14.77 (13.26,15.08)
**Southeast Asia**	252388 (122330,486666)	92.87 (45.45,179.67)	736837 (356755,1411054)	104.35 (50.89,201.33)	12.36 (11.97,12.06)
Cambodia	4482 (2151,8704)	92.91 (44.91,179.13)	13447 (6479,26600)	100.75 (48.88,200.81)	8.44 (8.84,12.1)
Indonesia	98165 (47713,190495)	92.39 (45,178.39)	275146 (132684,526765)	102.61 (49.97,197.86)	11.06 (10.91,11.04)
Lao People's Democratic Republic	2033 (979,3936)	93.09 (45.38,180.88)	5223 (2514,10115)	102.16 (49.49,197.59)	9.74 (9.06,9.24)
Malaysia	9917 (4789,19174)	99.19 (48.16,190.3)	33272 (16079,65244)	111.34 (53.97,217.89)	12.25 (12.06,14.5)
Maldives	91 (44,177)	90.31 (43.86,173.83)	419 (200,815)	104.98 (50.84,202.31)	16.24 (15.91,16.38)
Mauritius	763 (369,1481)	100.54 (48.73,195.43)	2090 (1036,4020)	111.59 (55.51,214.8)	10.99 (9.91,13.91)
Myanmar	22417 (10830,43769)	92.39 (44.91,179.51)	53872 (26738,102576)	103.12 (51.32,197.63)	11.61 (10.09,14.27)
Philippines	26112 (12706,50350)	81.92 (40.04,158.07)	82832 (40462,158988)	93.83 (45.87,179.59)	14.54 (13.61,14.56)
Seychelles	58 (28,114)	105.2 (51.47,205.85)	142 (68,276)	112.46 (54.22,219.88)	6.9 (5.34,6.82)
Sri Lanka	10915 (5295,21158)	94.33 (45.88,182.54)	28744 (14158,55010)	104.27 (51.49,200.02)	10.54 (9.58,12.23)

Thailand	39261 (19076,76952)	101.46 (50.05,200.06)	132566 (63764,257908)	120.3 (57.88,234.65)	18.57 (15.64,17.29)
Timor-Leste	297 (144,585)	90.19 (44.19,178.21)	841 (406,1634)	95.32 (46.14,185.94)	5.69 (4.34,4.41)
Viet Nam	37511 (18251,72616)	93 (45.39,181.17)	107216 (51571,209203)	100.6 (49,196.41)	8.17 (7.95,8.41)
**Oceania**	3889 (1841,7536)	119.61 (57.24,233.97)	10965 (5248,21272)	128.75 (62.39,250.96)	7.64 (7.26,9)
American Samoa	35 (17,69)	141.42 (68.6,277.45)	80 (38,159)	152.3 (73.31,301.89)	7.69 (6.87,8.81)
Cook Islands	18 (9,35)	135.27 (65.62,263.93)	40 (19,77)	154.67 (75.91,304.3)	14.34 (15.3,15.68)
Fiji	516 (248,1000)	127.98 (62.29,248.13)	1217 (596,2330)	144.93 (71.49,278.4)	13.24 (12.2,14.77)
Guam	116 (55,227)	135.64 (65.82,265.53)	320 (157,624)	152.14 (74.43,296.92)	12.16 (11.82,13.08)
Kiribati	50 (24,96)	126.9 (62.27,244)	112 (55,216)	139.85 (68.27,269.4)	10.2 (9.64,10.41)
Marshall Islands	20 (10,38)	119.57 (58.23,225.98)	53 (25,104)	131.8 (63.07,260.89)	10.23 (8.31,15.45)
Micronesia (Federated States of)	63 (30,122)	128.31 (61.81,247.46)	117 (56,224)	141.4 (67.91,267.78)	10.2 (8.21,9.87)
Nauru	6 (3,12)	126.88 (62.16,242.36)	9 (4,18)	144.83 (70.35,281.7)	14.15 (13.18,16.23)
Niue	3 (1,6)	134.82 (64.71,265.88)	3 (2,6)	150.68 (72.51,287.76)	11.76 (8.23,12.05)
Northern Mariana Islands	30 (14,58)	134.16 (65.08,265.08)	89 (43,173)	147.76 (71.27,287.35)	10.14 (8.4,9.51)
Palau	14 (7,27)	134.79 (65.18,262.44)	38 (19,74)	147.83 (72.21,287.35)	9.67 (9.49,10.79)
Papua New Guinea	2303 (1086,4470)	114.97 (54.57,226.46)	7264 (3474,14185)	123.06 (59.44,240.74)	7.04 (6.31,8.92)
Samoa	119 (58,227)	134.29 (65,254.58)	219 (106,426)	143.09 (69.75,277.26)	6.55 (7.31,8.91)
Solomon Islands	177 (85,339)	117.49 (57.54,227.46)	513 (247,1001)	131.46 (63.51,255.93)	11.89 (10.38,12.52)
Tokelau	2 (1,3)	127.14 (62.02,249.92)	2 (1,4)	143.77 (70.83,276.03)	13.08 (10.45,14.21)
Tonga	77 (37,154)	132.73 (64.24,265.25)	121 (59,241)	146.33 (71.63,291.56)	10.25 (9.92,11.5)
Tuvalu	9 (4,17)	127.3 (61.5,247.05)	15 (7,30)	140.5 (68.27,278.79)	10.37 (11.01,12.85)
Vanuatu	83 (40,162)	120.78 (59.04,236.49)	260 (126,502)	134.2 (65.81,261.1)	11.11 (10.41,11.47)

**North Africa and Middle East**	191120 (92640,367986)	108.26 (52.86,210.57)	596794 (290214,1148221)	121.33 (59.3,235.41)	12.07 (11.8,12.18)
Afghanistan	6976 (3404,13566)	96.96 (47.77,190.35)	11622 (5532,22727)	105.83 (51.96,207.18)	9.15 (8.77,8.84)
Algeria	13658 (6638,26825)	106.71 (52.1,209.37)	46474 (22727,90485)	121.61 (59.51,238.67)	13.96 (13.99,14.22)
Bahrain	237 (114,476)	114.99 (55.66,230.23)	1537 (739,2958)	126.03 (61.59,245.21)	9.6 (6.51,10.65)
Egypt	32464 (15559,63981)	111.09 (53.66,220.43)	87257 (42389,173548)	124 (60.23,249.48)	11.62 (12.24,13.18)
Iran (Islamic Republic of)	28360 (13831,54075)	103.01 (50.59,198.93)	94081 (46049,179997)	114.33 (56.35,220.61)	10.99 (10.9,11.39)
Iraq	9253 (4558,17759)	114 (56.03,219.58)	31602 (15422,62091)	119.84 (58.95,234.94)	5.12 (5.21,7)
Jordan	1620 (784,3082)	111.52 (54.28,215.82)	10702 (5239,20354)	125.81 (61.49,243.6)	12.81 (12.87,13.28)
Kuwait	839 (409,1662)	116.81 (56.51,231.36)	4922 (2387,9623)	130.5 (64.01,254.71)	11.72 (10.09,13.27)
Lebanon	2439 (1199,4631)	107.47 (52.73,205.41)	7086 (3490,13692)	121.42 (59.85,233.55)	12.98 (13.5,13.7)
Libya	2242 (1092,4295)	113.83 (55.98,218.17)	7281 (3550,14073)	123.25 (60.64,238.55)	8.28 (8.32,9.34)
Morocco	15303 (7421,29900)	105.07 (51.24,204.98)	42500 (20497,82095)	117.61 (56.81,228.84)	11.93 (10.87,11.64)
Oman	769 (370,1511)	102.02 (49.85,199.97)	3173 (1537,6083)	124.08 (61.38,237.17)	21.62 (18.6,23.13)
Palestine	970 (472,1853)	109.7 (53.42,211.32)	3296 (1589,6374)	116.88 (57.52,226.16)	6.55 (7.02,7.68)
Qatar	192 (92,383)	117.44 (57.49,223.99)	2109 (1012,4122)	130.29 (64.19,247.63)	10.94 (10.55,11.65)
Saudi Arabia	6951 (3314,13195)	108.42 (52.19,208.21)	31919 (15257,62521)	127.17 (62.02,250.77)	17.29 (18.84,20.44)
Sudan	9404 (4557,18194)	98.4 (47.9,191.72)	24475 (12017,47106)	114.81 (56.48,221.5)	16.68 (15.53,17.91)
Syrian Arab Republic	5932 (2875,11318)	108.53 (53.24,209.47)	17350 (8395,32825)	119 (57.84,227.04)	9.65 (8.39,8.64)
Tunisia	5599 (2748,10882)	107.73 (52.96,211.4)	16486 (7940,31816)	119.54 (57.56,232.17)	10.96 (8.69,9.82)
Türkiye	42192 (20395,81566)	116.38 (56.6,227.36)	126271 (61284,245051)	130.19 (63.25,252.87)	11.87 (11.22,11.75)
United Arab Emirates	680 (325,1324)	110.22 (53.78,213.6)	9961 (4799,19487)	123.92 (60.68,242.4)	12.43 (12.83,13.48)
Yemen	4937 (2412,9637)	96.03 (47.21,188.3)	16134 (7868,31295)	105.24 (51.68,203.52)	9.59 (8.08,9.47)

**South Asia**	669709 (326141,1287959)	108.55 (53.23,210.18)	1872013 (913685,3586458)	120.81 (59.17,232.43)	11.29 (10.59,11.16)
Bangladesh	51711 (25008,101213)	104.5 (50.94,204.91)	165130 (80996,320139)	114.67 (56.31,222.46)	9.73 (8.56,10.54)
Bhutan	295 (141,567)	111.65 (54.48,215.24)	788 (387,1565)	124.77 (61.28,246.23)	11.75 (12.48,14.4)
India	554440 (269183,1064069)	111.3 (54.46,215.39)	1540454 (751878,2951122)	123.57 (60.56,237.59)	11.02 (10.31,11.2)
Nepal	10624 (5170,20977)	105.06 (51.38,208.44)	29103 (14182,56926)	119.49 (58.27,234.68)	13.74 (12.59,13.41)
Pakistan	52639 (25729,102844)	89.99 (44.24,175.78)	136538 (65620,267834)	102.1 (49.84,199.58)	13.46 (12.66,13.54)
**Southern Sub-Saharan Africa**	32212 (15730,62021)	115.9 (56.92,225.01)	75140 (36629,144659)	123.64 (60.67,240.41)	6.68 (6.59,6.84)
Botswana	643 (307,1239)	109.93 (52.91,214.52)	1992 (975,3940)	123.98 (60.59,248.1)	12.78 (14.52,15.65)
Eswatini	366 (177,705)	119.76 (58.51,231.04)	765 (380,1510)	127.3 (63.27,252.58)	6.3 (8.14,9.32)
Lesotho	947 (473,1811)	109.22 (54.81,210.34)	1327 (650,2590)	118.07 (58.19,233.04)	8.1 (6.17,10.79)
Namibia	736 (353,1451)	109.26 (52.82,216.56)	1732 (842,3385)	117.45 (57.52,231.29)	7.5 (6.8,8.9)
South Africa	24820 (12118,47792)	117.57 (57.7,228.06)	60816 (29661,117281)	125.33 (61.47,243.88)	6.6 (6.53,6.94)
Zimbabwe	4701 (2286,9092)	110.49 (54.14,215.59)	8507 (4101,16584)	113.71 (55.7,220.1)	2.91 (2.09,2.88)
**Western Sub-Saharan Africa**	102089 (49668,195744)	111.44 (54.56,215.6)	261698 (126482,497450)	121.72 (59.6,234.01)	9.22 (8.54,9.24)
Benin	2259 (1080,4411)	111.44 (53.53,217.93)	6984 (3408,13593)	125.07 (61.82,244.36)	12.23 (12.13,15.49)
Burkina Faso	4888 (2355,9516)	108.49 (52.35,212.5)	11823 (5731,22510)	119.79 (58.26,230.29)	10.42 (8.37,11.29)
Cabo Verde	251 (123,489)	115.08 (56.15,222.38)	594 (289,1151)	127.43 (61.88,247.86)	10.73 (10.2,11.46)
Cameroon	5707 (2765,10896)	120.72 (58.75,231.11)	17951 (8569,34980)	130.11 (62.58,254.99)	7.78 (6.52,10.33)
Chad	3031 (1466,5912)	106.11 (51.56,207.61)	6988 (3409,13443)	111.48 (54.86,216.46)	5.06 (4.26,6.4)
Côte d'Ivoire	4938 (2382,9735)	111.88 (54.46,222.52)	15273 (7401,30189)	122.09 (59.48,241.55)	9.13 (8.55,9.22)
Gambia	412 (201,785)	111.38 (55.06,214.32)	1263 (605,2394)	121.78 (58.9,232.44)	9.34 (6.97,8.45)

Ghana	7406 (3558,14249)	111.67 (54.02,215.93)	23295 (11262,44778)	127.8 (62.24,247.63)	14.44 (14.68,15.22)
Guinea	3717 (1840,7210)	109.9 (54.44,212.81)	6950 (3385,13372)	117.14 (57.38,226.26)	6.59 (5.4,6.32)
Guinea-Bissau	453 (222,870)	109.81 (54.47,211.5)	932 (451,1817)	116.95 (57.15,229.79)	6.5 (4.92,8.65)
Liberia	1318 (634,2527)	112.6 (54.35,216.04)	2963 (1449,5771)	123.29 (60.5,241.95)	9.49 (11.32,11.99)
Mali	4503 (2209,8692)	107.14 (52.75,204.88)	10970 (5293,20862)	115.31 (56.18,220.97)	7.63 (6.5,7.85)
Mauritania	1211 (589,2364)	118.4 (57.85,231.98)	2963 (1417,5710)	129.86 (62.52,251.13)	9.68 (8.07,8.26)
Niger	3199 (1549,6242)	107.11 (52.02,208.68)	10114 (4890,19032)	113.83 (55.26,216.92)	6.27 (3.95,6.23)
Nigeria	51197 (24886,98348)	111.53 (54.51,216.17)	122615 (59424,233522)	121.75 (59.65,234.6)	9.16 (8.53,9.43)
Sao Tome and Principe	76 (37,152)	115.88 (56.6,231.87)	155 (75,303)	126.51 (61.91,248.59)	9.17 (7.21,9.38)
Senegal	3798 (1835,7336)	113.44 (55.29,219.71)	10001 (4860,19126)	121.3 (58.77,232.07)	6.93 (5.63,6.29)
Sierra Leone	2266 (1093,4415)	109.01 (52.82,213.95)	4735 (2272,9169)	117.2 (56.65,228.2)	7.51 (6.66,7.25)
Togo	1457 (706,2805)	110.53 (53.52,211.83)	5125 (2442,9961)	120.19 (57.73,233.64)	8.74 (7.87,10.3)
**Eastern Sub-Saharan Africa**	80099 (38879,153647)	102.41 (50.13,199.17)	200595 (97952,381074)	110.05 (54.14,212.29)	7.46 (6.59,8)
Burundi	2427 (1190,4619)	102.31 (50.09,195.85)	5531 (2654,10555)	104.18 (50.29,200.07)	1.83 (0.4,2.15)
Comoros	219 (107,425)	105.7 (51.83,205.55)	586 (283,1108)	112.46 (54.91,215.07)	6.4 (4.63,5.94)
Djibouti	157 (75,304)	102.75 (49.99,197.28)	832 (404,1615)	113.56 (55.49,223.9)	10.52 (11,13.49)
Eritrea	1243 (588,2460)	99.67 (47.89,193.72)	3228 (1551,6295)	104.94 (50.63,207.61)	5.29 (5.72,7.17)
Ethiopia	20700 (10087,40194)	99.51 (49.09,195.81)	49168 (24094,93721)	106.97 (52.87,205.47)	7.5 (4.93,7.7)
Kenya	8940 (4362,17118)	104.62 (51.39,202.65)	28266 (13707,54094)	113.89 (55.72,220.76)	8.86 (8.43,8.94)
Madagascar	5323 (2571,10251)	101.35 (49.45,197.46)	13393 (6533,25563)	106.28 (51.99,207.17)	4.86 (4.92,5.14)
Malawi	4195 (2016,8074)	104.29 (50.67,200.63)	8934 (4421,17114)	112.56 (55.4,217.43)	7.93 (8.37,9.33)
Mozambique	6460 (3093,12550)	101.58 (49.22,199.02)	13149 (6444,25044)	108.99 (53.6,209.25)	7.29 (5.14,8.9)

Rwanda	3055 (1473,5922)	104.29 (50.67,203.61)	7626 (3725,14853)	111.54 (55.03,214.75)	6.95 (5.47,8.6)
Somalia	2724 (1321,5311)	102.64 (50.57,205.02)	7193 (3493,13865)	105.93 (51.62,208.03)	3.21 (1.47,2.08)
South Sudan	2660 (1284,5079)	101.7 (49.36,195.19)	4728 (2321,9021)	108.57 (53.63,210.57)	6.76 (7.88,8.65)
Uganda	6824 (3321,13180)	102.01 (49.71,200.01)	17661 (8570,34096)	110.69 (54.32,216.01)	8.51 (8,9.27)
United Republic of Tanzania	11934 (5752,22969)	105.63 (51.31,204.52)	31518 (15103,60497)	114.37 (55.06,222.36)	8.27 (7.31,8.72)
Zambia	3179 (1523,6160)	105.64 (50.92,208.6)	8609 (4213,16474)	112.62 (55.14,218.85)	6.61 (4.91,8.29)
**Central Sub-Saharan Africa**	24860 (12133,47848)	104.1 (50.94,202.82)	66124 (32089,127548)	109.23 (53.33,213.55)	4.93 (4.69,5.29)
Angola	4258 (2049,8136)	101.5 (49.6,197.92)	14606 (7070,27998)	111.34 (54.45,215.46)	9.69 (8.86,9.78)
Central African Republic	1223 (603,2410)	100.89 (49.35,198.89)	2595 (1269,4953)	105.18 (51.7,204.41)	4.25 (2.78,4.76)
Congo	1204 (586,2304)	108.56 (52.87,209.68)	3541 (1714,6933)	116.57 (56.92,230.51)	7.38 (7.66,9.93)
Democratic Republic of the Congo	17329 (8438,33566)	104.44 (51.04,204.1)	43277 (20996,84125)	107.62 (52.24,211.9)	3.04 (2.35,3.82)
Equatorial Guinea	207 (99,402)	101.7 (49.27,199.52)	689 (335,1341)	122.09 (60.4,237.39)	20.05 (18.98,22.59)
Gabon	638 (313,1249)	110.35 (54.12,216.44)	1415 (689,2761)	124.28 (60.69,244.7)	12.62 (12.14,13.06)
